# A Modular Mathematical Model of Exercise-Induced Changes in Metabolism, Signaling, and Gene Expression in Human Skeletal Muscle

**DOI:** 10.3390/ijms221910353

**Published:** 2021-09-26

**Authors:** Ilya R. Akberdin, Ilya N. Kiselev, Sergey S. Pintus, Ruslan N. Sharipov, Alexander Yu Vertyshev, Olga L. Vinogradova, Daniil V. Popov, Fedor A. Kolpakov

**Affiliations:** 1 Department of Computational Biology, Scientific Center for Information Technologies and Artificial Intelligence, Sirius University of Science and Technology, 354340 Sochi, Russia; kiselev.in@talantiuspeh.ru (I.N.K.); sspintus@biosoft.ru (S.S.P.); sharipov.rn@talantiuspeh.ru (R.N.S.); kolpakov.fa@talantiuspeh.ru (F.A.K.); 2 BIOSOFT.RU, LLC, 630090 Novosibirsk, Russia; 3 Department of Natural Sciences, Novosibirsk State University, 630090 Novosibirsk, Russia; 4 Federal Research Center Institute of Cytology and Genetics SB RAS, 630090 Novosibirsk, Russia; 5 Laboratory of Bioinformatics, Federal Research Center for Information and Computational Technologies, 633010 Novosibirsk, Russia; 6 JSC “Sites-Tsentr”, 123182 Moscow, Russia; avertyshev@mail.ru; 7 Institute of Biomedical Problems of the Russian Academy of Sciences, 123007 Moscow, Russia; microgravity@mail.ru

**Keywords:** mathematical model, skeletal muscle, physical exercise, Ca^2+^-dependent signaling pathway, transcriptome, RNA sequencing, regulation of expression, BioUML

## Abstract

Skeletal muscle is the principal contributor to exercise-induced changes in human metabolism. Strikingly, although it has been demonstrated that a lot of metabolites accumulating in blood and human skeletal muscle during an exercise activate different signaling pathways and induce the expression of many genes in working muscle fibres, the systematic understanding of signaling–metabolic pathway interrelations with downstream genetic regulation in the skeletal muscle is still elusive. Herein, a physiologically based computational model of skeletal muscle comprising energy metabolism, Ca^2+^, and AMPK (AMP-dependent protein kinase) signaling pathways and the expression regulation of genes with early and delayed responses was developed based on a modular modeling approach and included 171 differential equations and more than 640 parameters. The integrated modular model validated on diverse including original experimental data and different exercise modes provides a comprehensive in silico platform in order to decipher and track cause–effect relationships between metabolic, signaling, and gene expression levels in skeletal muscle.

## 1. Introduction

Skeletal muscle tissue comprises about 40% of total body mass in lean adult humans and plays a crucial role in the control of whole-body metabolism and exercise tolerance. Regular low-intensity exercise (aerobic or endurance training) strongly increases vascular and mitochondrial density and oxidative capacity, improving fat and carbohydrate metabolism. These adaptations lead to an enhancement of muscle endurance performance and reduce the risk associated with the morbidity and premature mortality of chronic cardiovascular and metabolic diseases [1,2].

Acute aerobic exercise induces significant metabolic changes in the working skeletal muscle, which in turn activate numerous signaling molecules. Changes in the content of Ca^2+^ ions in skeletal muscle play a fundamental role in the regulation of the activity of contractile proteins and enzymes involved in energy metabolism. In addition, a contraction-induced increase in the content of Ca^2+^ ions in the myoplasm significantly affects the activation of some signaling proteins: Ca^2+^/calmodulin-dependent kinases (CaMKs), calcineurin, Ca^2+^-dependent protein kinase C, etc. [3]. Increasing the intensity of contractile activity more than 50% of maximal pulmonary O_2_ consumption rate (V′O2_max_) induces a linear increase in the activity of AMPK, the key energy sensor of the cell activated by an increase in the AMP/ATP ratio, Ca^2+^-dependent kinase CaMKKII, and a decrease in the level of muscle glycogen [4]. In muscle cells, activated AMPK changes the level of phosphorylation of several dozens of different signaling proteins [5,6,7]. Thus, Ca^2+^ and AMPK play a key role in the regulation of various intracellular signaling cascades, as well as the gene expression induced by exercise.

Dramatic changes in the expression of hundreds of genes were observed during the first hours of recovery after acute intensive aerobic exercise in untrained skeletal muscle [8,9,10] as well as in muscle adapted to regular exercise training [8,10,11]. These changes are associated with muscle contraction per se and with system factors, e.g., humoral factors, neuronal activity, feeding mode, and circadian rhythms. On the basis of the analysis of differentially expressed genes between exercised and contralateral non-exercised vastus lateralis muscle, the contractile activity-specific transcriptome responses at 1 and 4 h after the one-legged exercise were identified in our previous study [12]. It was shown that the most enriched biological process for the transcriptome response is transcription regulation, i.e., an increase in the expression of genes encoding transcription factors and co-activators. The study demonstrated that genes encoding transcription factors such as *NR4A*, *AP-1,* and *EGR1* were actively expressed 1 h after the termination of the exercise, while other transcription regulators such as *PPARGC1A*, *ESRRG,* and *VGLL2* were highly expressed at 4 h. Both sets of transcription factors modulate muscle metabolism. We suggest that gene expression in early and late stages of the recovery after the termination of the exercise can be regulated in different ways [13]. Obviously, these molecular mechanisms are complex, but we assume that each considered gene has a constitutive transcription rate independent of the presence of considered transcription factors, while fine-tuning regulation by them ensures diverse expression dynamics of genes with early and delayed responses to an exercise and recovery. For example, the activation of CREB- and CRTC-like transcription factors by the upstream Ca^2+^-dependent signaling pathway enables the expression increase of early response genes such as *NR4A2, NR4A3*, while the transcription activation of genes with delayed response such as *PPARGC1A* is provided through initial transcription regulation by the same CREB- and CRTC-like factors and translation of the X factor, which is an intermediate regulator. Moreover, conducted bioinformatics analysis of the transcriptomics [12] and ChIP-seq data from the GTRD database revealed potential candidates for this X factor regulating expression of the *PPARGC1A* gene.

It is worth noting that although advancement in the development of high-throughput experimental techniques and generation of diverse omics data for human skeletal muscle during endurance exercise enabled us to unveil key participants of the cellular response and adaptation to stress/various stimuli [8,9,10,11,12], the systematic understanding of signaling–metabolic pathway relationships with downstream genetic regulation in exercising skeletal muscle is still elusive. Detailed mechanistic and multiscale mathematical models have been constructed to provide a powerful in silico tool enabling quantitative investigation of the activation of metabolic pathways during an exercise in skeletal muscle [14,15,16]. Here, we propose a modular model of exercise-induced changes in metabolism, signaling, and gene expression in human skeletal muscle. The model includes different compartments (blood, muscle fibers with cytosol and mitochondria) and allows one to quantitatively interrogate dynamic changes of metabolic and Ca^2+^- and AMPK-dependent signaling pathways in response to aerobic cycling or knee extension exercises of various intensity in slow- and fast-twitch vastus lateralis muscle fibres (type I and II, respectively), as well as downstream regulation of genes with early and delayed responses in a whole/mixed fiber type skeletal muscle.

The model modules are hierarchically organized and presented according to metabolic, signaling, and gene expression levels. To build the model, we used the BioUML platform [17] that is designed for modular modelling of complex biological systems. The effectiveness of both this approach and the BioUML platform was previously confirmed by the development of complex modular models of apoptosis [18] and the cardio-vascular system [19].

## 2. Materials and Methods

This section consists of two subsections. Firstly, the suggested approach for modular construction of complex biological models, their reproducibility, and practical applications using the BioUML platform are described. Afterwards, the main points of the integrated model construction are presented.

### 2.1. BioUML Platform

BioUML (Biological Universal Modeling Language, https://ict.biouml.org accessed on 8 June 2021) [17] is an integrated platform for modeling and analysis of complex biological systems. It supports main standards in systems biology, modular and visual modelling, fast simulation, parameter estimation, and a number of numerical methods, as well as integration with Jupyter Notebook for reproducible research. Together, these cover all needs for modeling complex biological systems.

#### 2.1.1. Systems Biology Standards

It is very important to provide interoperability and reproducibility of mathematical models of complex biological systems [20,21]. For this purpose, the BioUML platform supports the main standards used in systems biology.

- SBML—Systems Biology Markup Language [22] serves for a formal description of mathematical models. BioUML supports all versions of SBML from l1v2 to the latest l3v2, including “comp” [23].
- SBGN—Systems Biology Graphical Notation [24] is used for visual description of model elements (complexes, compartments, molecule types, reactions, etc.). BioUML completely supports SBGN Process Description diagrams and uses them to visually represent SBML models. We also support the XML markup language SBGN-ML (https://github.com/sbgn/sbgn/wiki/SBGN_ML accessed on 8 June 2021), which facilitates the exchange of SBGN diagrams between tools.
- Antimony—human-readable text format, which supports most of the SBML features [25]. BioUML automatically processes it into an SBML diagram in SBGN notation. BioUML supports import and export into antimony format.

However, these standards are not sufficient for some tasks. Thus, we suggest extension of the SBGN Process Description diagram type and Antimony format and demonstrate how they can improve the construction of complex biological models using visual modelling. These extensions supported by the BioUML platform will be described below.

#### 2.1.2. Visual Modelling

Representation of investigated systems as graphical diagrams by means of software supporting visual modeling can significantly facilitate the procedures of the model reconstruction.

Following a paradigm of visual modelling, a user creates mathematical models as visual diagrams. Each component of the diagram corresponds to a particular mathematical object of the model (variable, reaction, metabolite, equation, etc.). Users may additionally edit those elements by changing their properties (i.e., initial value of a variable, kinetic law for the reaction, etc.). Based on this visual representation as well as on defined properties of diagram elements, BioUML automatically generates a program code that is employed to simulate the model dynamics. The current BioUML version generates highly optimized Java code and uses its own state-of-the-art simulation engines.

#### 2.1.3. SBGN Process Diagrams Extension

Graphical notation is a crucial component of visual modeling that allows one to formally and completely build a model. A visual model can be presented by some types of diagrams enabling the description of diverse aspects of the structure and function of a complex system with different levels of details. This formal graphical representation is a basis for automatic code generation by specialized tools to simulate the model.

We devised an extension (Table 1) for the Process Diagram type from the SBGN standard [24] to provide the possibility of graphical representation of mathematical elements used in SBML format: equations, events, functions, and constraints [22]. We also added glyphs to represent tabular data that are used for defining the dynamics of the mathematical variables of the model. Tabular data may be translated into spline curves or a constant piecewise function. Furthermore, tabular data may be used, for example, for defining experimental conditions—training regimen for physical exercises.

Although SBGN notation already has tag elements that denote the module interface (ports in SBML terminology), in our diagrams we have three different types of ports (see below).

#### 2.1.4. Modular Diagrams

Modularity could be considered a principle of biological organization [26,27]. Therefore, a modular approach to the modeling of complex biochemical systems has been actively developing in the last decades [28,29].

In the framework of a modular approach, the investigated system is viewed as a set of interconnected subsystems (modules). Each module is a mathematical model that can be considered and simulated independently. Integration of these modules constitutes a more complex model of the whole system. Modules may leverage different mathematical formalisms and scales. They can be created, validated, and improved independently and may be viewed as replaceable parts.

For visual modelling of modular models, we developed a special diagram type that allows us to specify connections between modules. For this purpose, each module specifies variables that can be used to connect it with other modules. This subset of variables is called the module interface and is represented by ports (Table 2).

Ports can be of three types:

- Input—mathematical variable associated with input ports that is calculated outside of the module and used in the module.
- Output—mathematical variable associated with contact ports can be modified inside the module as well as outside (e.g., using differential equations).
- Contact—mathematical variable associated with output ports calculated inside the module and may be used in other modules. In other words, it is a shared variable that can be simultaneously changed by several modules.

Besides this, modular diagrams can include all mathematical elements and tabular data suggested in Table 1.

#### 2.1.5. Visual Modular Modelling

**Figure 1:**
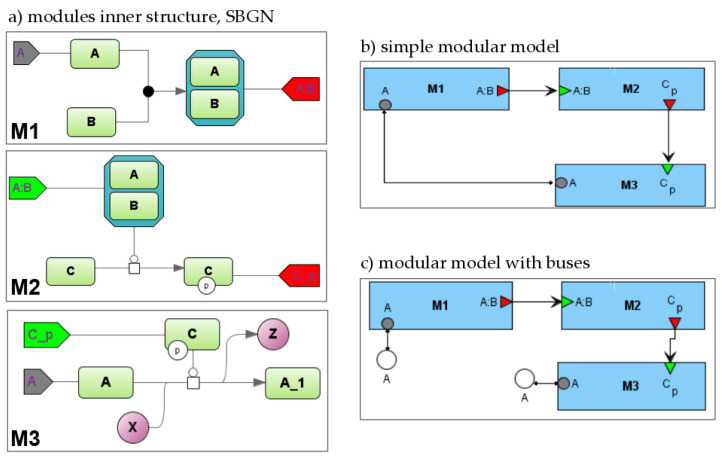
Modular model example. (**a**)—inner structure of modules (SBGN). M1-M3 designate corresponding modules; two green rectangles A and B in M1 correspond to molecules A and B which form the complex A:B, while grey and red pentagons in M1 designate contact port for A and output port for A:B, respectively; two green rectangles C and C_p in M2 correspond to protein C and phosphorylated form of the protein, while green and red pentagons in M2 designate input port for A:B and output port for C_p, respectively; two green rectangles A and A_1 in M3 correspond to molecules A and A_1, while green and grey pentagons in M3 designate input port for C_p and contact port for A, respectively; two purple circles X and Z in M3 mean the additional substrate and product of the bimolecular reaction, correspondingly that is catalyzed by phosphorylated form of the protein C (green rectangle), (**b**,**c**)—modular diagram in two equivalent variants: without or with buses (white circles). Buses serve for cosmetic purposes only.

Module ports are used on two levels (Figure 1):

- when creating model that will be used as a part of another model (i.e., module), a modeler specifies the module inputs, outputs, and contacts and links them to corresponding module entities or variables;
- on a modular diagram, a modeler links several modules together using previously defined ports.

Let us consider a simple example demonstrating this approach (Figure 1). First, we will develop a simple module M1 that consists of one biochemical reaction where two molecules A and B form the complex A:B. We are suggesting that the concentration of entity A can be changed in other reactions due to participation in other modules. To specify this, we will create port A of type “contact” (grey pentagon). The concentration of the A:B complex is solely defined in module M1, and we will create a port A:B of type “output” (red pentagon) that is represented as input in module M2.

Then, we will create module M2 that will also include one reaction where complex A:B catalyzes the phosphorylation of protein C (C{p}). Herein, we will define port A:B as input (green pentagon) for this module and port C{p} as output.

Module M3 also comprises one bimolecular reaction where C{p} catalyzes the transformation of A into A_1. X and Z are chemical substances that are the reactant and product, respectively. Similarly, we will specify C{p} as input, and A port will be a contact while the A concentration is also changed in the reaction from module M1.

Finally, let us form these three modules into a modular model (Figure 1b,c). We will connect corresponding ports to each other (Figure 1b).

- inputs and outputs: A:B for M1 and M2, C{p} for M2 and M3; this is a directed connection so it is depicted by an arrow;
- contacts—A for M1 and M3; this is an undirected connection while concentration A is changed simultaneously by two reactions from these modules and so it is depicted by the line.

More complex modular diagrams may contain a large number of connections that form many intersections. To skip this intersection, we introduce the concept of a bus (white circle in Figure 1c): a port is connected to the named bus, and a diagram may contain several clones of such a bus. Figure 1c demonstrates how the connection of two A ports can be replaced by a connection with two clones of bus A.

Numerical calculations for modular models may be performed in two ways:

- Flattening—a modular model may be transformed into a non-modular model by aggregating all elements of all modules with automatic resolving of established connections between variables [18].
- Agent-based simulation. Each module is simulated independently with its own simulator and formalism. The implemented scheduler coordinates the interactions by sending and receiving the numerical values of connected variables [19].

To simulate the presented integrated modular model, we employ a flattening approach while all modules use the same mathematical formalism and contain sets of ordinary differential equations (ODEs) and discrete events (i.e., hybrid models). The BioUML platform automatically transforms the modular model into a “flat” hybrid model with the same formalism by aggregating all equations and events from all modules and resolving connections. For more details, see [18,19].

#### 2.1.6. Antimony—Extension and Synchronization with Visual Depiction

Antimony provides a convenient human-readable text format that supports most of the SBML features. Herein, we suggest an extension for the Antimony format to specify reaction components to which type of SBGN Process Diagram they correspond, as well as some other properties.

The suggested format is as follows:

@entity_id.property = value

The proposed extension is quite similar to the idea of annotations in SBML format where SBML-enabled software can store any auxiliary information. Similar to Java annotations, we suggest sign @ for this purpose.

Currently, the BioUML platform supports the following properties in annotations:

- sbgnType—defines the SBGN entity type (unspecified, macromolecule, nucleic acid feature, perturbing agent, simple chemical or complex). All those entities correspond to mathematical variables in the model.
- sbgnViewTitle—defines additional properties of an SBGN entity such as whether it is multimer if it has units of information or state variables. If an entity is a complex, it also defines the inner elements of the said complex. We used Transpath conventions to denote entities and complexes in text formats. Here are some examples:
  ○ Complex comprising two entities A and B is denoted as “A:B”.
  ○ Entity A with state variable p (phosphorylated) is denoted as “A{p}”.
  ○ Multimer entity A is denoted as “(A)3”.
  ○ A more advanced example: “(A{p})3:B{r}{p}:C”.

More information can be found in part 2.6 of Transpath documentation at https://genexplain.com/wp-content/uploads/2017/04/TRANSPATH-Documentation_2012.2.pdf (accessed on 8 June 2021).

Depending on the context/tasks, it can be more suitable to present a model of a biological system either as a graph using the extended version of the SBGN Process diagram type or as a program code using Antimony language.

**Figure 2:**
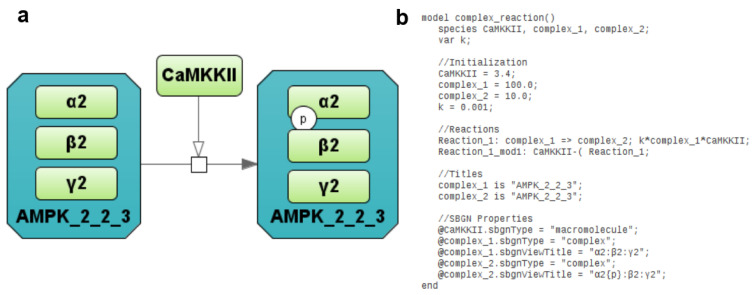
Representation of the simple chemical reaction (**a**) using the SBGN Process Diagram type and Antimony with annotations (**b**). Three green rectangles α2, β2, γ2 inside the dark green octagon AMPK_2_2_3 designate corresponding subunits of the AMPK, while green rectangle CAMKKII mean the kinase catalyzing the phosphorylation reaction.

Antimony+ and PD+ are seamlessly integrated in the frame of the BioUML platform. Due to this integration, a user can simultaneously view and edit textual and graphical representations of a biological system model. Figure 2 demonstrates how the chemical reaction is represented using SBGN Process Diagram Type (2a) and extended Antimony format (2b).

It is noteworthy that if a user edits textual model representation then graphical representation is updated synchronously by the BioUML platform and vice versa. Similarly, if a user selects some object on a diagram, then corresponding text items are highlighted in the text document and vice versa.

#### 2.1.7. Model Simulations

Numerical solutions of the model represented by a system of ordinary differential equations have been obtained on the basis of the VODE method [30] using a JVode simulation engine implemented in the BioUML tool [17]. Each submodule of the modular model can be represented as an independent SBML file [22], while the integrated modular model can be exported as a COMBINE archive [31] to use the model and reproduce simulations, resulting in alternative software supporting current standards of the systems biology.

#### 2.1.8. Jupyter Notebook

**Figure 3:**
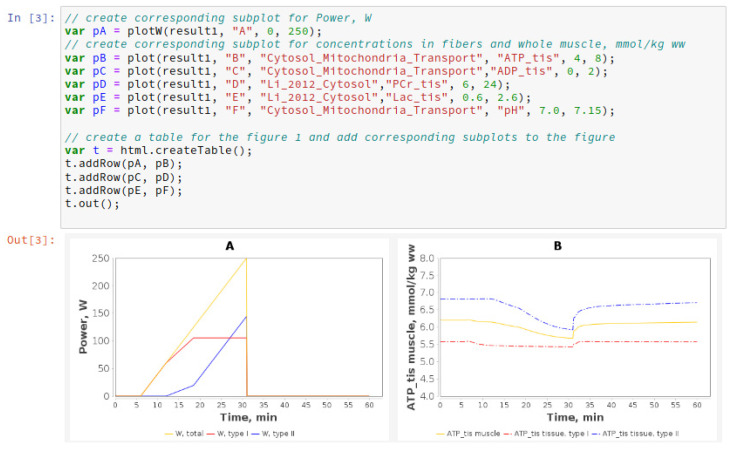
A fragment of Jupyter notebook reproducing the results presented in the current study: (**A**) Exercise power and fiber recruitment pattern: total power (orange), power generated by type I (red) and II (blue) fibers; (**B**) ATP concentration in type I (red, dotted) and II (blue, dotted) fibers and in the muscle tissue (orange, solid) during the incremental ramp exercise until exhaustion.

BioUML is integrated with Jupyter (https://jupyter.org, accessed on 8 June 2021) for interactive data and model analysis as well as an essential and user-friendly tool for the reproducibility of the simulation results (Figure 3). The notebook that provides reproducibility of results presented in the article can be started on the BioUML server as well as using Binder technology.

### 2.2. Integrated Modular Model

#### 2.2.1. The Model Overview

**Figure 4:**
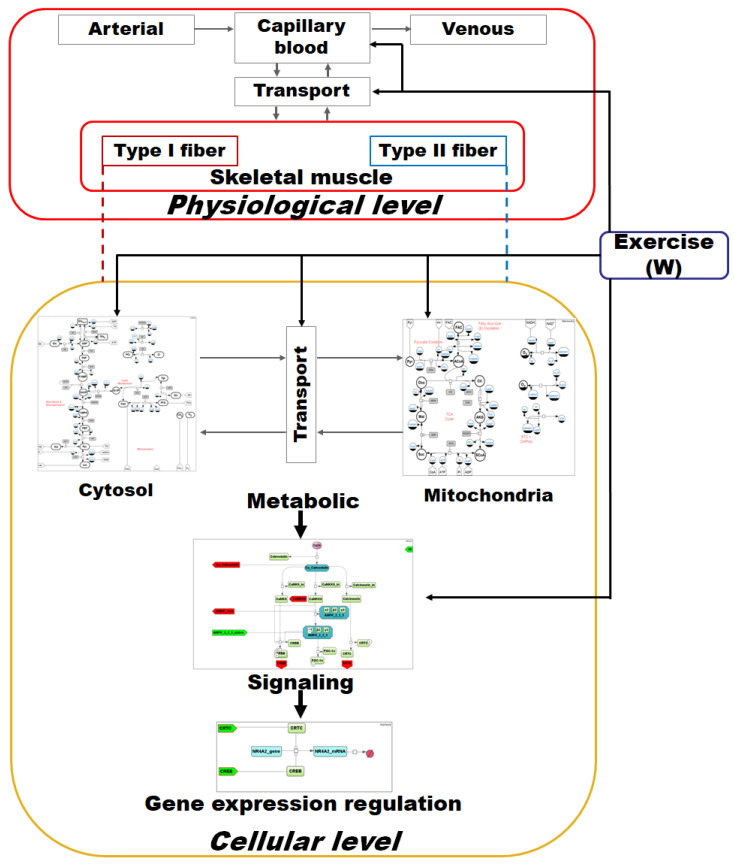
General structure of the integrated modular model linking metabolism, Ca^2+^-dependent signaling transduction, and regulation of gene expression in human skeletal muscle. Grey arrows on the physiological and cellular levels represent transport reactions, red and blue dashed lines from each fiber type to the cellular level indicate duplicated submodules for the corresponding fiber type, while black arrows indicate activation mechanisms related to an exercise response in the corresponding module. The metabolic sublevel consists of submodules “Cytosol” and “Mitochondria”, which in turn contain equations describing enzymatic reactions inside the certain compartment as well as of the Transport submodule comprising transport reactions between them. The signaling sublevel includes Ca^2+^- and AMPK-dependent signaling pathways, while the Gene expression regulation sublevel incorporates submodules describing the expression regulation of genes with early and delayed responses. For a detailed description of all modules and diagrams, see the Supplementary Material.

The general structure of the developed model linking metabolism, Ca^2+^-dependent signaling transduction, and regulation of gene expression in human skeletal muscle is demonstrated in Figure 4.

The model has a hierarchical structure. At the top level, the model describes the physiology of capillary blood flow through muscles during exercise to provide oxygen and substrate delivery and metabolite removal from the skeletal muscle. It is worth noting that many physiological details are lumped in the current version of the model (e.g., cardiorespiratory system elements), and the dynamic change of the capillary blood flow elicited by the exercise is considered a linear function of the exercise intensity [32]. In the muscle model, we consider Type I and Type II fibers. Their modules have the same structure but differ in some parameter values.

On the next level (cellular level), we consider biological processes that occur in human skeletal muscle cells. These processes can also be divided into three sublevels that are described by the corresponding modules of the model:

- Metabolic—the main metabolic processes that occur in the skeletal muscle during physical exercises: glycolysis, glycogenolysis, tricarboxylic acid cycle, β-oxidation, and oxidative phosphorylation. This part of the model is based on a detailed mathematical model of muscle metabolism developed by Li and coauthors [32]. We have redesigned this model according to the methodology described above and changed some model parameters to reproduce more experimental data (see below).
- Signalling— the main signal transduction pathways that are activated by physical exercises are related to Ca^2+^-dependent signaling [33] and AMPK activation [34]. For each of them we developed a special module.
- Gene expression regulation—changes in gene expression were divided into early (up to 1–3 h after exercises) and late (3–6 h after exercises) responses. We selected the most well-known genes for each group—*NR4A2* and *NR4A3* for the first group and *PPRGC1A* for the second as described in the “Gene expression level” section. The corresponding modules that describe the expression of these genes have also been developed.

Oxygen delivery and metabolite transport between cellular compartments (mitochondria and cytoplasm) as well as between muscle cells and capillary blood are very important parts of the model. Therefore, we developed special modules considering all these transport processes.

An activation mechanism that enhances energy metabolism via transport and reaction fluxes due to physical exercise was harnessed as the stress function depending on the general work rate parameter [32]:

$$Function\left(W\right)=1+\alpha_{i}\times W\times \left(1-e^{\frac{t_{start}-t}{\tau_{i}}}\right)$$

where $\alpha_{i}$ is the activation coefficient, W is the work rate value, $\tau_{i}$ indicates the time constant of changes in the metabolic reaction rates in response to exercise, and $t_{start}$ is the simulation time when an exercise is started. The work rate parameter defines the power of the physical exercise and varies depending on the mode of the exercise. In our model, the muscle volume was 5 L, which corresponds to that involved in exercise using a cycling ergometer [14,32].

All details on the general description of each module, representation of the module diagram using extended SBGN Process Description notation, corresponding Antimony code for the module as well as reaction rates and ordinary differential equations to describe the species concentrations, the module parameters, their values, and references to the literature from which they were extracted are presented in the Supplementary Materials.

Overall, the model includes:

- 25 modules;
- 238 species;
- 185 reactions;
- 171 ordinary differential equations;
- 647 parameters.

#### 2.2.2. Physiological (or Organism) Level

**Figure 5:**
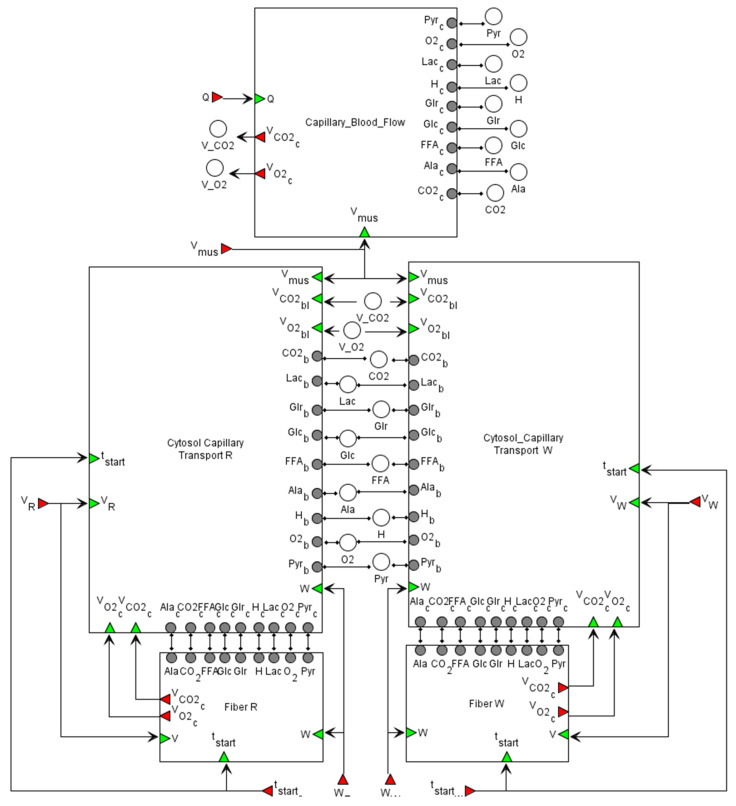
The modular representation of the integrated model at the physiological level. The model comprises five modules: “Capillary_Blood_Flow” to describe flow processes in the blood; “Cytosol_Capillary Transport R” and “Cytosol_Capillary Transport W” modules representing transport reactions between capillary blood and the muscle tissue, “Fiber R” and “Fiber W” modules where metabolic, signaling, and gene expression regulation processes are considered. All details for each submodule and zoomed-in subfigures are in the Supplementary Material.

On this level (Figure 5), we model capillary blood flow for oxygen and substrate delivery to the muscle and for removal of metabolites produced by the muscle including: CO_2_—carbon dioxide; O_2_—oxygen; Lac—lactate; Ala—alanine; Pyr—pyruvate; H—hydrogen; Glr—glycerol; Glc—glucose; FFA—free fatty acid.

It should be noted that those species are present in six different modules and have different subscripts. We explain this using the example of CO_2_. In the module “Capillary_Blood_Flow”, $CO_{2c}$ is the concentration of CO_2_ in the capillary blood. It is connected via connections and buses with $CO_{2b}$ in modules “Cytosol_Capillary Transport R” and “Cytosol_Capillary Transport W”, where $CO_{2b}$ is also the CO_2_ concentration in the capillary blood. In those modules, transport of CO_2_ from blood to the muscle tissue is presented, where $CO_{2c}$ is the concentration in the tissue. Finally, $CO_{2c}$ is connected with the CO_2_ variables in the “Fiber R” and “Fiber W” modules (corresponding to Type I and Type II fibers, respectively), where the metabolism of CO_2_ in tissues is considered. Bus elements (white circles in Figure 5) are used to prevent too many intersections between connections.

Skeletal muscle volume ($V_{mus}$) is represented by the sum of the effective volumes of blood ($V_{bl}$): $V_{mus}=V_{tis}+V_{bl}$—the skeletal muscle volume (5 kg w.w.). In skeletal muscle, recruitment of muscle fibers due to the transition from rest to exercise enhances active muscle volume and blood flow. According to the original metabolic model [32], we also assume that these physiological variables dynamically change as linear functions of the work rate parameter or power of the physical exercise:

$$V_{mus}=V_{mus_{0}}\times (1+\alpha_{i}\times W\times \left(1-e^{\frac{t_{start}-t}{\tau_{V}}})\right)$$

$$Q=Q_{0}\times (1+\alpha_{i}\times W\times \left(1-e^{\frac{t_{start}-t}{\tau_{Q}}})\right)$$

where Q is the blood flow, $Q_{0}$ = 0.9 L/min is the muscle blood flow at rest for two legs; $V_{mus_{0}}$ is the skeletal muscle volume at rest (5 kg w.w.); $\alpha_{i}$ is the activation coefficient; while $\tau_{V}=\tau_{Q}=0.4$ min is the time constant of the muscle volume and blood flow changes, respectively, in response to exercise; and $t_{start}$ is the simulation time when an exercise is started [32].

The muscle consists of two compartments (modules) that correspond to Type I and Type II fibers. They have the same structure but differ in some parameters (see details in corresponding tables of the Supplementary Materials).

#### 2.2.3. Transport Level

The inter-compartmental metabolite transport is described as passive or facilitated (carrier mediated) fluxes according to the original paper [32]. By analogy with metabolic rates, all transport flux equations are multiplied by a linear function to consider the exercise effect on transport processes. The basic transport flux equation for passive (superscript *p*) diffusion of species *i* between the blood and cytosol is:

$$T_{bl\langle -\rangle cyt,type,\;i}^{p}=\lambda_{bl\langle -\rangle cyt,type,\;i}\times \left(C_{bl,\;i}-C_{cyt,type,i}\right)\times \left(1+\alpha_{i}\times W\times \left(1-e^{\frac{t_{start}-t}{\tau_{i}}}\right)\right)$$

where $\lambda_{bl\langle -\rangle cyt,type,\;i}$ is the permeability-surface area coefficient, $C_{bl,\;i}$ and $C_{cyt,type,i}$ are concentrations of species *i* in the blood and cytosol, respectively; $i\in (CO_{2},O_{2},\;Ala,\;Glr$*)* and type∈(type I fiber,type II fiber*)*, while for facilitated (superscript *f*) transport:

$$\begin{array}{c} T_{bl\langle -\rangle cyt,\;type,i}^{f}=R_{max_{transport}bl\langle -\rangle cyt,\;type,i}\times \left(\frac{C_{bl,\;i}}{K_{M_{bl\langle -\rangle cyt,i}}+C_{bl,\;i}}-\frac{C_{cyt,type,i}}{K_{M_{bl\langle -\rangle cyt,i}+}C_{cyt,type,i}}\right)\\ \times \left(1+\alpha_{i}\times W\times \left(1-e^{\frac{t_{start}-t}{\tau_{i}}}\right)\right) \end{array}$$

where $R_{max_{transport}cyt\langle -\rangle mit,\;type,i}$ is the maximal flux rate for facilitated transport, $C_{bl,\;i}$ and $C_{cyt,type,i}$ are concentrations of species *i* in the blood and cytosol, respectively; $i\in (Glc,Pyr,Lac,\;FFA,\;H^{+}$*)* and type∈(type I fiber,type II fiber*).*

The basic transport flux equation for passive (superscript *p*) diffusion of species *i* between the cytosol and mitochondria is:

$$T_{cyt\langle -\rangle mit,type,\;i}^{p}=\lambda_{cyt\langle -\rangle mit,type,\;i}\times \left(C_{cyt,type,\;i}-C_{mit,type,i}\right)*\left(1+\alpha_{i}\times W\times \left(1-e^{\frac{t_{start}-t}{\tau_{i}}}\right)\right)$$

where $\lambda_{cyt\langle -\rangle mit,type,\;i}$ is the permeability-surface area coefficient, $C_{cyt,type,\;i}$ and $C_{mit,type,i}$ are concentrations of species *i* in cytosol and mitochondria, respectively; $i\in (CO_{2},O_{2}$) and type∈(type I fiber,type II fiber*),* while for facilitated (superscript *f*) transport:

$$\begin{array}{c} T_{cyt<\to mit,\;type,i}^{f}=R_{max_{transport}cyt<\to mit,\;type,i}\\ \times \left(\frac{C_{cyt,type,\;i}}{K_{M_{cyt\langle -\rangle mit,i}}+C_{cyt,type,\;i}}-\frac{C_{mit,type,i}}{K_{M_{cyt\langle -\rangle mit,i}+}C_{mit,type,i}}\right)\\ \times \left(1+\alpha_{i}\times W\times \left(1-e^{\frac{t_{start}-t}{\tau_{i}}}\right)\right) \end{array}$$

where $R_{max_{transport}cyt\langle -\rangle mit,\;type,i}$ is the maximal flux rate for facilitated transport, $C_{cyt,type,\;i}$ and $C_{mit,type,i}$ are concentrations of species *i* in the cytosol and mitochondria, respectively; $i\in (H^{+},Pyr,FAC,CoA,P_{i}$*)* and type∈(type I fiber,type II fiber*)*.

#### 2.2.4. Cellular (Metabolic) Level

**Figure 6:**
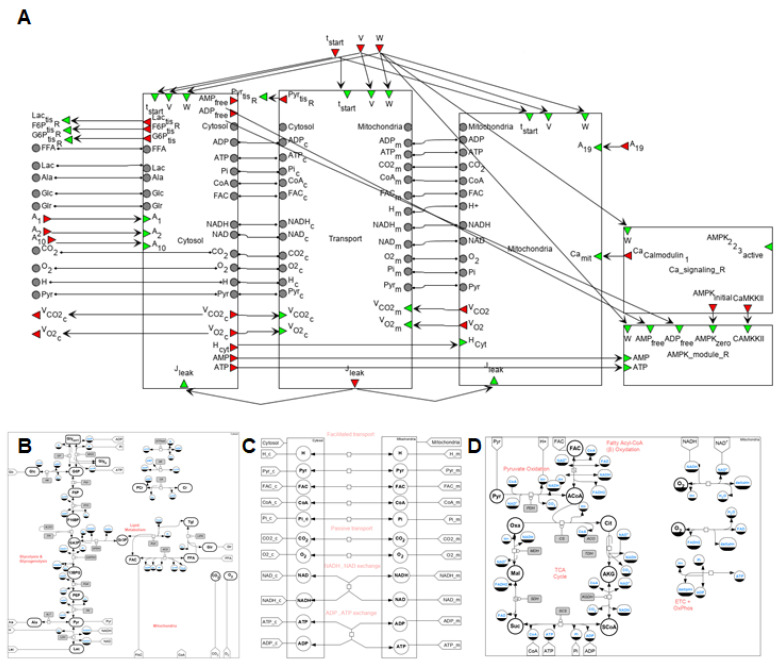
A general SBGN diagram of the modular model describing metabolism in human muscle fibers (**A**) taking into account metabolic processes in the cytoplasm (**B**) and the mitochondrion (**D**), and transport reactions between two compartments (**C**) as well as modules of the Ca^2+^-dependent signaling pathway and AMPK activity considering different isoforms. Designations of input/output/contact ports are described above in the main text. The vertices of the bipartite graph 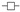 in the Cytosol/Mitochondrion and Transport submodules correspond to the respective biochemical or transport reaction depending on the submodule. Each fiber type module also comprises submodules of Ca^2+^- and AMPK-dependent signaling pathways. All details for each submodule and zoomed-in subfigures are in the Supplementary Material.

The diagram of the modular model describing the metabolism of human skeletal muscle is presented in Figure 6. The cytosol includes metabolic reactions of the glycolysis, glycogenolysis, and lipid metabolism, while the tricarboxylic acid (TCA) cycle, ß-oxidation, and oxidative phosphorylation reactions occur in the mitochondria. The intermediate compartment between those is a transport module that contains passive and facilitated transport reactions for model intracellular species. Kinetic laws presenting metabolic and transport flux expressions exactly match the initial model developed by Li and coauthors [32].

According to the model, a dynamic mass balance of metabolites (*i*) is based on the structural and functional organization and is expressed by equations:

$$\begin{array}{c} V_{cyt,type}\frac{dC_{cyt,\;type,i}}{dt}=R_{cyt,type,i}+T_{bl\langle -\rangle cyt,\;type,i}^{k}-T_{cyt\langle -\rangle mit,\;type,i}^{k}\\ \mathrm{in}\;\mathrm{the}\;\mathrm{cytosol}\;\mathrm{and}: \end{array}$$

$$\begin{array}{c} V_{mit,type}\frac{dC_{mit,\;type,i}}{dt}=R_{mit,type,i}+T_{cyt\langle -\rangle mit,\;type,i}^{k}\\ \mathrm{in}\;\mathrm{the}\;\mathrm{mictochondria}. \end{array}$$

where $V_{cyt}$, $V_{mit}$ indicate the volume of the corresponding module or compartment in kg wet weight (kg w.w.), type∈(type I fiber,type II fiber). $V_{cyt,R}=0.88\times V_{R}$ and $V_{cyt,W}=0.92\times V_{W}$ are volumes of the cytosol for type I and II fibers, respectively, while $V_{mit,R}=0.12\times V_{R}$ and $V_{mit,W}=0.08\times V_{W}$ are the volumes of mitochondria for type I and II fibers, respectively, where $V_{R}=V_{w}=0.5\times V_{tis}=2$ kg w.w., $V_{R}$—the type I fiber volume, $V_{W}$—the type II fiber volume, and $V_{tis}$—the volume of muscle cells in the tissue. $C_{cyt,\;type,i}$, $C_{mit,\;type,i}$ is the concentration of metabolite *i* in a certain compartment of the corresponding fiber type (mmol/kg w.w.); $R_{x,type,i}$, x∈{cyt, mit} is the net metabolic reaction rate in a certain compartment of the corresponding fiber type (mmol/min/kg w.w.); $T_{cyt\langle -\rangle mit,\;type,i}^{k}$, $T_{bl\langle -\rangle cyt,\;type,i}^{k}$ are the respective transport fluxes between the cytosol and mitochondria compartments and cytosol and blood compartments (mmol/kg w.w.), where superscript *k* indicates *f* (facilitated) or *p* (passive) transports.

In order to describe a dynamic mass balance of metabolites (*i*) in the blood compartment, the next equation is used:

$$V_{bl}\frac{dC_{bl,i}}{dt}=Q\times \left(C_{art,i}-C_{bl,i}\right)-T_{bl\langle -\rangle cyt,R,\;i}^{f\;or\;p}\times V_{R}-T_{bl\langle -\rangle cyt,W,\;i}^{f\;or\;p}\times V_{W}$$

where $V_{bl}$ is the total effective volume of the capillary blood and interstitial fluid compartments. $V_{bl}=0.2\times V_{mus}$, $V_{tis}=0.8\times V_{mus}$, where $V_{mus}=V_{tis}+V_{bl}$—the skeletal muscle volume (5 kg w.w.); $C_{art,i}C_{bl,i}$ is the concentration of metabolite *i* in the respective arterial and capillary blood compartments (mmol/kg w.w.).

It is worth noting that such modules as capillary blood and interstitial fluid are assumed to be in equilibrium with each other, which allows us to consider species concentrations in these compartments as equal and combine them into the blood compartment. The comprehensive description including visual representation, corresponding Antimony code, and mathematical equations for reaction rates and the dynamic mass balance in each module of the integrated model as well as values and units of the used kinetic parameters is presented in the Supplementary material.

In comparison with the original model of Li and coauthors [32], we introduced a following changes:

1. Values of activation coefficients associated with ATPase [35,36,37,38] and pyruvate dehydrogenase reaction fluxes for type I and type II fibers [39,40,41] as well as the time constant of the ATPase flux rate coefficient in response to exercise were modified (See Data availability and Supplementary material) according to recently published data and estimations [42,43].
2. Despite overall net glycogen breakdowns during muscle contraction, exercise increases the activity of glycogen synthase (GS) [44,45,46,47] and ATP consumption related with the reaction. Therefore, GS reaction fluxes were modified according to [44,46,48]. The rates of muscle glycogen synthesis during exercise assumed to be equal in type I and type II fibres were estimated from average post-exercise glycogen synthesis data [49].
3. To consider the allosteric regulation of AMPK activity (in corresponding modules), concentrations of free ADP and AMP in the cytosol were calculated using intracellular Cr, PCr, ATP, and H^+^ concentrations as well as the equilibrium constants for creatine phosphokinase and adenylate kinases in each fiber type as described previously [50,51,52].

#### 2.2.5. Signaling Level

**Figure 7:**
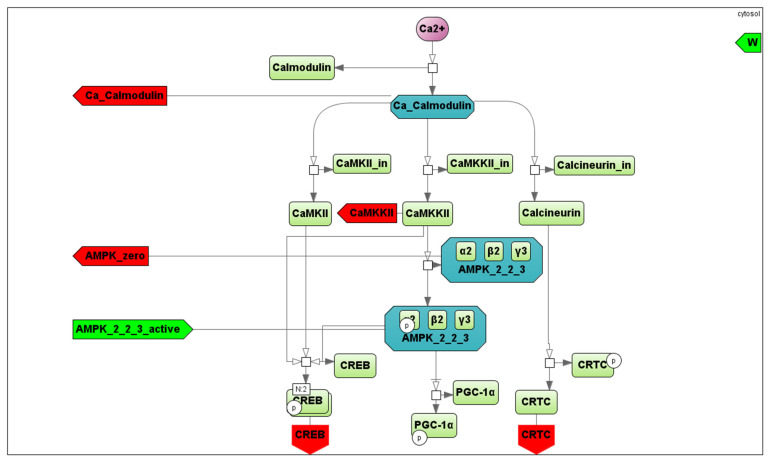
An SBGN diagram of the Ca^2+^- (purple circle) and AMPK-dependent signaling pathways activated by contractile activity (aerobic exercise). A vertex of the bipartite graph 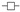 corresponds to a reaction in the signaling cascade, where green rectangles designate corresponding proteins, while dark green octagons represent protein complexes. All abbreviations and aliases of proteins correspond to the main text description. Red arrows (Ca_Calmodulin, CAMKKII, AMPK_zero, CREB and CRTC) correspond to output ports, while the green arrow indicates the input port (AMPK_2_2_3_active).

The mean concentration of Ca^2+^ ions in the myoplasm increases in proportion to the intensity of exercise. Ca^2+^ binds to calmodulin, thereby activating CaMKs and phosphatase calcineurin [33]. CaMKII is the most abundant isoform in human skeletal muscle, whereas CaMKI and CaMKIV are not expressed at detectable levels [53]. An increase in CaMKII activity results in CREB1 Ser133 phosphorylation (and likely some other CREB-like factors), leading to the activation of the transcription factor [54,55]. Calcineurin can dephosphorylate (and activate) CRTCs at Ser171 (CREB-regulated transcription coactivators), playing a key role in regulating the transcriptional activity of CREB1 [56]. Another target of calmodulin is Ca^2+^/calmodulin-dependent protein kinase kinase 2 (CAMKK2) that phosphorylates AMPK Thr172, thereby activating the kinase [57]. In turn, activated AMPK can phosphorylate CREB1 Ser133 [58]. Collectively, these findings drove us to include in our model the Ca^2+^-dependent regulation of calmodulin, CREB1 (via CaMKII), CRTC (via calcineurin), and AMPK (via CaMKK2) (Figure 7). The amount of these proteins in human skeletal muscle was estimated using published proteomics and transcriptomics data [12,59] (see Supplementary data in [60]).

There are three different heterotrimeric complexes of AMPK in the human skeletal muscles: α2β2γ1, α2β2γ3, and α1β2γ1 [61]. Distinct kinetic properties (an intrinsic enzyme activity, binding affinities of AMP, ADP, and ATP to the specific isoform, sensitivity to de- and phosphorylation of AMPK heterotrimers) [62,63] and their subcellular localization [64] cause a differential regulation of the AMPK heterotrimers in vivo. The α2β2γ3 complex is phosphorylated and activated during moderate- to high-intensity aerobic exercise, while the activity associated with the other two AMPK heterotrimers is almost unchanged [65]. However, the baseline activity of the α2β2γ3 complex is significantly lower than others. The general AMPK activity at baseline and during/after exercise is a sum of isoform activities; hence, we considered all isoforms separately (in the corresponding module) to quantitatively fit experimental data obtained at baseline and after an exercise [65,66]. AMPK is regulated in various ways: an up-stream kinase LKB1 can phosphorylate AMPK at Thr172 [67,68]. On the other hand, an exercise-induced decrease in intramuscular ATP increases its activity, while an increase in AMP activates it [69,70]. Hence, in our model, AMPK is regulated via AMP, ATP, and LKB1, as well as CaMKK2 (as mentioned above) (Figure 7).

It was demonstrated that the localizations of AMPK and CaMKII kinases have a pronounced effect on their activities [34,53,71,72,73], implying the necessity to consider the impact in the model. However, an extended analysis of the published data on this issue demonstrates some contradictions in the data and the lack of quantitative data on this issue. For instance, the vast majority of CaMKII (~80%) expressed in human skeletal muscle is localized to the soluble cytosolic fraction. However, most of the major estimations and measurements on the functional properties and substrates have been obtained for membrane-associated CaMKII [53]. Moreover, the mobile fraction of the kinases or their substrates has a limited diffusion rate in the tightly packed myocyte structure and is dependent on the molecular weight that can affect the kinetics of their interaction. Such diffusion rate data have not been found.

#### 2.2.6. Gene Expression Level

An aerobic exercise induces the expression of several hundreds of genes regulating many cell functions: energy metabolism, transport of various substances, angiogenesis, mitochondrial biogenesis, etc. Regulation of the transcriptomic response to acute exercise includes dozens of transcription regulators [12] and seems to be extremely complex. Therefore, to consider the response at a gene expression level, we selected exercise-induced genes based on the next criterion comprising two points: (1) a functional role of this gene in the regulation of skeletal muscle metabolism is known; (2) its expression in human skeletal muscle markedly changes in response to an exercise and has one of the expression patterns—early or delayed response since gene expression in early and late stages of the recovery after the termination of the exercise can be regulated in different ways [13]. According to the criterion, the *PPARGC1A* gene, known as the major regulator of exercise-induced phenotypic adaptation and substrate utilization [74], was chosen as the gene with delayed response, while *NR4A2* and *NR4A3* genes were chosen as early response genes [75]. NR4A nuclear receptors induce DNA demethylation in skeletal muscle [76], regulate genes involved in glycogenolysis, fatty acid oxidation, and pyruvate use and apparently play a role in the regulation of the skeletal muscle fiber phenotype [77,78]. Significantly, all members of the NR4A nuclear receptor subfamily (*NR4A1, NR4A2, NR4A3*) are the three most highly induced genes in response to acute endurance exercise [79,80]. We selected both genes from one family since they have different temporal patterns of mRNA expression that are likely associated with different methylation profiles of their promoters [81,82].

Expression of *NR4A2* and *NR4A3* mRNA rapidly increases during the first hour after an aerobic exercise (early response genes) [12] due to activation of Ca^2+^\calcineurin-dependent signaling [75]. We included in our model the Ca^2+^-dependent regulation (Ca^2+^\calcineurin-CaMKII-CREB1) of *NR4As* genes using data of contractile activity-specific mRNA responses of these genes [12]. Expression of *PPARGC1A* mRNA rises 3 to 4 h after an exercise (gene with delayed response) [12]. The transcription regulation of *PPARGC1A* via the canonical (proximal) and inducible (distal) promoters is very complicated and includes Ca^2+^- and AMPK-dependent signaling, as well as CREB1 and its co-activator CRTC [10,83]. The phosphorylation level of many signaling kinases drops to basal levels within the first hour after an aerobic exercise. Moreover, in a genome-wide study on various human tissues, it was shown that the phosphorylation level of CREB Ser133 does not always correlate with its transcriptional activity [81]. Therefore, we suggested that the expression of genes with delayed response (including *PPARGC1A*) is regulated by increasing the expression of one of the early response genes encoding transcription factors leading to a rapid increase in the corresponding protein [60]. A detailed description of our results on the identification of transcription factors as potential candidates for the role of X factor is presented below in the section Results and Discussion. Analysis of contractile activity-specific transcriptomic data [12] showed that a rapid increase in the expression of genes encoding various TFs is observed already in the first hour after an exercise. It turned out that the binding motifs of some TFs (CREB-like proteins, as well as proteins of the AP-1 family: FOS and JUN) are located and intersect with each other both in the alternative and in the canonical promoters of the *PPARGC1A* gene [60], i.e., these TFs can act as potential regulators of this gene. This is consistent with the fact that these TFs can bind to DNA and regulate the expression of target genes as homo- and heterodimers [84,85]. Based on these considerations, we included in the model the regulation of gene expression of early (*NR4A2*, *NR4A3*) and delayed (*PPARGC1A*) genes: early response genes are regulated via the activation of existing TFs (e.g., CREB1) and their co-activators (e.g., CRTC), while delayed response genes are regulated via an increase in the expression of early response genes encoding transcription factors (transcription factor X in our model, Supplementary Material, Module “Gene expression regulation”).

## 3. Results and Discussion

### 3.1. Model Validation

#### 3.1.1. Simulation of Metabolic Changes Induced by Incremental and Interval Exercises

**Figure 8:**
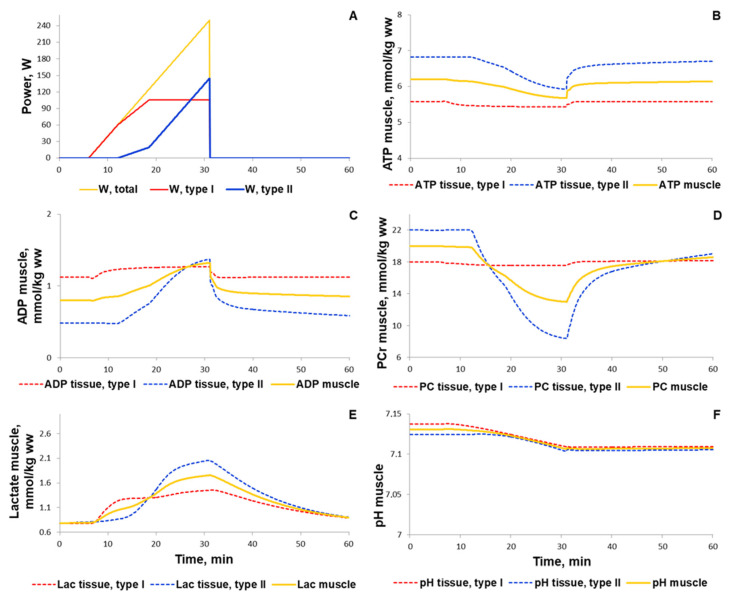
Simulation results for the incremental ramp exercise until exhaustion. (**A**) Exercise power and fiber recruitment pattern: total power (orange), power generated by type I (red) and II (blue) fibers; (**B**–**D**) ATP, ADP, and PCr concentrations in type I (red, dotted) and II (blue, dotted) fibers and in the muscle tissue (orange, solid); (**E**,**F**) Lactate concentrations and pH changes in type I (red, dotted) and II (blue, dotted) fibers and in the muscle tissue (orange, solid).

To validate the metabolic part of the model, we investigated the dynamic behaviour of the system in response to diverse acute aerobic exercises and compared them with published experimental data. It is worth noting that qualitative validation of the model was conducted on the basis of the comparison of the simulation and experimental data for three indicators: time period to achieve the maximal level of the species concentrations (e.g., PCr, ATP, glycogen) at the corresponding value of the exercise intensity and time to reach the steady-state value in recovery as well as the multiplicity of concentration changes (fold changes). We used the last indicator due to quantitative differences in measured concentrations for the same species by different experimental approaches (e.g., biochemical and ^31^P MRS measurements). Initially, we quantitatively estimated the biochemical responses of the key metabolic variables (ATP, ADP, PCr, lactate concentrations, and pH in muscle fibers type I and II) in the incremental ramp exercise to exhaustion, which is a commonly used approach to evaluate aerobic performance. Increasing the power during the ramp exercise affects various physiological variables such as the number/volume of recruited muscle fibre type I and II, blood flow as well as the transport and metabolic fluxes in both fibre types (Figure 8 and see data availability). In our simulation, muscle fibres type I start to be recruited after the beginning of exercise, while fibre type II if only recruited at a power higher than 24% of VO2_max_ (6 min after the ramp exercise onset, Figure 8A). Recruiting all muscle fibres during the test leads to exhaustion and termination of the exercise [35,86,87]; the peak power at exhaustion in our simulation was 250 W, which corresponds to the value for an untrained male performing the ramp exercise until exhaustion using a cycling ergometer. The model simulations correspond reasonably well to experimental measurements [88,89,90] obtained in studies with the incremental exercise (Supplementary Figure S1). It is worth noting that the current version of the model does not take into account the effect of muscle fatigue during the incremental ramp exercise observed in exercised muscle in vivo (see below). This fact may partially explain the lack of exponential changes in muscle lactate concentration and pH during the last part of the incremental exercise.

**Figure 9:**
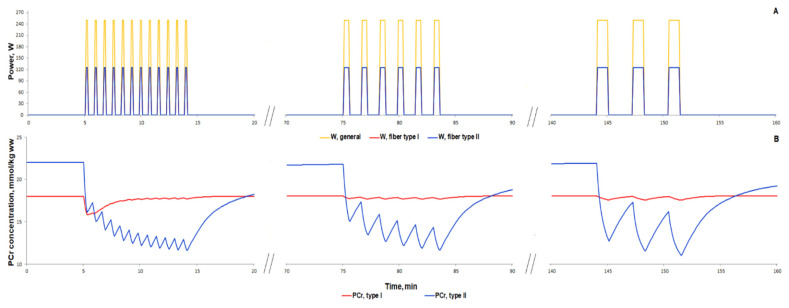
Simulation results for high-intensity intermittent exercise bouts with different ratios of work:recovery (initial—16:32 s; intermediate—32:64 s; final—64:128 s) [91]. (**A**) Exercise power and fiber recruitment pattern: total power (W_peak_ = 250) (orange), power generated by type I (red, W_peak_ = 125) and II (blue, W_peak_ = 125) fibers; (**B**) PCr concentration in type I (red) and type II fibers (blue).

**Figure 10:**
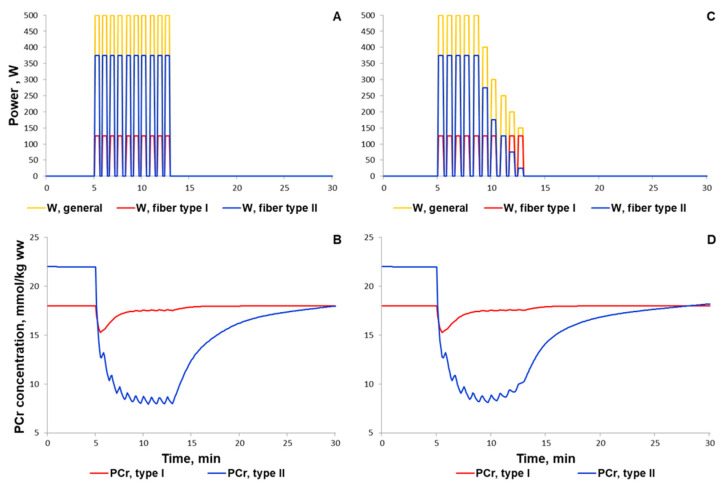

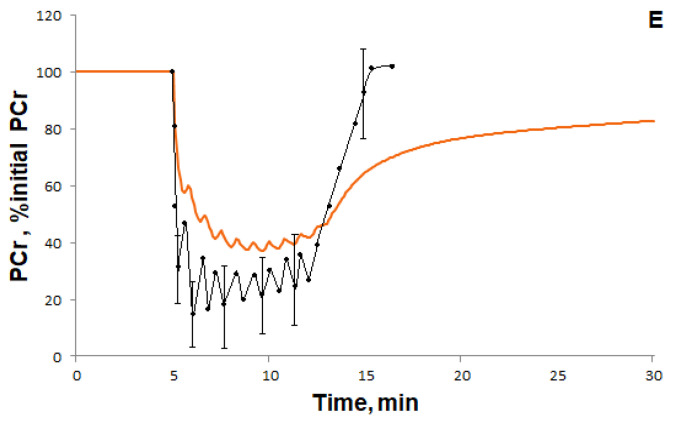
Simulation results for high-intensity intermittent exercise (each bout of 30 s exercise separated by 20 s recovery; [92]). (**A**,**C**) Exercise power and fiber recruitment pattern: total power (A: W_peak_ = 500; C: W_peak_ = 500), W (orange), power generated by type I (red, A–C: W_peak_ = 125) and II (blue, A: W_peak_ = 375; C: W_peak_ = 375 and successive power decline) fibers; (**B**,**D**) PCr concentration in type I (red) and type II fibers (blue); (**E**) Changes in PCr concentration (% initial): the orange line is the simulation result for PCr in the muscle tissue, while black dots with the corresponding line are the experimental data from [92] (mean ± SD for some dots).

For additional validation of the metabolic part of the model, we simulated responses to various interval exercises (Figure 9 and Figure 10). Figure 9 shows that the model qualitatively reproduces the dynamics of PCr concentration during the interval exercises with different patterns (duration of an exercise bout 16 s to 64 s and recovery period 32 s to 128 s) and with peak power comparable with maximal aerobic power obtained in the incremental ramp test (250 W) [91].

High-intensity interval exercise has been shown to rapidly decrease the PCr level followed by slow recovery of the PCr concentration during the last part of the exercise [92]. There are no data on the exercise power in the study; hence, we used the constant value (500 W) for each bout (Figure 10A). The power was markedly higher than the peak power in the incremental cycling test because the duration of each exercise bout is short; the energy supply of such short exercise bouts is related mainly to PCr reactions as well as glycolysis. Our model precisely simulated the rapid decline in PCr, but showed no slow recovery of the PCr during the last part of the high-intensity interval exercise (Figure 10B). We suggested that this discrepancy may be related to the lack of the fatigue-induced decline in exercise power. We tried to roughly simulate the fatigue-induced decline in exercise power by the decline in power generated by muscle fibers type II (Figure 10C). As a result, the model much better reproduced the experimental dynamics of PCr than simulations with constant maximal power in each bout (Figure 10D,E, Supplementary Figure S2). However, the PCr dynamics during the recovery process indicated that the model still requires further modifications and numerical study. We assume that the potential point for the update is related to the pH changes during the recovery.

#### 3.1.2. Simulation of Signaling and Gene Expression Changes Induced by Low- and Moderate Intensity Continuous Exercises

**Figure 11:**
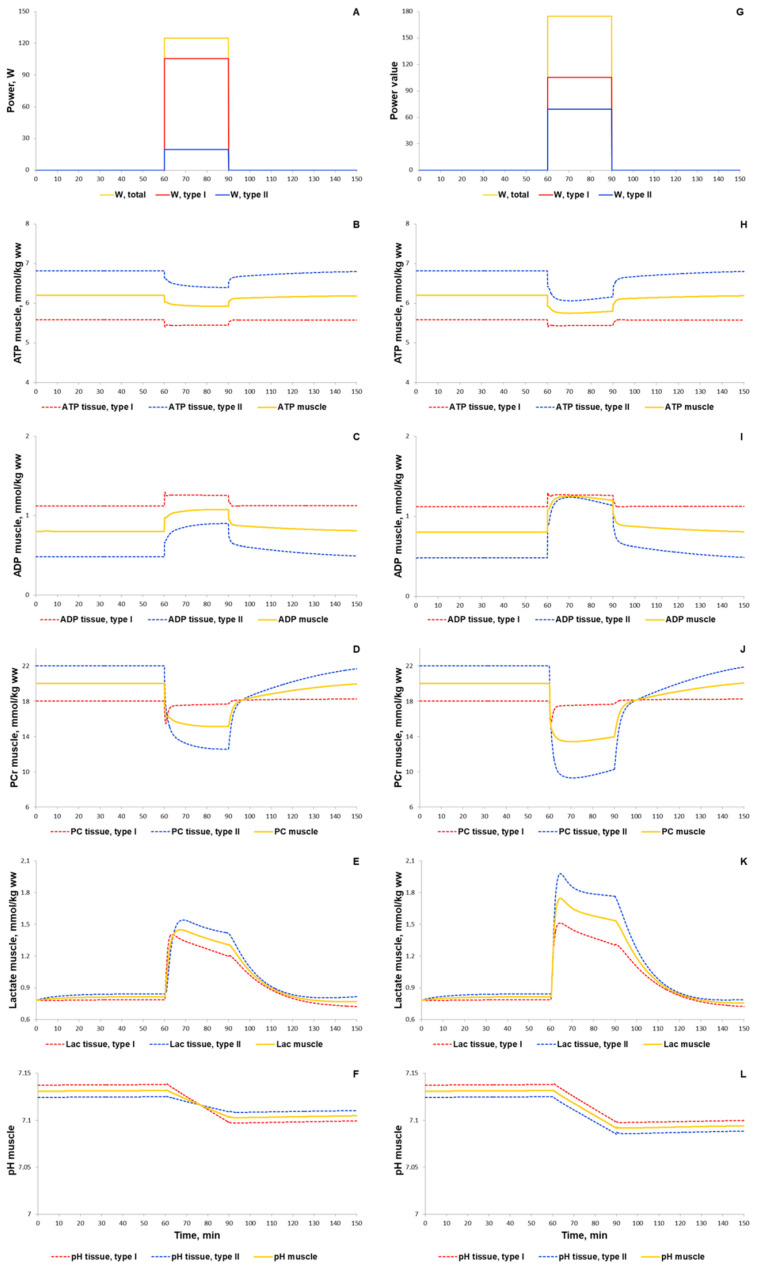
Simulation results for low (50% VO2_max_, **A**–**F**) and moderate intensity (70% VO2_max_, **G**–**L**) continuous exercises (30 min). (**A**,**G**) Exercise power and fiber recruitment pattern: total power (orange), power generated by type I (red) and II (blue) fibers; (**B**,**H**) ATP, ADP, PCr, lactate concentrations, and pH changes in type I (red, dotted) and II (blue, dotted) fibers and in the muscle tissue (orange, solid) during low (**B**–**F**) and moderate (**H**–**L**) intensity exercise.

At the next step of the model validation, we predicted the responses of biochemical variables, signaling molecules (AMPK and Ca^2+^-dependent proteins), transcription factor (CREB1), as well as expression of genes with early and delayed responses (*NR4A3*, *NR4A2*, *PPARGC1A*) to low (50% VO2_max_) and moderate intensity (70% VO2_max_) continuous aerobic exercises (Figure 11). Moderate intensity exercise recruits more muscle fibers type II than low intensity exercise, thereby additionally modulating the exercise-induced metabolic fluxes and molecular response. A comparison of our simulations with experimental data [43,90,93,94,95] showed that the model well reproduces the metabolic changes in various fiber types and in the whole muscle induced by exercises with various intensity (Supplementary Figure S1).

**Figure 12:**
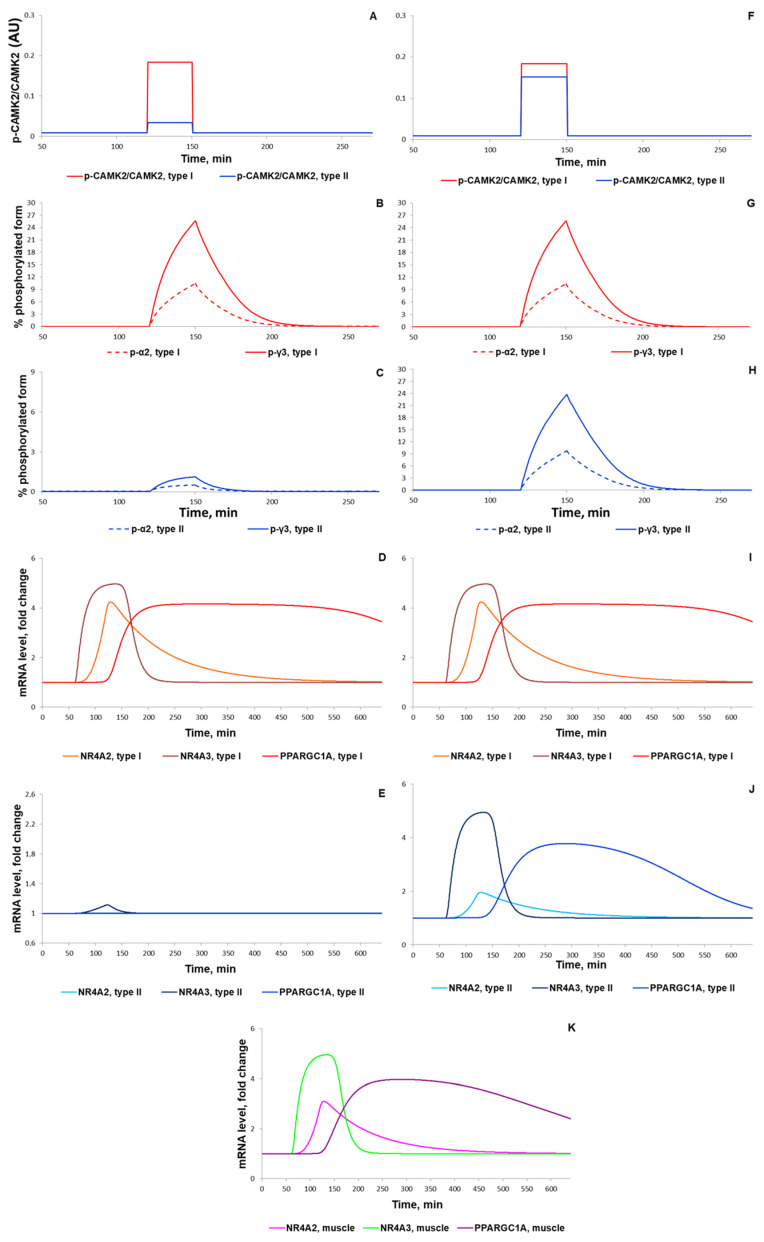
Simulation results for low and moderate intensity (50% (**A**–**E**) and 70% (**F**–**K**) VO2_max_, respectively) continuous exercises (30 min for signaling and 60 min for gene expression) with intermediate X factor regulating the expression of the *PPARGC1A* gene. (**A**,**F**) Ratio of CAMKII phosphorylated protein in type I (red) and type II (blue) fibers (corresponds to [53]); (**B**,**C**,**G**,**H**) Percentage of all α2 phosphorylated proteins (dashed) and of the phosphorylated γ3 heterotrimers (solid) in type I fibers and type II fibers, respectively (corresponds to [65,96]); (**D**,**I**) Expression (in fold changes) of *NR4A3* (thunderbird solid), *NR4A2* (orange solid), *PPARGC1A* (red solid) in type I fibers and (**E**,**J**) in type II fibers, where *NR4A3*—sapphire solid, *NR4A2*—azure solid, *PPARGC1A*—blue solid; (**K**) Expression (in fold changes) of *NR4A3* (green solid), *NR4A2* (magenta solid), and *PPARGC1A* (purple solid) in the muscle tissue during moderate intensity exercise.

According to the literature data on the human vastus lateralis muscle [53,65,96], our simulation showed an intensity-dependent increase in the phosphorylation of CAMKII and AMPK α2 and γ3 (Figure 12A–C,F–H). Importantly, the phosphorylation (as a marker of activity) of AMPK α2 and γ3 consisted of 10% and 30% of the AMPK isoforms containing α2 and γ3, respectively (Figure 12B–C,G–H), which is in line with the experimental data [65]. In contrast to experimental data at a signaling level, we found transcriptomics data concerning intensity-dependent gene expression for 1 h exercise only [10,12]. In our model, exercise-induced activation of CAMKII and AMPK induced CREB- and CRTC-related expression of early response genes that is in line with the experimental data [12] on exercise-induced expression of early response genes (for example, of *NR4A2*, *NR4A3*) in the human vastus lateralis muscle (Figure 12K).

Numerical analysis of the model demonstrated the necessity of considering additional transcription factors showing activity 1 to 2 h after exercise for the simulation of genes with a delayed response to exercise (for example, *PPARGC1A*; [60]). Introducing in the model transcription factor X that is up-regulated immediately after exercise in a CREB- and CRTC-dependent manner allowed us to reproduce the expression of the *PPARGC1A* gene (Figure 12K). Our bioinformatics analysis [60] of the transcriptomics data [12], in turn, allowed us to suggest that proteins from the AP-1 family (e.g., FOS and JUN) forming heterodimer complexes with CREB-like transcription factors served as these intermediate regulators (factor X; see details in Supplementary Figure S3 and Table S1). Moreover, an analysis of the transcriptomic [12,80] and ChIP-seq data from the GTRD database [97] revealed that the expression of *PPARGC1A* via the alternative promoter may be regulated by EGR1 and MYC. Both *EGR1* and *MYC* markedly induced expression 30 to 60 min after an aerobic exercise and had binding motifs in the alternative promoter. Our prediction is supported by experimental data showing that *EGR1* expression leads to an increase in *PPARGC1A* expression in human aortic smooth muscle cells [98,99], while the *EGR1* expression promptly and dramatically increased after the stretching of skeletal muscle cells, leading to an increase in the concentration of the EGR1 protein in 3–4 h [100]. On the one hand, MYC positively regulates the expression of all active genes through transcriptional amplification [101,102,103] and chromatin modifications [104,105]. However, an enhancement of its expression negatively impacts *PPARGC1A* expression [106,107], in particular, in cardiomyocytes [108] and other types of cells where MYC acts as a repressor [109].

### 3.2. The Integrated Modular Model Comprises Three Hierarchical Levels (Metabolic, Signaling, and Gene Expression)

We previously developed a multi-compartmental mathematical model describing the dynamics of intracellular species concentrations and fluxes in human muscle at rest and intracellular metabolic rearrangements in exercising skeletal muscles during aerobic exercise on a cycle ergometer [16]. As an initial model for this study, we used a complex model of energy metabolism in the human skeletal muscle developed by Li and coauthors and considered two types of muscle fibers [32]. We proposed a modular representation of the complex model using the BioUML platform [17]. The modular representation provides the possibility of rapid expansion and modification of the model compartments to account for the complex organization of muscle cells and the limitations of the rate of diffusion of metabolites between intracellular compartments. This feature allowed us to integrate modules of signaling pathways modulating downstream regulatory processes of early response genes and genes with delayed response during exercise and recovery. The validation of the modular model based on a higher number of published experimental data [43,89,90,93,94] (see Supplementary Figure S1) than were used in the original metabolic model [32] showed the validity of the modular modeling approach implemented in BioUML. Furthermore, the integrated modular model provides an absolutely novel in silico platform to predict molecular responses of human skeletal muscle cells to diverse modes of exercise on three hierarchical levels (metabolic, signaling, and gene expression), experimental precise measurements of which are currently methodologically limited or even remain elusive.

In the current state, the model is suitable for testing the plausibility of some physiological hypotheses. For example, the existence of intermediate X factor regulating the expression of the *PPARGC1A* gene as the example of a delayed response gene in human skeletal muscle has been numerically investigated using different versions of the model: considering direct regulation via the CREB-like factor or taking into account the X factor regulatory role as an intermediate activator of *PPARGC1A* expression.

### 3.3. Model Constraints and Further Ways for Development

Despite the complexity of the developed modular model, the current version does not consider the influence of many system factors such as hormonal regulation [56,110], the influence of processes in the central nervous system [111,112], feeding mode [113,114], and exercise-induced temperature drift in skeletal muscle [115,116], which hampers the precise quantitative reproduction of abrupt changes at different physiological levels during initial stages of physical exercise. It can be overcome by means of significant modifications on the muscle fiber recruitment model in order to simulate the transient process due to exercise. Some other constraints are described in detail below.

GS activity is regulated through multiple mechanisms, including feedbacks mediated by glycogen, blood glucose concentration, rate of glucose uptake, insulin, epinephrine, and the GS phosphorylation state [46,48,117,118]. However, in the current model, GS activity depends on the glycogen content only. In our model, post-exercise glycogen synthesis is lower than that estimated in the majority of studies [49,119,120] because many factors are omitted, such as feeding and associated rises in blood glucose concentration, rate of glucose uptake, sensitivity to and changes in insulin, etc. At the same time, in our model, glycogen synthesis is higher than that observed during exercise recovery in a fasted state [121].

Additionally, our model does not take into account the effect of muscle fatigue related to the decline in power generated by type II muscle fibres and recruitment of new type II fibres as well as the depletion of muscle glycogen and other substrates. This may play an important role in the simulation of moderate and high intensive and/or long-lasting exercise. Moreover, the focus of this study is related to the recruitment of vastus lateralis muscle fibers and their activation at metabolic, signaling, and gene expression regulation levels as a response to the exercise performed according to a cycle-ergometer or knee-extensor exercises only. These limitations provide a direction for model improvements and should be considered in further works.

Furthermore, the modular nature of the presented model allows the introduction of multiple positive and negative feedbacks between different considered levels: for instance, the impact of kinases altering the activity of enzymes that catalyze reactions of the glycolysis, TCA cycle, and fatty acid oxidation in skeletal muscle [122,123], Ca^2+^-dependent enhancement of glycolytic enzyme activity and mitochondrial respiration [33], and PGC1α-dependent regulation of the expression of genes encoding glycolysis and malate–aspartate shuttle enzymes [124].

Our model provides a proof of concept of how dynamic changes at the metabolic level can be linked to gene expression regulation via signaling transduction pathways in skeletal muscles during physical exercises. The modular approach used in the study has demonstrated a methodological basis for qualitative and quantitative development of the complex model including different hierarchical levels of the system organization. The analysis completed during this study allows us to refine the roadmap for further model improvements, linking this in silico version to in vivo skeletal muscle. The roadmap includes an improvement of the motor unit recruitment model, considering the impact of the muscle fatigue on power decrease, and extension of the model by new modules representing system factors, e.g., hormonal regulation and the central nervous system taking into account multiple relationships and feedbacks between different modules of the integrated model.

## 4. Conclusions

We developed, for the first time, an integrated model of human skeletal muscle incorporating metabolic, signaling, and gene expression modules. The model enables us to simulate the most important exercise-related signaling (Ca^2+^ and AMPK-related signaling) and RNA expression of early response genes (as a result of the activation of transcription factors existing in the cell), as well as the expression of delayed response genes (as a result of the expression of intermediate transcription factors induced immediately after an exercise). The molecular response of skeletal muscle to contractile activity is related to the high number of signaling molecules and genes. The modular nature of the model enables us to add new variables and modules, thereby increasing both the complexity and quality of the model.

## Figures and Tables

**Table 1:** Glyphs for new entities for the SBGN Process Diagram.

Element Name	Glyph	Description
Equation	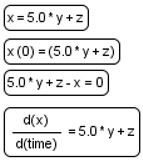	Mathematical equations of the model: assignments; initial assignments; algebraic equations; differential equations.
Event	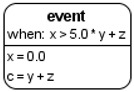	Events describing instant changes made to model variables when a specified condition is satisfied, i.e., when trigger expression changes its value from “false” to “true”.
Function	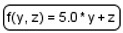	Function receives argument values, calculates, and returns result of the given expression.
Constraint	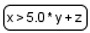	Constraint is checked during the simulation. If it is violated, simulation is either stopped or an error message is shown depending on solver settings.
Tabular data	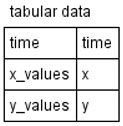	Tabular element is used to calculate model variables based on tabular data. In this example, the time column is used for the time variable; x_value and y_value columns are used for x and y, respectively. The tabular element is either translated to a spline approximating tabular data or a piecewise constant function.
Interface ports	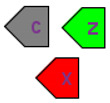	Although SBGN notation already has tag elements that denote the module interface (ports in SBML terminology), in our diagrams we have three different types of ports: contact ports (gray), input ports (green), and output ports (red).

**Table 2:** Glyphs and arcs for modular diagrams.

Element Name	Glyph	Description
Module	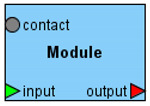	Module encapsulates the mathematical model (submodel) of a particular subsystem forming the hierarchic structure of the model.
Port	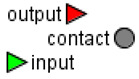	Ports represent the interface of the module through which it can be connected with other modules or with the hierarchical model itself. The color of the port defines its type: output (red), input (green), contact (gray).
Connection	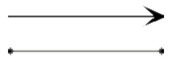	Connections can be established between ports to aggregate modules with each other. Directed connections are established between output and input ports, with undirected connections between contact ports.
Bus	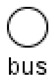	Buses are auxiliary elements that can be used as intermediate points in connections. They decrease the number of intersections between connections and make the structure of the model more clear.

## Data Availability

https://gitlab.sirius-web.org/virtual-patient/muscle-metabolism, accessed on 8 June 2021. Git project on the Sirius platform contains all source codes for the developed model and its modules. README.md file on the start page describes how users can visualize, edit and simulate the model using the BioUML and Jupyter Notebook.

## Supplementary Materials

The following are available online at https://www.mdpi.com/article/10.3390/ijms221910353/s1.

## References

[1] Pedersen B.K., Febbraio M.A. (2012). Muscles, exercise and obesity: Skeletal muscle as a secretory organ. Nat. Rev. Endocrinol..

[2] Hawley J.A., Hargreaves M., Joyner M.J., Zierath J.R. (2014). Integrative biology of exercise. Cell.

[3] Koulmann N., Bigard A.X. (2006). Interaction between signalling pathways involved in skeletal muscle responses to endurance exercise. Pflügers Arch..

[4] Richter E.A., Ruderman N.B. (2009). AMPK and the biochemistry of exercise: Implications for human health and disease. Biochem. J..

[5] Hoffman N.J., Parker B.L., Chaudhuri R., Fisher-Wellman K.H., Kleinert M., Humphrey S.J., Yang P., Holliday M., Trefely S., Fazakerley D.J. (2015). Global phosphoproteomic analysis of human skeletal muscle reveals a network of exercise-regulated kinases and AMPK substrates. Cell Metab..

[6] Needham E.J., Humphrey S.J., Cooke K.C., Fazakerley D.J., Duan X., Parker B.L., James D.E. (2019). Phosphoproteomics of Acute Cell Stressors Targeting Exercise Signaling Networks Reveal Drug Interactions Regulating Protein Secretion. Cell Rep..

[7] Nelson M.E., Parker B.L., Burchfield J.G., Hoffman N.J., Needham E.J., Cooke K.C., Naim T., Sylow L., Ling N.X., Francis D. (2019). Phosphoproteomics reveals conserved exercise-stimulated signaling and AMPK regulation of store-operated calcium entry. EMBO J..

[8] Vissing K., Schjerling P. (2014). Simplified data access on human skeletal muscle transcriptome responses to differentiated exercise. Sci. Data.

[9] Dickinson J.M., D’Lugos A.C., Naymik M.A., Siniard A.L., Wolfe A.J., Curtis D.R., Huentelman M.J., Carroll C.C. (2018). Transcriptome response of human skeletal muscle to divergent exercise stimuli. J. Appl. Physiol..

[10] Popov D.V., Makhnovskii P.A., Kurochkina N.S., Lysenko E.A., Vepkhvadze T.F., Vinogradova O.L. (2018). Intensity-dependent gene expression after aerobic exercise in endurance-trained skeletal muscle. Biol. Sport.

[11] Neubauer O., Sabapathy S., Ashton K.J., Desbrow B., Peake J.M., Lazarus R., Wessner B., Cameron-Smith D., Wagner K.H., Haseler L.J. (2014). Time course-dependent changes in the transcriptome of human skeletal muscle during recovery from endurance exercise: From inflammation to adaptive remodeling. J. Appl. Physiol..

[12] Popov D.V., Makhnovskii P.A., Shagimardanova E.I., Gazizova G.R., Lysenko E.A., Gusev O.A., Vinogradova O.L. (2019). Contractile activity-specific transcriptome response to acute endurance exercise and training in human skeletal muscle. Am. J. Physiol.-Endocrinol. Metab..

[13] Bahrami S., Drabløs F. (2016). Gene regulation in the immediate-early response process. Adv. Biol. Regul..

[14] Li Y., Dash R.K., Kim J., Saidel G.M., Cabrera M.E. (2009). Role of NADH/NAD+ transport activity and glycogen store on skeletal muscle energy metabolism during exercise: In silico studies. Am. J. Physiol.-Cell Physiol..

[15] Akberdin I.R., Kazantsev F.V., Ermak T.V., Timonov V.S., Khlebodarova T.M., Likhoshvai V.A. (2013). In silico cell: Challenges and perspectives. Math. Biol. Bioinform..

[16] Kiselev I.N., Akberdin I.R., Vertyshev A.Y., Popov D.V., Kolpakov F.A. (2019). A modular visual model of energy metabolism in human skeletal muscle. Math. Biol. Bioinform..

[17] Kolpakov F., Akberdin I., Kashapov T., Kiselev I., Kolmykov S., Kondrakhin Y., Kutumova E., Mandrik N., Pintus S., Ryabova A. (2019). BioUML: An integrated environment for systems biology and collaborative analysis of biomedical data. Nucleic Acids Res..

[18] Kutumova E.O., Kiselev I.N., Sharipov R.N., Lavrik I.N., Kolpakov F.A. (2012). A modular model of the apoptosis machinery. Advances in Systems Biology.

[19] Kiselev I.Y.N., Semisalov B.V., Biberdorf E.A., Sharipov R.N.E., Blokhin A.M., Kolpakov F.A.E. (2012). Modular modeling of the human cardiovascular system. Math. Biol. Bioinform..

[20] Porubsky V.L., Goldberg A.P., Rampadarath A.K., Nickerson D.P., Karr J.R., Sauro H.M. (2020). Best practices for making reproducible biochemical models. Cell Syst..

[21] Tiwari K., Kananathan S., Roberts M.G., Meyer J.P., Sharif Shohan M.U., Xavier A., Maire M., Zyoud A., Men J., Ng S. (2021). Reproducibility in systems biology modelling. Mol. Syst. Biol..

[22] Hucka M., Bergmann F.T., Chaouiya C., Dräger A., Hoops S., Keating S.M., König M., Le Novère N., Myers C.J., Olivier B.G. (2019). The Systems Biology Markup Language (SBML): Language specification for level 3 version 2 core. J. Integr. Bioinform..

[23] Smith L.P., Hucka M., Hoops S., Finney A., Ginkel M., Myers C.J., Moraru I., Liebermeister W. (2015). SBML level 3 package: Hierarchical model composition, version 1 release 3. J. Integr. Bioinform..

[24] Le Novere N., Hucka M., Mi H., Moodie S., Schreiber F., Sorokin A., Demir E., Wegner K., Aladjem M.I., Wimalaratne S.M. (2009). The systems biology graphical notation. Nat. Biotechnol..

[25] Smith L.P., Bergmann F.T., Chandran D., Sauro H.M. (2009). Antimony: A modular model definition language. Bioinformatics.

[26] Hartwell L.H., Hopfield J.J., Leibler S., Murray A.W. (1999). From molecular to modular cell biology. Nature.

[27] Alon U. (2003). Biological networks: The tinkerer as an engineer. Science.

[28] Blinov M.L., Ruebenacker O., Moraru I.I. (2008). Complexity and modularity of intracellular networks: A systematic approach for modelling and simulation. IET Syst. Biol..

[29] Neal M.L., Cooling M.T., Smith L.P., Thompson C.T., Sauro H.M., Carlson B.E., Cook D.L., Gennari J.H. (2014). A reappraisal of how to build modular, reusable models of biological systems. PLoS Comput. Biol..

[30] Brown P.N., Byrne G.D., Hindmarsh A.C. (1989). VODE: A variable-coefficient ODE solver. SIAM J. Sci. Stat. Comput..

[31] Bergmann F.T., Adams R., Moodie S., Cooper J., Glont M., Golebiewski M., Hucka M., Laibe C., Miller A.K., Nickerson D.P. (2014). COMBINE archive and OMEX format: One file to share all information to reproduce a modeling project. BMC Bioinform..

[32] Li Y., Lai N., Kirwan J.P., Saidel G.M. (2012). Computational model of cellular metabolic dynamics in skeletal muscle fibers during moderate intensity exercise. Cell. Mol. Bioeng..

[33] Gehlert S., Bloch W., Suhr F. (2015). Ca^2+^-dependent regulations and signaling in skeletal muscle: From electro-mechanical coupling to adaptation. Int. J. Mol. Sci..

[34] Kjøbsted R., Hingst J.R., Fentz J., Foretz M., Sanz M.N., Pehmøller C., Shum M., Marette A., Mounier R., Treebak J.T. (2018). AMPK in skeletal muscle function and metabolism. FASEB J..

[35] Stienen G.J., Kiers J.L., Bottinelli R., Reggiani C. (1996). Myofibrillar ATPase activity in skinned human skeletal muscle fibres: Fibre type and temperature dependence. J. Physiol..

[36] He Z.H., Bottinelli R., Pellegrino M.A., Ferenczi M.A., Reggiani C. (2000). ATP consumption and efficiency of human single muscle fibers with different myosin isoform composition. Biophys. J..

[37] Szentesi P., Zaremba R., Van Mechelen W., Stienen G.J.M. (2001). ATP utilization for calcium uptake and force production in different types of human skeletal muscle fibres. J. Physiol..

[38] Barclay C.J. (2017). Energy demand and supply in human skeletal muscle. J. Muscle Res. Cell Motil..

[39] Parolin M.L., Chesley A., Matsos M.P., Spriet L.L., Jones N.L., Heigenhauser G.J. (1999). Regulation of skeletal muscle glycogen phosphorylase and PDH during maximal intermittent exercise. Am. J. Physiol.-Endocrinol. Metab..

[40] Kiilerich K., Birk J.B., Damsgaard R., Wojtaszewski J.F., Pilegaard H. (2008). Regulation of PDH in human arm and leg muscles at rest and during intense exercise. Am. J. Physiol.-Endocrinol. Metab..

[41] Albers P.H., Pedersen A.J., Birk J.B., Kristensen D.E., Vind B.F., Baba O., Nøhr J., Højlund K., Wojtaszewski J.F. (2015). Human muscle fiber type–specific insulin signaling: Impact of obesity and type 2 diabetes. Diabetes.

[42] Broxterman R.M., Layec G., Hureau T.J., Morgan D.E., Bledsoe A.D., Jessop J.E., Amann M., Richardson R.S. (2017). Bioenergetics and ATP synthesis during exercise: Role of group III/IV muscle afferents. Med. Sci. Sports Exerc..

[43] Bartlett M.F., Fitzgerald L.F., Nagarajan R., Hiroi Y., Kent J.A. (2020). Oxidative ATP synthesis in human quadriceps declines during 4 min of maximal contractions. J. Physiol..

[44] Wojtaszewski J.F., Nielsen P., Kiens B., Richter E.A. (2001). Regulation of glycogen synthase kinase-3 in human skeletal muscle: Effects of food intake and bicycle exercise. Diabetes.

[45] Nielsen J.N., Richter E.A. (2003). Regulation of glycogen synthase in skeletal muscle during exercise. Acta Physiol. Scand..

[46] Jensen J., Lai Y.C. (2009). Regulation of muscle glycogen synthase phosphorylation and kinetic properties by insulin, exercise, adrenaline and role in insulin resistance. Arch. Physiol. Biochem..

[47] Jensen T.E., Richter E.A. (2012). Regulation of glucose and glycogen metabolism during and after exercise. J. Physiol..

[48] Jensen J., Tantiwong P., Stuenæs J.T., Molina-Carrion M., DeFronzo R.A., Sakamoto K., Musi N. (2012). Effect of acute exercise on glycogen synthase in muscle from obese and diabetic subjects. Am. J. Physiol.-Endocrinol. Metab..

[49] Casey A., Short A.H., Hultman E., Greenhaff P.L. (1995). Glycogen resynthesis in human muscle fibre types following exercise-induced glycogen depletion. J. Physiol..

[50] Lawson J.W., Veech R.L. (1979). Effects of pH and free Mg2+ on the Keq of the creatine kinase reaction and other phosphate hydrolyses and phosphate transfer reactions. J. Biol. Chem..

[51] Dudley G.A., Terjung R.L. (1985). Influence of acidosis on AMP deaminase activity in contracting fast-twitch muscle. Am. J. Physiol.-Cell Physiol..

[52] Mannion A.F., Jakeman P.M., Willan P.L. (1993). Determination of human skeletal muscle buffer value by homogenate technique: Methods of measurement. J. Appl. Physiol..

[53] Rose A.J., Kiens B., Richter E.A. (2006). Ca^2+^-calmodulin-dependent protein kinase expression and signalling in skeletal muscle during exercise. J. Physiol..

[54] Johannessen M., Moens U. (2007). Multisite phosphorylation of the cAMP response element-binding protein (CREB) by a diversity of protein kinases. Cell. Signal. Apoptosis Res..

[55] Olesen J., Kiilerich K., Pilegaard H. (2010). PGC-1α-mediated adaptations in skeletal muscle. Pflügers Arch.-Eur. J. Physiol..

[56] Altarejos J.Y., Montminy M. (2011). CREB and the CRTC co-activators: Sensors for hormonal and metabolic signals. Nat. Rev. Mol. Cell Biol..

[57] Abbott M.J., Edelman A.M., Turcotte L.P. (2009). CaMKK is an upstream signal of AMP-activated protein kinase in regulation of substrate metabolism in contracting skeletal muscle. Am. J. Physiol.-Regul. Integr. Comp. Physiol..

[58] Thomson D.M., Herway S.T., Fillmore N., Kim H., Brown J.D., Barrow J.R., Winder W.W. (2008). AMP-activated protein kinase phosphorylates transcription factors of the CREB family. J. Appl. Physiol..

[59] Murgia M., Toniolo L., Nagaraj N., Ciciliot S., Vindigni V., Schiaffino S., Reggiani C., Mann M. (2017). Single muscle fiber proteomics reveals fiber-type-specific features of human muscle aging. Cell Rep..

[60] Akberdin I.R., Pintus S.S., Kolpakov F.A., Vertyshev A.Y., Popov D.V. (2020). A mathematical model linking Ca^2+^-dependent signaling pathway and gene expression regulation in human skeletal muscle. Math. Biol. Bioinform..

[61] Wojtaszewski J.F., Birk J.B., Frøsig C., Holten M., Pilegaard H., Dela F. (2005). 5′-AMP activated protein kinase expression in human skeletal muscle: Effects of strength training and type 2 diabetes. J. Physiol..

[62] Rajamohan F., Reyes A.R., Frisbie R.K., Hoth L.R., Sahasrabudhe P., Magyar R., Landro J.A., Withka J.M., Caspers N.L., Calabrese M.F. (2016). Probing the enzyme kinetics, allosteric modulation and activation of α1-and α2-subunit-containing AMP-activated protein kinase (AMPK) heterotrimeric complexes by pharmacological and physiological activators. Biochem. J..

[63] Ross F.A., Jensen T.E., Hardie D.G. (2016). Differential regulation by AMP and ADP of AMPK complexes containing different γ subunit isoforms. Biochem. J..

[64] Pinter K., Grignani R.T., Watkins H., Redwood C. (2013). Localisation of AMPK γ subunits in cardiac and skeletal muscles. J. Muscle Res. Cell Motil..

[65] Birk J.B., Wojtaszewski J.F.P. (2006). Predominant α2/β2/γ3 AMPK activation during exercise in human skeletal muscle. J. Physiol..

[66] Willows R., Sanders M.J., Xiao B., Patel B.R., Martin S.R., Read J., Wilson J.R., Hubbard J., Gamblin S.J., Carling D. (2017). Phosphorylation of AMPK by upstream kinases is required for activity in mammalian cells. Biochem. J..

[67] Lizcano J.M., Göransson O., Toth R., Deak M., Morrice N.A., Boudeau J., Hawley S.A., Udd L., Mäkelä T.P., Hardie D.G. (2004). LKB1 is a master kinase that activates 13 kinases of the AMPK subfamily, including MARK/PAR-1. EMBO J..

[68] Jansen M., Ten Klooster J.P., Offerhaus G.J., Clevers H. (2009). LKB1 and AMPK family signaling: The intimate link between cell polarity and energy metabolism. Physiol. Rev..

[69] Hardie D.G. (2016). Regulation of AMP-activated protein kinase by natural and synthetic activators. Acta Pharm. Sin. B.

[70] Li J., Li S., Wang F., Xin F. (2017). Structural and biochemical insights into the allosteric activation mechanism of AMP-activated protein kinase. Chem. Biol. Drug Des..

[71] Sun P., Enslen H., Myung P.S., Maurer R.A. (1994). Differential activation of CREB by Ca^2+^/calmodulin-dependent protein kinases type II and type IV involves phosphorylation of a site that negatively regulates activity. Genes Dev..

[72] Bayer K.U., Harbers K., Schulman H. (1998). αKAP is an anchoring protein for a novel CaM kinase II isoform in skeletal muscle. EMBO J..

[73] Zong Y., Zhang C.S., Li M., Wang W., Wang Z., Hawley S.A., Ma T., Feng J.W., Tian X., Qi Q. (2019). Hierarchical activation of compartmentalized pools of AMPK depends on severity of nutrient or energy stress. Cell Res..

[74] Lira V.A., Benton C.R., Yan Z., Bonen A. (2010). PGC-1α regulation by exercise training and its influences on muscle function and insulin sensitivity. Am. J. Physiol.-Endocrinol. Metab..

[75] Pearen M.A., Muscat G.E. (2018). The nuclear receptor Nor-1 is a pleiotropic regulator of exercise-induced adaptations. Exerc. Sport Sci. Rev..

[76] Suzuki T., Maeda S., Furuhata E., Shimizu Y., Nishimura H., Kishima M., Suzuki H. (2017). A screening system to identify transcription factors that induce binding site-directed DNA demethylation. Epigenetics Chromatin.

[77] Wang S.C.M., Muscat G.E. (2013). Nuclear receptors and epigenetic signaling: Novel regulators of glycogen metabolism in skeletal muscle. IUBMB Life.

[78] Kupr B., Schnyder S., Handschin C. (2017). Role of nuclear receptors in exercise-induced muscle adaptations. Cold Spring Harb. Perspect. Med..

[79] Mahoney D.J., Parise G., Melov S., Safdar A., Tarnopolsky M.A. (2005). Analysis of global mRNA expression in human skeletal muscle during recovery from endurance exercise. FASEB J..

[80] Catoire M., Mensink M., Boekschoten M.V., Hangelbroek R., Müller M., Schrauwen P., Kersten S. (2012). Pronounced effects of acute endurance exercise on gene expression in resting and exercising human skeletal muscle. PLoS ONE.

[81] Zhang X., Odom D.T., Koo S.H., Conkright M.D., Canettieri G., Best J., Chen H., Jenner R., Herbolsheimer E., Jacobsen E. (2005). Genome-wide analysis of cAMPresponse element binding protein occupancy, phosphorylation, and target gene activation in human tissues. Proc. Natl. Acad. Sci. USA.

[82] Pattamaprapanont P., Garde C., Fabre O., Barrès R. (2016). Muscle contraction induces acute hydroxymethylation of the exercise-responsive gene Nr4a3. Front. Endocrinol..

[83] Popov D.V., Lysenko E.A., Kuzmin I.V., Vinogradova O.L., Grigoriev A.I. (2015). Regulation of PGC-1α isoform expression in skeletal muscles. Acta Nat..

[84] Hai T., Curran T. (1991). Cross-family dimerization of transcription factors Fos/Jun and ATF/CREB alters DNA binding specificity. Proc. Natl. Acad. Sci. USA.

[85] Newman J.R., Keating A.E. (2003). Comprehensive identification of human bZIP interactions with coiled-coil arrays. Science.

[86] Bottinelli R., Canepari M., Pellegrino M.A., Reggiani C. (1996). Force-velocity properties of human skeletal muscle fibres: Myosin heavy chain isoform and temperature dependence. J. Physiol..

[87] Li M., Larsson L. (2010). Force-generating capacity of human myosin isoforms extracted from single muscle fibre segments. J. Physiol..

[88] Roussel M., Mattei J.P., Le Fur Y., Ghattas B., Cozzone P.J., Bendahan D. (2003). Metabolic determinants of the onset of acidosis in exercising human muscle: A 31P-MRS study. J. Appl. Physiol..

[89] Greiner A., Esterhammer R., Bammer D., Messner H., Kremser C., Jaschke W.R., Fraedrich G., Schocke M.F. (2007). High-energy phosphate metabolism in the calf muscle of healthy humans during incremental calf exercise with and without moderate cuff stenosis. Eur. J. Appl. Physiol..

[90] Cannon D.T., Bimson W.E., Hampson S.A., Bowen T.S., Murgatroyd S.R., Marwood S., Kemp G.J., Rossiter H.B. (2014). Skeletal muscle ATP turnover by 31P magnetic resonance spectroscopy during moderate and heavy bilateral knee extension. J. Physiol..

[91] Davies M.J., Benson A.P., Cannon D.T., Marwood S., Kemp G.J., Rossiter H.B., Ferguson C. (2017). Dissociating external power from intramuscular exercise intensity during intermittent bilateral knee-extension in humans. J. Physiol..

[92] Kappenstein J., Ferrauti A., Runkel B., Fernandez-Fernandez J., Müller K., Zange J. (2013). Changes in phosphocreatine concentration of skeletal muscle during high-intensity intermittent exercise in children and adults. Eur. J. Appl. Physiol..

[93] Krustrup P., Soderlund K., Mohr M., Bangsbo J. (2004). Slow-Twitch Fiber Glycogen Depletion Elevates Moderate-Exercise Fast-Twitch Fiber Activity and O~ 2 Uptake. Med. Sci. Sports Exerc..

[94] Barker A.R., Welsman J.R., Fulford J., Welford D., Armstrong N. (2008). Muscle phosphocreatine kinetics in children and adults at the onset and offset of moderate-intensity exercise. J. Appl. Physiol..

[95] Fiedler G.B., Schmid A.I., Goluch S., Schewzow K., Laistler E., Niess F., Unger E., Wolzt M., Mirzahosseini A., Kemp G.J. (2016). Skeletal muscle ATP synthesis and cellular H+ handling measured by localized 31 P-MRS during exercise and recovery. Sci. Rep..

[96] Lee-Young R.S., Canny B.J., Myers D.E., McConell G.K. (2009). AMPK activation is fiber type specific in human skeletal muscle: Effects of exercise and short-term exercise training. J. Appl. Physiol..

[97] Kolmykov S., Yevshin I., Kulyashov M., Sharipov R., Kondrakhin Y., Makeev V.J., Kulakovskiy I.V., Kel A., Kolpakov F. (2021). GTRD: An integrated view of transcription regulation. Nucleic Acids Res..

[98] Fu M., Zhang J., Lin Y., Zhu X., Ehrengruber M.U., Chen Y.E. (2002). Early growth response factor-1 is a critical transcriptional mediator of peroxisome proliferator-activated receptor-γ1 gene expression in human aortic smooth muscle cells. J. Biol. Chem..

[99] Fu M., Zhang J., Lin Y., Zhu X., Zhao L., Ahmad M., Ehrengruber M.U., Chen Y.E. (2003). Early stimulation and late inhibition of peroxisome proliferator-activated receptor gamma (PPARgamma) gene expression by transforming growth factor beta in human aortic smooth muscle cells: Role of early growth-response factor-1 (Egr-1), activator protein 1 (AP1) and Smads. Biochem. J..

[100] Pardo P.S., Mohamed J.S., Lopez M.A., Boriek A.M. (2011). Induction of Sirt1 by mechanical stretch of skeletal muscle through the early response factor EGR1 triggers an antioxidative response. J. Biol. Chem..

[101] Lin C.Y., Lovén J., Rahl P.B., Paranal R.M., Burge C.B., Bradner J.E., Lee T.I., Young R.A. (2012). Transcriptional amplification in tumor cells with elevated c-Myc. Cell.

[102] Nie Z., Hu G., Wei G., Cui K., Yamane A., Resch W., Wang R., Green D.R., Tessarollo L., Casellas R. (2012). c-Myc is a universal amplifier of expressed genes in lymphocytes and embryonic stem cells. Cell.

[103] Rahl P.B., Young R.A. (2014). MYC and transcription elongation. Cold Spring Harb. Perspect. Med..

[104] Guccione E., Martinato F., Finocchiaro G., Luzi L., Tizzoni L., Dall’Olio V., Zardo G., Nervi C., Bernard L., Amati B. (2006). Myc-binding-site recognition in the human genome is determined by chromatin context. Nat. Cell Biol..

[105] Knoepfler P.S., Zhang X.Y., Cheng P.F., Gafken P.R., McMahon S.B., Eisenman R.N. (2006). Myc influences global chromatin structure. EMBO J..

[106] Tan Z., Luo X., Xiao L., Tang M., Bode A.M., Dong Z., Cao Y. (2016). The role of PGC1α in cancer metabolism and its therapeutic implications. Mol. Cancer Ther..

[107] Mastropasqua F., Girolimetti G., Shoshan M. (2018). PGC1α: Friend or foe in cancer?. Genes.

[108] Ahuja P., Zhao P., Angelis E., Ruan H., Korge P., Olson A., Wang Y., Jin E.S., Jeffrey F.M., Portman M. (2010). Myc controls transcriptional regulation of cardiac metabolism and mitochondrial biogenesis in response to pathological stress in mice. J. Clin. Investig..

[109] Sancho P., Burgos-Ramos E., Tavera A., Kheir T.B., Jagust P., Schoenhals M., Barneda D., Sellers K., Campos-Olivas R., Graña O. (2015). MYC/PGC-1α balance determines the metabolic phenotype and plasticity of pancreatic cancer stem cells. Cell Metab..

[110] Kim J., Saidel G.M., Cabrera M.E. (2007). Multi-scale computational model of fuel homeostasis during exercise: Effect of hormonal control. Ann. Biomed. Eng..

[111] De Luca C.J., Kline J.C. (2011). Influence of proprioceptive feedback on the firing rate and recruitment of motoneurons. J. Neural Eng..

[112] Nybo L. (2012). Brain temperature and exercise performance. Exp. Physiol..

[113] Vieira A.F., Costa R.R., Macedo R.C.O., Coconcelli L., Kruel L.F.M. (2016). Effects of aerobic exercise performed in fasted v. fed state on fat and carbohydrate metabolism in adults: A systematic review and meta-analysis. Br. J. Nutr..

[114] Aird T.P., Davies R.W., Carson B.P. (2018). Effects of fasted vs. fed-state exercise on performance and post-exercise metabolism: A systematic review and meta-analysis. Scand. J. Med. Sci. Sports.

[115] González-Alonso J., Teller C., Andersen S.L., Jensen F.B., Hyldig T., Nielsen B. (1999). Influence of body temperature on the development of fatigue during prolonged exercise in the heat. J. Appl. Physiol..

[116] Gray S.R., Soderlund K., Watson M., Ferguson R.A. (2011). Skeletal muscle ATP turnover and single fibre ATP and PCr content during intense exercise at different muscle temperatures in humans. Pflügers Arch. -Eur. J. Physiol..

[117] Prats C., Helge J.W., Nordby P., Qvortrup K., Ploug T., Dela F., Wojtaszewski J.F. (2009). Dual regulation of muscle glycogen synthase during exercise by activation and compartmentalization. J. Biol. Chem..

[118] Palm D.C., Rohwer J.M., Hofmeyr J.H.S. (2013). Regulation of glycogen synthase from mammalian skeletal muscle–a unifying view of allosteric and covalent regulation. FEBS J..

[119] Jentjens R., Jeukendrup A.E. (2003). Determinants of post-exercise glycogen synthesis during short-term recovery. Sports Med..

[120] Burke L.M., van Loon L.J., Hawley J.A. (2017). Postexercise muscle glycogen resynthesis in humans. J. Appl. Physiol..

[121] Maehlum S., Hermansen L. (1978). Muscle glycogen concentration during recovery after prolonged severe exercise in fasting subjects. Scand. J. Clin. Lab. Investig..

[122] Egan B., Zierath J.R. (2013). Exercise metabolism and the molecular regulation of skeletal muscle adaptation. Cell Metab..

[123] Mounier R., Théret M., Lantier L., Foretz M., Viollet B. (2015). Expanding roles for AMPK in skeletal muscle plasticity. Trends Endocrinol. Metab..

[124] Agudelo L.Z., Ferreira D.M., Dadvar S., Cervenka I., Ketscher L., Izadi M., Zhengye L., Furrer R., Handschin C., Venckunas T. (2019). Skeletal muscle PGC-1α1 reroutes kynurenine metabolism to increase energy efficiency and fatigue-resistance. Nat. Commun..

