# Supplementary material for: A Modular Mathematical Model of Exercise-Induced Changes in Metabolism, Signaling, and Gene Expression in Human Skeletal Muscle
Source: Int J Mol Sci. 2021 Sep 26;22(19):10353. doi: 10.3390/ijms221910353 (PMC8508736; doi:10.3390/ijms221910353)
Supplement: Supplementary file 1 [file ijms-22-10353-s001.zip › ijms-1272147-supplementary (correct file)/ijms-1272147_Supplementary material.pdf]

Supplementary material for "A Modular Mathematical Model of Exercise-Induced Changes in Metabolism, Signaling, and Gene Expression in Human Skeletal Muscle"

Table of contents

1. Rates of reactions and ODE system of the Module "Cytosol" 3
   a. SBGN scheme of the module: 3
   b. Antimony view which represents generated code according to the visual graph: 4
   c. Mathematical equations of the metabolic reactions in the Module "Cytosol": 15
2. Rates of reactions and ODE system of the Module "Mitochondrion" 22
   a. SBGN scheme of the module: 22
   b. Antimony view which represents generated code according to the visual graph: 23
   c. Mathematical equations of the metabolic reactions in the Module "Mitochondrion": 30
3. Rates of reactions and ODE system of the Module "Transport: Cytosol-Mitochondria" 36
   a. SBGN scheme of the module: 36
   b. Antimony view which represents generated code according to the visual graph: 37
   c. Mathematical equations of the metabolic reactions in the Module "Transport: Cytosol-Mitochondria": 41
4. Rates of reactions and ODE system of the Module "Capillary Blood" 43
   a. SBGN scheme of the module: 43
   b. Antimony view which represents generated code according to the visual graph: 43
   c. Mathematical equations of the metabolic reactions in the Module "Capillary Blood": 48
5. Rates of reactions and ODE system of the Module "Transport: Blood-Cytosol" 50
   d. SBGN scheme of the module: 50
   e. Antimony view which represents generated code according to the visual graph: 50
   f. Mathematical equations of the metabolic reactions in the Module "Transport: Blood-Cytosol": 54
6. Rates of reactions in the Module "Ca-signaling pathway" 56
   a. SBGN scheme of the module: 56
   b. Antimony view which represents generated code according to the visual graph: 56
   c. Mathematical equations of the metabolic reactions in the Module "Ca-signaling pathway": 60
7. Rates of reactions in the Module "AMPK" 63
   a. SBGN scheme of the module: 63
   b. Antimony view which represents generated code according to the visual graph: 65
   c. Mathematical equations of the metabolic reactions in the Module "AMPK": 70
8. Rates of reactions in the Module "Gene expression regulation" 73
   a. SBGN scheme of the module: 73

b. Antimony view which represents generated code according to the visual graph:.........................75
c. Mathematical equations of the metabolic reactions in the Module “Gene expression regulation”: 81

1. Rates of reactions and ODE system of the Module "Cytosol"

a. SBGN scheme of the module:

The Cytosol module consists of the 18 enzymatic reactions comprising glycolysis, lipid metabolism, 2 biochemical reactions (*GP*, *MGS*) of the glycogen metabolism and 3 reactions related to ATP homeostasis and catalyzed by Creatine kinase, Adenylate kinase and ATPase (Figure S1). The list of reactions and math equations describing their rates are identical in type I and II fibers, while values of the kinetic parameters are different between two types of the fibers (See Table S1 and Table S2).

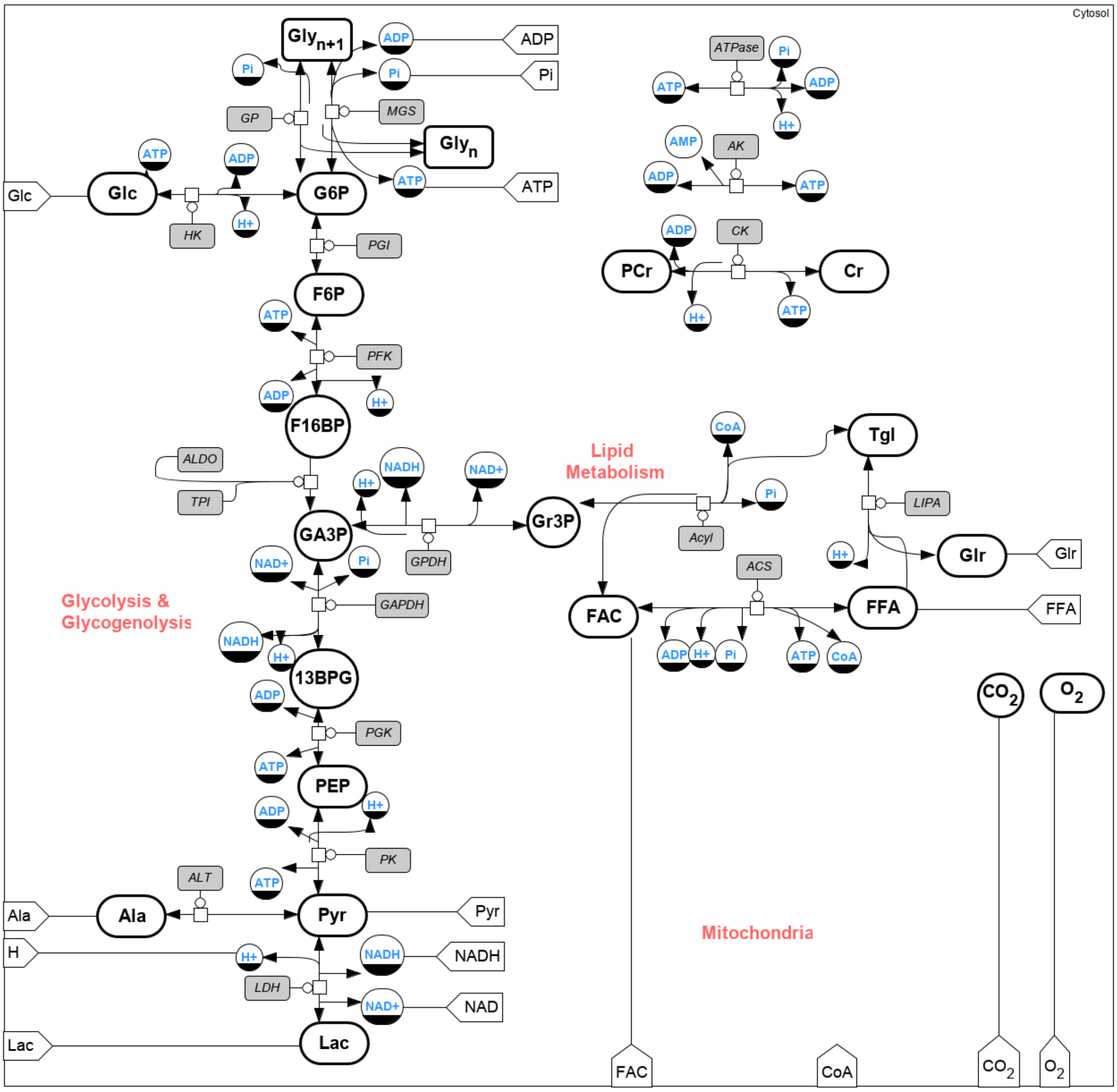

Figure S1. SBGN scheme (Le Novere et al., 2009) of the metabolic reactions taking place in the Module "Cytosol" (vertex of the bipartite graph corresponds to the biochemical reaction). The diagram of metabolic pathways comprises glycolysis and glycogenolysis, lipid metabolism and creatine kinase. Metabolites are vertices, while biochemical reactions are arcs of the diagram: Glc, glucose; G6P, glucose-6-phosphate; F6P, fructose-6-phosphate; F16BP, fructose-1,6-biphosphate; GA3P, glyceraldehyde-3-phosphate; 13BPG, 1,3-biphosphate glycerate; PEP, phosphoenolpyruvate; Pyr, pyruvate; Lac, lactate; Ala, alanine; Gr3P, glycerol-3-phosphate; Gly, glycogen; Tgl, triglycerides; Glr, glycerol; FFA, free fatty acids; FAC, fatty acyl-CoA; CoA, coenzyme-A; PCr, phosphocreatine; Cr, creatine. Enzymes catalyzing

biochemical reactions in the cytosol are depicted as rectangles on the diagram: GP, glycogen phosphorylase; MGS, glycogen synthase; HK, hexokinase; PGI, phosphoglucose isomerase; PFK, phosphofructokinase; ALDO+TPI, aldolase and triosephosphate isomerase; GAPDH, glyceraldehyde 3-phosphate dehydrogenase; PGK, phosphoglycerate kinase; PK, pyruvate kinase; LDH, lactate dehydrogenase; ALT, alanine aminotransferase; LIPA, lipases; GPDH, glycerol 3-phosphate dehydrogenase; Acyl, acyltransferase; ACS, acyl-CoA synthetase; CK, creatine kinase; AK, adenylate kinase. White arrows along the compartment border correspond to contact ports for Glc, Ala, Ala, H^+, Lac, FAC, Glr, FFA, CoA, ATP, ADP, P_i, CO_2, and O_2.

b. Antimony view which represents generated code according to the visual graph:

The conception and all details on the BioUML feature that provides an opportunity to seamlessly integrate the Antimony code (Smith et al., 2009) of the structure of studied biological systems and its mathematical model with corresponding visual representation (Kolpakov et al., 2019) are described in the main text. Antimony code generated for the corresponding visual diagram is presented below and specified for each model compartment.

```
//used mathematical functions in the module
function stressATPase_R(a,W,t_start,t)
1.0+a*W*(1.0-exp((t_start-t)/0.004));
end
function stress2(a,W,t_start,t,tau)
1.0+a*W*(1.0-exp((t_start-t)/tau));
end
function stress(a,W,t_start,t)
1.0+a*W*(1.0-exp((t_start-t)/0.4));
end
function adjustVb(K_f,K_eq,V_f,K_b,pH)
V_f*K_b/(K_f*K_eq)*10.0^(7.1-pH);
end
function MMATPase_R(r,p,v_f,k_f,v_b,k_b,a,W,t_start,t)
(v_f/k_f*r-v_b/k_b*p)/(1.0+r/k_f+p/k_b)*stressATPase_R(a,W,t_start,t);
end
function MM2(r,p,v_f,k_f,v_b,k_b,a,W,t_start,t,tau)
(v_f/k_f*r-v_b/k_b*p)/(1.0+r/k_f+p/k_b)*stress2(a,W,t_start,t,tau);
end
function MM(r,p,v_f,k_f,v_b,k_b,a,W,t_start,t)
(v_f/k_f*r-v_b/k_b*p)/(1.0+r/k_f+p/k_b)*stress(a,W,t_start,t);
end
function Kinetik(r,p,v_f,k_f,v_r,k_r,c,k,a,W,t_start,t)
c/(k+c)*MMATPase_R(r,p,v_f,k_f,v_r,k_r,a,W,t_start,t);
end

//name of the model and components
    //Function definitions
```

model
Li_2012_Cytosol_R(<ADP_free_R,<AMP_free_R,AMP,ATP,>A_10,>A_1,>A_2,ADP,ATP,Ala,CO2,CoA,FAC,FFA,Glc,Glr,H,Lac,NADH,NAD,O2,Pi,Pyr,Cytosol,<F6P_tis,<G6P_tis,H,>J_leak,<Lac_tis_R,<V_CO2_c,<V_O2_c,>V,>W,>t_start)

//the list of used compartment in the module
compartment Cytosol;

//the list of used biochemical species in the module
species ADP in Cytosol, AMP in Cytosol, ATP in Cytosol, ATPase in Cytosol, Acyl_CoA_Synthetase in Cytosol, Acyltransferase in Cytosol, Adenylate_Kinase in Cytosol, Ala in Cytosol, Alanine_Aminotransferase in Cytosol, Aldolase in Cytosol, BPG in Cytosol, CO2 in Cytosol, CoA in Cytosol, Cr in Cytosol, Creatine_Kinase in Cytosol, F16BP in Cytosol, F6P in Cytosol, FAC in Cytosol, FFA in Cytosol, G6P in Cytosol, GA3P in Cytosol, Glc in Cytosol, Glr in Cytosol, Gly_n in Cytosol, Gly_n1 in Cytosol, Glyceraldehyde_3_Phosphate_Dehydrogenase in Cytosol, Glycerol_3Phosphate_Dehydrogenase in Cytosol, Glycogen_posphorylase in Cytosol, Glycogen_synthase in Cytosol, Gr3P in Cytosol, H in Cytosol, Hexokinase in Cytosol, Lac in Cytosol, Lactate_Dehydrogenase in Cytosol, Lipase in Cytosol, NAD in Cytosol, NADH in Cytosol, O2 in Cytosol, PCr in Cytosol, PEP in Cytosol, Phosphofructokinase in Cytosol, Phosphoglucose_Isomerase in Cytosol, Phosphoglycerate_Kinase in Cytosol, Pi in Cytosol, Pyr in Cytosol, Pyruvate_Kinase in Cytosol, Tgl in Cytosol, Triose_Phosphate_Isomerase in Cytosol;

//the list of used model parameters in the module
var ADP_free_R, AMP_free_R, ATP_Glycolysis_R, ATP_Glycolysis_R_norm, ATP_hydrolysis_R_norm, A_1, A_10, A_11, A_12, A_15, A_16, A_2, A_4, A_5, A_6, A_7, A_8, A_9, Active_1, Active_15, Active_16, Active_2, Active_4, Active_5, Active_6, Active_7, Active_8, Active_9, CK_R, CK_R_norm, Cr_tis, F6P_tis, G6P_tis, Glc_tis, Gly_tis, Gly_tis_R, J_leak, K_1, K_3, K_5, K_9, K_AK, K_CK, K_b_1, K_b_10, K_b_11, K_b_12, K_b_13, K_b_14, K_b_15, K_b_16, K_b_17, K_b_18, K_b_2, K_b_3, K_b_4, K_b_5, K_b_6, K_b_7, K_b_8, K_b_9, K_eq_1, K_eq_10, K_eq_11, K_eq_12, K_eq_13, K_eq_14, K_eq_15, K_eq_16, K_eq_17, K_eq_18, K_eq_2, K_eq_3, K_eq_4, K_eq_5, K_eq_6, K_eq_7, K_eq_8, K_eq_9, K_f_1, K_f_10, K_f_11, K_f_12, K_f_13, K_f_14, K_f_15, K_f_16, K_f_17, K_f_18, K_f_2, K_f_3, K_f_4, K_f_5, K_f_6, K_f_7, K_f_8, K_f_9, Lac_Pyr, Lac_tis, Lac_tis_R, PCr_tis, PCr_tis_R, PCr_tis_normalized_R, R10_R, R11_R, R12_R, R13_R, R14_R, R15_R, R16_R, R17_R, R18_R, R1_R, R2_R, R3_R, R4_R, R5_R, R6_R, R7_R, R8_R, R9_R, Rate_H_R, Rel_AMP, V, V_CO2_c, V_O2_c, V_b_1, V_b_10, V_b_11, V_b_12, V_b_13, V_b_14, V_b_15, V_b_16, V_b_17, V_b_18, V_b_2, V_b_3, V_b_4, V_b_5, V_b_6, V_b_7, V_b_8, V_b_9, V_f_1, V_f_10, V_f_11, V_f_12, V_f_13, V_f_14, V_f_15, V_f_16, V_f_17, V_f_18, V_f_2, V_f_3, V_f_4, V_f_5, V_f_6, V_f_7, V_f_8, V_f_9, W, a, beta_cyt, k_b, k_f, p, pH, pH0, r, rate_CK_normalized_R, t, t_start, v_b, v_f;

//the list of values of used model parameters in the module

```
//Initialization
Cytosol = 3.6;
ADP = 0.14;
AMP = 0.0329280958;
ATP = 5.37709090909091;
Ala = 1.18181818181818;
BPG = 0.0909090909090909;
CO2 = 1.403;
CoA = 0.0181818181818182;
Cr = 10.0227272727273;
F16BP = 0.0681818181818182;
F6P = 0.05;
FAC = 0.00377840909090909;
FFA = 0.454545454545455;
G6P = 0.227272727272727;
GA3P = 0.0909090909090909;
Glc = 0.568181818181818;
Glr = 0.0738636363636364;
Gly_n = 86.3636363636364;
Gly_n1 = 86.3636363636364;
Gr3P = 0.170454545454545;
Lac = 0.886363636363637;
NAD = 0.184090909090909;
NADH = 3.40909090909091E-4;
O2 = 0.03375;
PCr = 20.4545454545455;
PEP = 0.00556818181818182;
Pi = 2.96875;
Pyr = 0.0539772727272727;
Tgl = 23.8636363636364;
A_1 = 0.15054;
A_11 = 0.0794936353702118;
A_15 = 0.339403112909419;
A_16 = 0.0766942747;
A_2 = 0.3394;
A_4 = 0.322075814406805;
A_5 = 0.322075814406805;
A_6 = 0.322075814406805;
A_7 = 0.322075814406805;
A_8 = 0.322075814406805;
A_9 = 0.322075814406805;
K_1 = 1.87E-5;
K_3 = 27.8;
K_5 = 0.0935;
```

K_9 = 0.00267;
K_AK = 1.05;
K_CK = 1.66E12;
K_b_1 = 293000.0;
K_b_10 = 1.3E-15;
K_b_11 = 1.44;
K_b_12 = 4.52E-15;
K_b_13 = 0.0251;
K_b_14 = 2.73E-4;
K_b_15 = 6.21E-14;
K_b_16 = 1.04E-5;
K_b_17 = 20.7;
K_b_18 = 3.18E-5;
K_b_2 = 158.0;
K_b_3 = 3.93E-7;
K_b_4 = 0.0486;
K_b_5 = 9.42E-8;
K_b_6 = 0.079;
K_b_7 = 1.96E-9;
K_b_8 = 0.0319;
K_b_9 = 0.313;
K_eq_1 = 6.94603203994856;
K_eq_10 = 1.50668628327844E9;
K_eq_11 = 2940.82095450261;
K_eq_12 = 8.75219824108104;
K_eq_13 = 3.41162352648177E8;
K_eq_14 = 3.32025516215481E8;
K_eq_15 = 374767.092470756;
K_eq_16 = 1473316.84555736;
K_eq_17 = 236927.473280989;
K_eq_18 = 0.110698588007732;
K_eq_2 = 267029.579741231;
K_eq_3 = 16263.5708908583;
K_eq_4 = 0.36678662881483;
K_eq_5 = 10331.4313649412;
K_eq_6 = 0.959629520749891;
K_eq_7 = 0.787422192473917;
K_eq_8 = 588.18148974955;
K_eq_9 = 1.50085452944024E9;
K_f_1 = 0.00306;
K_f_10 = 1.17E23;
K_f_11 = 0.0528;
K_f_12 = 16.7;
K_f_13 = 1.96E-9;
K_f_14 = 8.42E-9;

```
K_f_15 = 0.312;
K_f_16 = 5.92;
K_f_17 = 2.97E-4;
K_f_18 = 132.0;
K_f_2 = 17400.0;
K_f_3 = 3.29;
K_f_4 = 0.278;
K_f_5 = 0.288;
K_f_6 = 0.667;
K_f_7 = 0.0387;
K_f_8 = 0.00158;
K_f_9 = 7.61E-9;
V = 2.0;
V_b_1 = 3495359.76982217;
V_b_10 = 0.00360094629005659;
V_b_11 = 8.44479777448805E-4;
V_b_12 = 2.76847914338608E-19;
V_b_13 = 4.35407827932216E-4;
V_b_14 = 1.30640986602748E-6;
V_b_15 = 2.28979336515257E-18;
V_b_16 = 3.75711116210484E-11;
V_b_17 = 58.8340125624107;
V_b_18 = 4.35252328407772E-4;
V_b_2 = 2.35535321994023E-6;
V_b_3 = 6.49938886232676E-12;
V_b_4 = 0.404289985456642;
V_b_5 = 1.2673541143752E-10;
V_b_6 = 0.162611317352597;
V_b_7 = 3.38726454788882E-8;
V_b_8 = 0.0081667461616334;
V_b_9 = 0.00674862713374315;
V_f_1 = 0.253561009263262;
V_f_10 = 4.88294674384557E44;
V_f_11 = 0.091060340256422;
V_f_12 = 0.00895233733251904;
V_f_13 = 0.0115995110565746;
V_f_14 = 0.0133782823205861;
V_f_15 = 4.31142400912918;
V_f_16 = 31.5092863281761;
V_f_17 = 200.0;
V_f_18 = 200.0;
V_f_2 = 69.2640016459027;
V_f_3 = 0.884895071926041;
V_f_4 = 0.848232689517042;
V_f_5 = 4.0031333649151;
```

V_f_6 = 1.31750754303672;
V_f_7 = 0.52663735516167;
V_f_8 = 0.237917733527724;
V_f_9 = 0.246260271070265;
beta_cyt = 6.65774451662929;
pH0 = 7.1;

//the list of reactions in the module

//Reactions
R1: Gly_n1 + Pi_1_R1 -> G6P + Gly_n; R1_R/Cytosol;
R1_mod1: Glycogen_posphorylase-(R1;
R10: H + NADH + Pyr -> Lac + NAD; R10_R/Cytosol;
R11_mod: Lactate_Dehydrogenase-(R10;
R11: Pyr -> Ala; R11_R/Cytosol;
R11_mod1: Alanine_Aminotransferase-(R11;
R12: Tgl -> 3FFA + Glr + 3H_1_R12; R12_R/Cytosol;
R12_mod1: Lipase-(R12;
R13: GA3P + H_1_R13 + NADH_1_R13 -> Gr3P + NAD_1_R13; R13_R/Cytosol;
R13_mod1: Glycerol_3Phosphate_Dehydrogenase-(R13;
R14: 3FAC + Gr3P -> 3CoA + Pi_1_R14 + Tgl; R14_R/Cytosol;
R14_mod1: Acyltransferase-(R14;
R15: 2ATP_1_R15 + CoA_1_R15 + FFA -> 2ADP_1_R15 + FAC + 2H_1_R15 + 2Pi_1_R15; R15_R/Cytosol;
R15_mod1: Acyl_CoA_Synthetase-(R15;
R16: ATP_1_R16 -> ADP_1_R16 + H_1_R16 + Pi_1_R16; R16_R/Cytosol;
R16_mod1: ATPase-(R16;
R17: ADP_1_R17 + H_1_R17 + PCr -> ATP_1_R17 + Cr; R17_R/Cytosol;
R17_mod1: Creatine_Kinase-(R17;
R18: AMP + ATP_1_R18 -> 2ADP_1_R18; R18_R/Cytosol;
R18_mod1: Adenylate_Kinase-(R18;
R2: ATP + G6P + Gly_n -> ADP + Gly_n1 + 2Pi; R2_R/Cytosol;
R2_mod1: Glycogen_synthase-(R2;
R3: ATP_1_R3 + Glc -> ADP_1_R3 + G6P + H_1_R3; R3_R/Cytosol;
R3_mod1: Hexokinase-(R3;
R4: G6P -> F6P; R4_R/Cytosol;
R4_mod1: Phosphoglucose_Isomerase-(R4;
R5: ATP_1_R5 + F6P -> ADP_1_R5 + F16BP + H_1_R5; R5_R/Cytosol;
R5_mod1: Phosphofructokinase-(R5;
R7: GA3P + NAD_1_R7 + Pi_1_R7 -> BPG + H_1_R7 + NADH_1_R7; R7_R/Cytosol;
R7_mod1: Glyceraldehyde_3_Phosphate_Dehydrogenase-(R7;
R8: ADP_1_R8 + BPG -> ATP_1_R8 + PEP; R8_R/Cytosol;
R8_mod1: Phosphoglycerate_Kinase-(R8;
R9: ADP_1_R9 + H_1_R9 + PEP -> ATP_1_R9 + Pyr; R9_R/Cytosol;
R9_mod1: Pyruvate_Kinase-(R9;

```
  reaction: F16BP => 2GA3P; R6_R/Cytosol;
  reaction_mod1: Aldolase-( reaction;
  reaction_mod1: Triose_Phosphate_Isomerase-( reaction;
```

//the list of used mathematical equations in the module

```
 //Equations
  Rel_AMP := AMP/ATP;
  V_CO2_c := Cytosol;
  R9_R :=
Kinetik(ADP*H*PEP,ATP*Pyr,V_f_9,K_f_9,V_b_9,K_b_9,F16BP,K_9,A_9,W,t_start,time);
  R6_R := MM(F16BP,GA3P^2.0,V_f_6,K_f_6,V_b_6,K_b_6,A_6,W,t_start,time);
  R7_R :=
MM(GA3P*NAD*Pi,BPG*H*NADH,V_f_7,K_f_7,V_b_7,K_b_7,A_7,W,t_start,time);
  R8_R := MM(ADP*BPG,ATP*PEP,V_f_8,K_f_8,V_b_8,K_b_8,A_8,W,t_start,time);
  R10_R :=
MM(H*NADH*Pyr,Lac*NAD,V_f_10,K_f_10,V_b_10,K_b_10,A_10,W,t_start,time);
  R16_R :=
MMATPase_R(ATP,ADP*H*Pi,V_f_16,K_f_16,V_b_16,K_b_16,A_16,W,t_start,time);
  Lac_Pyr := Lac/Pyr;
  Cr_tis := Cytosol*Cr/V;
  PCr_tis_R := Cytosol*PCr/V;
  Glc_tis := Cytosol*Glc/V;
  H = 10.0^(-pH0+3.0);
  G6P_tis := Cytosol*G6P/V;
  F6P_tis := Cytosol*F6P/V;
  Gly_tis_R := Cytosol*Gly_n1/V;
  R1_R :=
Kinetik(Gly_n1*Pi,G6P*Gly_n,V_f_1,K_f_1,V_b_1,K_b_1,Rel_AMP,K_1,A_1,W,t_start,time);
  R2_R :=
MM2(ATP*G6P*Gly_n,ADP*Gly_n1*Pi^2.0,V_f_2,K_f_2,V_b_2,K_b_2,A_2,W,t_start,time,2.0);
  R11_R := MM(Pyr,Ala,V_f_11,K_f_11,V_b_11,K_b_11,A_11,W,t_start,time);
  R12_R :=
MM(Tgl,FFA^3.0*Glr*H^3.0,V_f_12,K_f_12,V_b_12,K_b_12,A_12,W,t_start,time);
  R13_R :=
MM(GA3P*H*NADH,Gr3P*NAD,V_f_13,K_f_13,V_b_13,K_b_13,0.0,W,t_start,time);
  Lac_tis_R := Cytosol*Lac/V;
  R14_R :=
MM(FAC^3.0*Gr3P,CoA^3.0*Pi*Tgl,V_f_14,K_f_14,V_b_14,K_b_14,0.0,W,t_start,time);
  V_O2_c := Cytosol;
```

```
  R15_R :=
MM(ATP^2.0*CoA*FFA,ADP^2.0*FAC*H^2.0*Pi^2.0,V_f_15,K_f_15,V_b_15,K_b_1
5,A_15,W,t_start,time);
  R17_R :=
MM(ADP*H*PCr,ATP*Cr,V_f_17,K_f_17,V_b_17,K_b_17,0.0,W,t_start,time);
  R18_R :=
MM(AMP*ATP,ADP^2.0,V_f_18,K_f_18,V_b_18,K_b_18,0.0,W,t_start,time);
  Active_1 := stress(A_1,W,t_start,time);
  Active_2 := stress2(A_2,W,t_start,time,2.0);
  Active_4 := stress(A_4,W,t_start,time);
  Active_5 := stress(A_5,W,t_start,time);
  Active_6 := stress(A_6,W,t_start,time);
  Active_7 := stress(A_7,W,t_start,time);
  Active_8 := stress(A_8,W,t_start,time);
  H '= Rate_H_R/(Cytosol*1000.0);
  Active_9 := stress(A_9,W,t_start,time);
  Active_15 := stress(A_15,W,t_start,time);
  Active_16 := stress(A_16,W,t_start,time);
  rate_CK_normalized_R := R17_R/Cytosol;
  PCr_tis_normalized_R := PCr_tis/Cytosol;
  ADP_free_R := ATP*Cr/(PCr*H*K_CK);
  AMP_free_R := ADP_free_R^2.0/(ATP*K_AK);
  ATP_Glycolysis_R := 5.0*(R9_R+R8_R-R5_R-R3_R-R10_R);
  CK_R := 5.0*R17_R;
  ATP_Glycolysis_R_norm := 5.0*(R9_R+R8_R-R5_R-R3_R-R10_R)/Cytosol;
  Cytosol := 0.88*V;
  CK_R_norm := 5.0*R17_R/Cytosol;
  ATP_hydrolysis_R_norm := R16_R/Cytosol;
  V_b_1 := adjustVb(K_f_1,K_eq_1,V_f_1,K_b_1,pH);
  V_b_2 := adjustVb(K_f_2,K_eq_2,V_f_2,K_b_2,pH);
  V_b_3 := adjustVb(K_f_3,K_eq_3,V_f_3,K_b_3,pH);
  V_b_4 := adjustVb(K_f_4,K_eq_4,V_f_4,K_b_4,pH);
  V_b_5 := adjustVb(K_f_5,K_eq_5,V_f_5,K_b_5,pH);
  V_b_7 := adjustVb(K_f_7,K_eq_7,V_f_7,K_b_7,pH);
  V_b_8 := adjustVb(K_f_8,K_eq_8,V_f_8,K_b_8,pH);
  Rate_H_R := 2.303/beta_cyt*H*(R3_R+R5_R+R7_R-R9_R-R10_R+3.0*R12_R-
R13_R+2.0*R15_R+R16_R-R17_R-J_leak);
  V_b_9 := adjustVb(K_f_9,K_eq_9,V_f_9,K_b_9,pH);
  V_b_10 := adjustVb(K_f_10,K_eq_10,V_f_10,K_b_10,pH);
  V_b_11 := adjustVb(K_f_11,K_eq_11,V_f_11,K_b_11,pH);
  V_b_12 := adjustVb(K_f_12,K_eq_12,V_f_12,K_b_12,pH);
  V_b_13 := adjustVb(K_f_13,K_eq_13,V_f_13,K_b_13,pH);
  V_b_14 := adjustVb(K_f_14,K_eq_14,V_f_14,K_b_14,pH);
  V_b_15 := adjustVb(K_f_15,K_eq_15,V_f_15,K_b_15,pH);
  V_b_16 := adjustVb(K_f_16,K_eq_16,V_f_16,K_b_16,pH);
```

```
V_b_6 := adjustVb(K_f_6,K_eq_6,V_f_6,K_b_6,pH);
V_b_17 := adjustVb(K_f_17,K_eq_17,V_f_17,K_b_17,pH);
R3_R := Kinetik(ATP*Glc,ADP*G6P*H,V_f_3,K_f_3,V_b_3,K_b_3,K_3,G6P,0.0,W,t_start,time);
V_b_18 := adjustVb(K_f_18,K_eq_18,V_f_18,K_b_18,pH);
R5_R := Kinetik(ATP*F6P,ADP*F16BP*H,V_f_5,K_f_5,V_b_5,K_b_5,AMP,K_5,A_5,W,t_start,time);
R4_R := MM(G6P,F6P,V_f_4,K_f_4,V_b_4,K_b_4,A_4,W,t_start,time);
pH := 3.0-log(H,10.0);
```

//the list of used model species and their alias

```
//Titles
ADP_1_R15 is "ADP";
ADP_1_R16 is "ADP";
ADP_1_R17 is "ADP";
ADP_1_R18 is "ADP";
ADP_1_R3 is "ADP";
ADP_1_R5 is "ADP";
ADP_1_R8 is "ADP";
ADP_1_R9 is "ADP";
ATP_1_R15 is "ATP";
ATP_1_R16 is "ATP";
ATP_1_R17 is "ATP";
ATP_1_R18 is "ATP";
ATP_1_R3 is "ATP";
ATP_1_R5 is "ATP";
ATP_1_R8 is "ATP";
ATP_1_R9 is "ATP";
Acyl_CoA_Synthetase is "ACS";
Acyltransferase is "Acyl";
Adenylate_Kinase is "AK";
Alanine_Aminotransferase is "ALT";
Aldolase is "ALDO";
BPG is "13BPG";
CO2 is "CO<sub>2";
CoA_1_R15 is "CoA";
Creatine_Kinase is "CK";
Gly_n is "Gly<sub>n</sub>";
Gly_n1 is "Gly<sub>n+1</sub>";
Glyceraldehyde_3_Phosphate_Dehydrogenase is "GAPDH";
Glycerol_3Phosphate_Dehydrogenase is "GPDH";
Glycogen_posphorylase is "GP";
Glycogen_synthase is "MGS";
```

```
H is "H+";
H_1_R12 is "H+";
H_1_R13 is "H+";
H_1_R15 is "H+";
H_1_R16 is "H+";
H_1_R17 is "H+";
H_1_R3 is "H+";
H_1_R5 is "H+";
H_1_R7 is "H+";
H_1_R9 is "H+";
Hexokinase is "HK";
Lactate_Dehydrogenase is "LDH";
Lipase is "LIPA";
NAD is "NAD+";
NADH_1_R13 is "NADH";
NADH_1_R7 is "NADH";
NAD_1_R13 is "NAD+";
NAD_1_R7 is "NAD+";
O2 is "O<sub>2";
Phosphofructokinase is "PFK";
Phosphoglucose_Isomerase is "PGI";
Phosphoglycerate_Kinase is "PGK";
Pi_1_R1 is "Pi";
Pi_1_R14 is "Pi";
Pi_1_R15 is "Pi";
Pi_1_R16 is "Pi";
Pi_1_R7 is "Pi";
Pyruvate_Kinase is "PK";
Triose_Phosphate_Isomerase is "TPI";

//SBGN Properties
@ADP.sbgnType = "simple chemical";
@AMP.sbgnType = "simple chemical";
@ATP.sbgnType = "simple chemical";
@ATPase.sbgnType = "macromolecule";
@ATPase.sbgnViewTitle = "ATPase";
@Acyl_CoA_Synthetase.sbgnType = "macromolecule";
@Acyl_CoA_Synthetase.sbgnViewTitle = "Acyl_CoA_Synthetase";
@Acyltransferase.sbgnType = "macromolecule";
@Acyltransferase.sbgnViewTitle = "Acyltransferase";
@Adenylate_Kinase.sbgnType = "macromolecule";
@Adenylate_Kinase.sbgnViewTitle = "Adenylate_Kinase";
@Ala.sbgnType = "simple chemical";
@Alanine_Aminotransferase.sbgnType = "macromolecule";
@Alanine_Aminotransferase.sbgnViewTitle = "Alanine_Aminotransferase";
```

```
@Aldolase.sbgnType = "macromolecule";
@Aldolase.sbgnViewTitle = "Aldolase";
@BPG.sbgnType = "simple chemical";
@CO2.sbgnType = "simple chemical";
@CoA.sbgnType = "simple chemical";
@Cr.sbgnType = "simple chemical";
@Creatine_Kinase.sbgnType = "macromolecule";
@Creatine_Kinase.sbgnViewTitle = "Creatine_Kinase";
@F16BP.sbgnType = "simple chemical";
@F6P.sbgnType = "simple chemical";
@FAC.sbgnType = "simple chemical";
@FFA.sbgnType = "simple chemical";
@G6P.sbgnType = "simple chemical";
@GA3P.sbgnType = "simple chemical";
@Glc.sbgnType = "simple chemical";
@Glr.sbgnType = "simple chemical";
@Gly_n.sbgnType = "macromolecule";
@Gly_n.sbgnViewTitle = "Gly_n";
@Gly_n1.sbgnType = "macromolecule";
@Gly_n1.sbgnViewTitle = "Gly_n1";
@Glyceraldehyde_3_Phosphate_Dehydrogenase.sbgnType = "macromolecule";
@Glyceraldehyde_3_Phosphate_Dehydrogenase.sbgnViewTitle =
"Glyceraldehyde_3_Phosphate_Dehydrogenase";
@Glycerol_3Phosphate_Dehydrogenase.sbgnType = "macromolecule";
@Glycerol_3Phosphate_Dehydrogenase.sbgnViewTitle =
"Glycerol_3Phosphate_Dehydrogenase";
@Glycogen_posphorylase.sbgnType = "macromolecule";
@Glycogen_posphorylase.sbgnViewTitle = "Glycogen_posphorylase";
@Glycogen_synthase.sbgnType = "macromolecule";
@Glycogen_synthase.sbgnViewTitle = "Glycogen_synthase";
@Gr3P.sbgnType = "simple chemical";
@H.sbgnType = "simple chemical";
@Hexokinase.sbgnType = "macromolecule";
@Hexokinase.sbgnViewTitle = "Hexokinase";
@Lac.sbgnType = "simple chemical";
@Lactate_Dehydrogenase.sbgnType = "macromolecule";
@Lactate_Dehydrogenase.sbgnViewTitle = "Lactate_Dehydrogenase";
@Lipase.sbgnType = "macromolecule";
@Lipase.sbgnViewTitle = "Lipase";
@NAD.sbgnType = "simple chemical";
@NADH.sbgnType = "simple chemical";
@O2.sbgnType = "simple chemical";
@PCr.sbgnType = "simple chemical";
@PEP.sbgnType = "simple chemical";
@Phosphofructokinase.sbgnType = "macromolecule";
```

```
@Phosphofructokinase.sbgnViewTitle = "Phosphofructokinase";
@Phosphoglucose_Isomerase.sbgnType = "macromolecule";
@Phosphoglucose_Isomerase.sbgnViewTitle = "Phosphoglucose_Isomerase";
@Phosphoglycerate_Kinase.sbgnType = "macromolecule";
@Phosphoglycerate_Kinase.sbgnViewTitle = "Phosphoglycerate_Kinase";
@Pi.sbgnType = "simple chemical";
@Pyr.sbgnType = "simple chemical";
@Pyruvate_Kinase.sbgnType = "macromolecule";
@Pyruvate_Kinase.sbgnViewTitle = "Pyruvate_Kinase";
@Tgl.sbgnType = "simple chemical";
@Triose_Phosphate_Isomerase.sbgnType = "macromolecule";
@Triose_Phosphate_Isomerase.sbgnViewTitle = "Triose_Phosphate_Isomerase";
end
```

c. Mathematical equations of the metabolic reactions in the Module "Cytosol":

The biochemical reaction flux expressions and used kinetic laws of the reactions in the Module "Cytosol" are originated from Li et al., 2012. According to the original paper, the flux expressions (F_i) for enzymatic reactions include the next set of parameters: $V_{max}^{f_i}$ and $V_{max}^{b_i}$ are the maximal forward and backward *i*- reaction velocities, respectively, while $K_m^{f_i}$ and $K_m^{b_i}$ are the corresponding Michaelis-Menten constants. Some flux expressions ($F_{GP}, F_{HK}, F_{PFK}, F_{PK}$) in the Module account for activation and/or inhibition of the metabolic reaction flux by intermediate metabolites in the cytosolic metabolism, where C_i is the metabolite's concentration and $K_m^{Reg_i}$ is the activation or inhibition constant of the regulatory metabolite in the *i* reaction. In this model version, the rate of each metabolic and transport reaction is multiplied by a linear function of the work rate (W) parameter which defines power of the physical exercise to consider activation mechanisms related to an exercises response: $Function(W) = 1 + \alpha_i * W * (1 - e^{\frac{t_{start}-t}{\tau_i}})$, where α_i is the activation coefficient, W is the work rate value, τ_i indicates the time constant of metabolic reaction rates change in response to exercise, while t_{start} – the simulation time when an exercise is started. Thus, flux expressions for the metabolic reactions in the Module "Cytosol" represents by the next set of equations:

R1. Glycogen Phosphorylase (as GP is shown in Figure S1)

$$F_{GP} = \left(\frac{\frac{C_{AMP}}{C_{ATP}}}{K_m^{RegGP} + \frac{C_{AMP}}{C_{ATP}}} \right) * \left(\frac{\frac{V_{max}^{fGP}}{K_m^{fGP}} * C_{Gly_{n+1}} * C_{P_i} - \frac{V_{max}^{bGP}}{K_m^{bGP}} * C_{Gly_n} * C_{G6P}}{1 + \frac{C_{Gly_{n+1}} * C_{P_i}}{K_m^{fGP}} + \frac{C_{Gly_n} * C_{G6P}}{K_m^{bGP}}} \right) * (1 + \alpha_{GP} * W * (1 - e^{\frac{t_{start}-t}{\tau_{GP}}}))$$

R2. Glycogen Synthase (as MGS is shown in Figure S1)

$$F_{MGS} = \left(\frac{\frac{V_{max}^{f_{MGS}}}{K_m^{f_{MGS}}} * C_{Gly_n} * C_{G6P} * C_{ATP} - \frac{V_{max}^{b_{MGS}}}{K_m^{b_{MGS}}} * C_{Gly_{n+1}} * {C_{P_i}}^2 * C_{ADP}}{1 + \frac{C_{Gly_n} * C_{G6P} * C_{ATP}}{K_m^{f_{MGS}}} + \frac{C_{Gly_{n+1}} * {C_{P_i}}^2 * C_{ADP}}{K_m^{b_{MGS}}}} \right) * (1 + \alpha_{MGS} * W * (1 - e^{\frac{t_{start}-t}{\tau_{MGS}}}))$$

R3. Hexokinase (as HK is shown in Figure S1)

$$F_{HK} = \left(\frac{K_m^{Reg_{HK}}}{K_m^{Reg_{HK}} + C_{G6P}} \right) * \left(\frac{\frac{V_{max}^{f_{HK}}}{K_m^{f_{HK}}} * C_{Glc} * C_{ATP} - \frac{V_{max}^{b_{HK}}}{K_m^{b_{HK}}} * C_{G6P} * C_{ADP} * C_{H^+}}{1 + \frac{C_{Glc} * C_{ATP}}{K_m^{f_{HK}}} + \frac{C_{G6P} * C_{ADP} * C_{H^+}}{K_m^{b_{HK}}}} \right) * (1 + \alpha_{HK} * W * (1 - e^{\frac{t_{start}-t}{\tau_{HK}}}))$$

R4. Phosphoglucose Isomerase (as PGI is shown in Figure S1)

$$F_{PGI} = \left(\frac{\frac{V_{max}^{f_{PGI}}}{K_m^{f_{PGI}}} * C_{G6P} - \frac{V_{max}^{b_{PGI}}}{K_m^{b_{PGI}}} * C_{F6P}}{1 + \frac{C_{G6P}}{K_m^{f_{PGI}}} + \frac{C_{F6P}}{K_m^{b_{PGI}}}} \right) * (1 + \alpha_{PGI} * W * (1 - e^{\frac{t_{start}-t}{\tau_{PGI}}}))$$

R5. Phosphofructokinase (as PFK is shown in Figure S1)

$$F_{PFK} = \left(\frac{C_{AMP}}{K_m^{Reg_{PFK}} + C_{AMP}} \right) * \left(\frac{\frac{V_{max}^{f_{PFK}}}{K_m^{f_{PFK}}} * C_{F6P} * C_{ATP} - \frac{V_{max}^{b_{PFK}}}{K_m^{b_{PFK}}} * C_{F16BP} * C_{ADP} * C_{H^+}}{1 + \frac{C_{F6P} * C_{ATP}}{K_m^{f_{PFK}}} + \frac{C_{F16BP} * C_{ADP} * C_{H^+}}{K_m^{b_{PFK}}}} \right) * (1 + \alpha_{PFK} * W * (1 - e^{\frac{t_{start}-t}{\tau_{PFK}}}))$$

R6. Glyceraldehyde 3-Phosphate Formation (as ALDO and TPI is shown in Figure S1)

$$F_{ALDO+TPI} = \left(\frac{\frac{V_{max}^{f_{ALDO+TPI}}}{K_m^{f_{ALDO+TPI}}} * C_{F16BP} - \frac{V_{max}^{b_{ALDO+TPI}}}{K_m^{b_{ALDO+TPI}}} * C_{GA3P}^2}{1 + \frac{C_{F16BP}}{K_m^{f_{ALDO+TPI}}} + \frac{C_{F6P}}{K_m^{b_{ALDO+TPI}}}} \right) * (1 + \alpha_{ALDO+TPI} * W * (1 - e^{\frac{t_{start}-t}{\tau_{ALDO+TPI}}}))$$

R7. Glyceraldehyde 3-Phosphate Dehydrogenase (as GAPDH is shown in Figure S1)

$$F_{GAPDH} = \left(\frac{\frac{V_{max}^{f_{GAPDH}}}{K_m^{f_{GAPDH}}} * C_{GA3P} * C_{P_i} * C_{NAD^+} - \frac{V_{max}^{b_{GAPDH}}}{K_m^{b_{GAPDH}}} * C_{13BPG} * C_{NADH} * C_{H^+}}{1 + \frac{C_{GA3P} * C_{P_i} * C_{NAD^+}}{K_m^{f_{GAPDH}}} + \frac{C_{13BPG} * C_{NADH} * C_{H^+}}{K_m^{b_{GAPDH}}}} \right) * (1 + \alpha_{GAPDH} * W * (1 - e^{\frac{t_{start}-t}{\tau_{GAPDH}}}))$$

R8. Phosphoglycerate Kinase (as PGK is shown in Figure S1)

$$F_{PGK} = \left(\frac{\frac{V_{max}^{f_{PGK}}}{K_m^{f_{PGK}}} * C_{13BPG} * C_{ADP} - \frac{V_{max}^{b_{PGK}}}{K_m^{b_{PGK}}} * C_{PEP} * C_{ATP}}{1 + \frac{C_{13BPG} * C_{ADP}}{K_m^{f_{PGK}}} + \frac{C_{PEP} * C_{ATP}}{K_m^{b_{PGK}}}} \right) * (1 + \alpha_{PGK} * W * (1 - e^{\frac{t_{start}-t}{\tau_{PGK}}}))$$

R9. Pyruvate Kinase (as PK is shown in Figure S1)

$$F_{PK} = \left(\frac{C_{F16BP}}{K_m^{Reg_{PK}} + C_{F16BP}} \right) * \left(\frac{\frac{V_{max}^{f_{PK}}}{K_m^{f_{PK}}} * C_{PEP} * C_{ADP} * C_{H^+} - \frac{V_{max}^{b_{PK}}}{K_m^{b_{PK}}} * C_{Pyr} * C_{ATP}}{1 + \frac{C_{PEP} * C_{ADP} * C_{H^+}}{K_m^{f_{PK}}} + \frac{C_{Pyr} * C_{ATP}}{K_m^{b_{PK}}}} \right) * (1 + \alpha_{PK} * W * (1 - e^{\frac{t_{start}-t}{\tau_{PK}}}))$$

R10. Lactate Dehydrogenase (as LDH is shown in Figure S1)

$$F_{LDH} = \left(\frac{\frac{V_{max}^{f_{LDH}}}{K_m^{f_{LDH}}} * C_{Pyr} * C_{NADH} * C_{H^+} - \frac{V_{max}^{b_{LDH}}}{K_m^{b_{LDH}}} * C_{Lac} * C_{NAD^+}}{1 + \frac{C_{Pyr} * C_{NADH} * C_{H^+}}{K_m^{f_{LDH}}} + \frac{C_{Lac} * C_{NAD^+}}{K_m^{b_{LDH}}}} \right) * (1 + \alpha_{LDH} * W * (1 - e^{\frac{t_{start} - t}{\tau_{LDH}}}))$$

R11. Alanine Aminotransferase (as ALT is shown in Figure S1)

$$F_{ALT} = \left(\frac{\frac{V_{max}^{f_{ALT}}}{K_m^{f_{ALT}}} * C_{Pyr} - \frac{V_{max}^{b_{ALT}}}{K_m^{b_{ALT}}} * C_{Ala}}{1 + \frac{C_{Pyr}}{K_m^{f_{ALT}}} + \frac{C_{Ala}}{K_m^{b_{ALT}}}} \right) * (1 + \alpha_{ALT} * W * (1 - e^{\frac{t_{start} - t}{\tau_{ALT}}}))$$

R12. Lipases (as LIPA is shown in Figure S1)

$$F_{LIPA} = \left(\frac{\frac{V_{max}^{f_{LIPA}}}{K_m^{f_{LIPA}}} * C_{Tgl} - \frac{V_{max}^{b_{LIPA}}}{K_m^{b_{LIPA}}} * C_{Glr} * C_{FFA}^3 * C_{H^+}^3}{1 + \frac{C_{Tgl}}{K_m^{f_{LIPA}}} + \frac{C_{Glr} * C_{FFA}^3 * C_{H^+}^3}{K_m^{b_{LIPA}}}} \right) * (1 + \alpha_{LIPA} * W * (1 - e^{\frac{t_{start} - t}{\tau_{LIPA}}}))$$

R13. Glycerol 3-Phosphate Dehydrogenase (as GPDH is shown in Figure S1)

$$F_{GPDH} = \left(\frac{\frac{V_{max}^{f_{GPDH}}}{K_m^{f_{GPDH}}} * C_{GA3P} * C_{NADH} * C_{H^+} - \frac{V_{max}^{b_{GPDH}}}{K_m^{b_{GPDH}}} * C_{Gr3P} * C_{NAD^+}}{1 + \frac{C_{GA3P} * C_{NADH} * C_{H^+}}{K_m^{f_{GPDH}}} + \frac{C_{Gr3P} * C_{NAD^+}}{K_m^{b_{GPDH}}}} \right) * (1 + \alpha_{GPDH} * W * (1 - e^{\frac{t_{start} - t}{\tau_{GPDH}}}))$$

R14. Acyltransferase (as Acyl is shown in Figure S1)

$$F_{Acyl} = \left(\frac{\frac{V_{max}^{f_{Acyl}}}{K_m^{f_{Acyl}}} * C_{Gr3P} * C_{FAC}^3 - \frac{V_{max}^{b_{Acyl}}}{K_m^{b_{Acyl}}} * C_{Tgl} * C_{CoA}^3 * C_{P_i}}{1 + \frac{C_{Gr3P} * C_{FAC}^3}{K_m^{f_{Acyl}}} + \frac{C_{Tgl} * C_{CoA}^3 * C_{P_i}}{K_m^{b_{Acyl}}}} \right) * (1 + \alpha_{Acyl} * W * (1 - e^{\frac{t_{start} - t}{\tau_{Acyl}}}))$$

R15. Acyl-CoA Synthetase (as ACS is shown in Figure S1)

$$F_{ACS} = \left(\frac{\frac{V_{max}^{f_{ACS}}}{K_m^{f_{ACS}}} * C_{FFA} * C_{CoA} * C_{ATP}^2 - \frac{V_{max}^{b_{ACS}}}{K_m^{b_{ACS}}} * C_{FAC} * C_{ADP}^2 * C_{P_i}^2 * C_{H^+}^2}{1 + \frac{C_{FFA} * C_{CoA} * C_{ATP}^2}{K_m^{f_{ACS}}} + \frac{C_{FAC} * C_{ADP}^2 * C_{P_i}^2 * C_{H^+}^2}{K_m^{b_{ACS}}}} \right) * (1 + \alpha_{ACS} * W * (1 - e^{\frac{t_{start} - t}{\tau_{ACS}}}))$$

R16. ATPase (as ATPase is shown in Figure S1)

$$F_{ATPase} = \left(\frac{\frac{V_{max}^{f_{ATPase}}}{K_m^{f_{ATPase}}} * C_{ATP} - \frac{V_{max}^{b_{ATPase}}}{K_m^{b_{ATPase}}} * C_{ADP} * C_{P_i} * C_{H^+}}{1 + \frac{C_{ATP}}{K_m^{f_{ATPase}}} + \frac{C_{ADP} * C_{P_i} * C_{H^+}}{K_m^{b_{ATPase}}}} \right) * (1 + \alpha_{ATPase} * W * (1 - e^{\frac{t_{start} - t}{\tau_{ATPase}}}))$$

R17. Creatine Kinase (as CK is shown in Figure S1)

$$F_{CK} = \left(\frac{\frac{V_{max}^{f_{CK}}}{K_m^{f_{CK}}} * C_{PCr} * C_{ADP} * C_{H^+} - \frac{V_{max}^{b_{CK}}}{K_m^{b_{CK}}} * C_{Cr} * C_{ATP}}{1 + \frac{C_{PCr} * C_{ADP} * C_{H^+}}{K_m^{f_{CK}}} + \frac{C_{Cr} * C_{ATP}}{K_m^{b_{CK}}}} \right) * (1 + \alpha_{CK} * W * (1 - e^{\frac{t_{start} - t}{\tau_{CK}}}))$$

R18. Adenylate Kinase (as AK is shown in Figure S1)

$$F_{AK} = \left(\frac{\frac{V_{max}^{f_{AK}}}{K_m^{f_{AK}}} * C_{ATP} * C_{AMP} - \frac{V_{max}^{b_{AK}}}{K_m^{b_{AK}}} * C_{ADP}^2}{1 + \frac{C_{ATP} * C_{AMP}}{K_m^{f_{AK}}} + \frac{C_{ADP}^2}{K_m^{b_{AK}}}} \right) * (1 + \alpha_{AK} * W * (1 - e^{\frac{t_{start} - t}{\tau_{AK}}}))$$

According to the above-mentioned rate equations and transport fluxes both between cytosol - mitochondria ($T_{cyt<->mit,type,i}^{f\ or\ p}$, where superscripts f and p denote facilitated and passive transports, respectively), $type \in (type\ I\ fiber\ (R), type\ II\ fiber(W))$ and blood - muscle fiber compartments ($T_{bl<->cyt,type,i}^{f\ or\ p}$), the tissue cells cytosolic concentrations of metabolites ($C_{cyt,type,i}$, mmol/kg w.w.) are:

$$V_{cyt,type} \frac{dC_{cyt,type,Glc}}{dt} = T_{bl<->cyt,type,Glc}^{f} - F_{HK,type}$$

$$V_{cyt,type} \frac{dC_{cyt,type,G6P}}{dt} = F_{HK,type} + F_{GP,type} - F_{MGS,type} - F_{PGI,type}$$

$$V_{cyt,type} \frac{dC_{cyt,type,F6P}}{dt} = F_{PGI,type} - F_{PFK,type}$$

$$V_{cyt,type} \frac{dC_{cyt,type,F16BP}}{dt} = F_{PFK,type} - F_{ALDO+TPI,type}$$

$$V_{cyt,type} \frac{dC_{cyt,type,GA3P}}{dt} = 2 * F_{ALDO+TPI,type} - F_{GAPDH,type} - F_{GPDH,type}$$

$$V_{cyt,type} \frac{dC_{cyt,type,13BPG}}{dt} = F_{GAPDH,type} - F_{PGK,type}$$

$$V_{cyt,type} \frac{dC_{cyt,type,PEP}}{dt} = F_{PGK,type} - F_{PK,type}$$

$$V_{cyt,type} \frac{dC_{cyt,type,Pyr}}{dt} = T_{bl<->cyt,type,Pyr}^{f} + F_{PK,type} - F_{LDH,type} - F_{ALT,type} - T_{cyt<->mit,type,Pyr}^{f}$$

$$V_{cyt,type} \frac{dC_{cyt,type,Lac}}{dt} = T_{bl<->cyt,type,Lac}^{f} + F_{LDH,type}$$

$$V_{cyt,type} \frac{dC_{cyt,type,Ala}}{dt} = T_{bl<->cyt,type,Ala}^{p} + F_{ALT,type}$$

$$V_{cyt,type} \frac{dC_{cyt,type,Gly}}{dt} = F_{MGS,type} - F_{GP,type}$$

$$V_{cyt,type} \frac{dC_{cyt,type,Gr3P}}{dt} = F_{GPDH,type} - F_{Acyl,type}$$

$$V_{cyt,type} \frac{dC_{cyt,type,Tgl}}{dt} = F_{Acyl,type} - F_{LIPA,type}$$

$$V_{cyt,type} \frac{dC_{cyt,type,Glr}}{dt} = T_{bl<->cyt,type,Glr}^{p} + F_{LIPA,type}$$

$$V_{cyt,type} \frac{dC_{cyt,type,FAC}}{dt} = F_{ACS,type} - 3 * F_{Acyl,type} - T_{cyt<->mit,type,FAC}^{f}$$

$$V_{cyt,type} \frac{dC_{cyt,type,FFA}}{dt} = T_{bl<->cyt,type,FFA}^{f} + 3 * F_{LIPA,type} - F_{ACS,type}$$

$$V_{cyt,type} \frac{dC_{cyt,type,PCr}}{dt} = -V_{cyt} \frac{dC_{cyt,Cr}}{dt} = -F_{CK,type}$$

$V_{cyt,type,CO_2} \frac{dC^F_{cyt,type,CO_2}}{dt} = T^p_{bl<->cyt,type,CO_2} - T^p_{cyt<->mit,type,CO_2}$, where superscript F denotes free dissolved CO_2

$V_{cyt,type,O_2} \frac{dC^F_{cyt,type,O_2}}{dt} = T^p_{bl<->cyt,type,O_2} - T^p_{cyt<->mit,type,O_2}$, where superscript F denotes concentration of free O_2

$$V_{cyt,type} \frac{dC_{cyt,type,AMP}}{dt} = -F_{AK,type}$$

$$V_{cyt,type} \frac{dC_{cyt,type,ATP}}{dt} = -V_{cyt,type} \frac{dC_{cyt,type,ADP}}{dt} = F_{PGK,type} + F_{PK,type} + F_{CK,type} - F_{MGS,type} - F_{HK,type} - F_{PFK,type} - 2 * F_{ACS,type} - F_{ATPase,type} - F_{AK,type} - T^f_{cyt<->mit,type,\frac{ATP}{ADP}}$$

$$V_{cyt,type} \frac{dC_{cyt,type,P_i}}{dt} = 2 * F_{MGS,type} + F_{Acyl,type} + 2 * F_{ACS,type} + F_{ATPase,type} - F_{GP,type} - F_{GAPDH,type} - T^f_{cyt<->mit,type,P_i}$$

$$V_{cyt,type} \frac{dC_{cyt,type,CoA}}{dt} = 3 * F_{Acyl,type} - F_{ACS,type} - T^f_{cyt<->mit,type,CoA}$$

$$V_{cyt,type} \frac{dC_{cyt,type,NADH}}{dt} = -V_{cyt,type} \frac{dC_{cyt,type,NAD^+}}{dt} = F_{GAPDH,type} - F_{LDH,type} - F_{GPDH,type} - T^f_{cyt<->mit,type,\frac{NADH}{NAD^+}}$$

$$V_{cyt,type} \frac{dC_{cyt,type,H^+}}{dt} = T^f_{bl<->cyt,type,H^+} - T^f_{cyt<->mit,type,H^+} + \frac{2.303}{\beta_{cyt}} * C_{cyt,type,H^+} * \big(F_{HK,type} + F_{PFK,type} + F_{GAPDH,type} + 3 * F_{LIPA,type} + 2 * F_{ACS,type} + F_{ATPase,type} - F_{PK,type} - F_{LDH,type} - F_{GPDH,type} - F_{CK,type} - T^{leak}_{cyt<->mit,type,H^+}\big)$$

where β_{cyt} = 6.65 mM/pH is buffering capacity of the cytosol for protons.

V_{cyt} indicates the volume of cytosol in kg wet weight; $V_{cyt,R} = 0.88 * V_R$ and $V_{cyt,W} = 0.92 * V_W$ are volumes of cytosol for type I and II fibers, correspondingly, where $V_R = V_W = 0.5 * V_{tis} = 2$ kg w.w., V_R – the type I fiber volume, V_W – the type II fiber volume and V_{tis} – the volume of muscle cells in the tissue. Volume distribution of muscle fibers corresponds to the human vastus lateralis muscle and used numerical values correspond to ones from the original publication (Li et al., 2012).

Table S1. The model parameter values for metabolic reactions in the Module "Cytosol" (type I fibers)

Reaction,i	$V_{max}^{f_i}$, $mmol/min$	$V_{max}^{b_i}$, $mmol/min$	$K_m^{f_i}$	$K_m^{b_i}$	$K_m^{Reg_i}$	K_{eq}^{i}	α_i, W^{-1}	τ_i, min	Reference
Glycogen Phosphorylase (GP)	2.54E-01	3.50E+06	3.06E-03	2.93E+05	1.87E-5	6.95E+00	1.51E-01*	4.0E-1	*Li et al., 2012*
Glycogen Synthase (MGS)	6.93E+01	2.36E-06	1.74E+04	1.58E+02	0	2.67E+05	3.39E-01*	2.0E+00	*Li et al., 2012*
Hexokinase (HK)	8.85E-01	6.50E-12	3.29E+00	3.93E-07	2.78E+01	1.63E+04	0	4.0E-1	*Li et al., 2012*
Phosphoglucose Isomerase (PGI)	8.48E-01	4.04E-01	2.78E-01	4.86E-02	0	3.67E-01	3.22E-01	4.0E-1	*Li et al., 2012*
Phosphofructokinase (PFK)	4.00E+00	1.27E-10	2.88E-01	9.42E-08	9.35E-02	1.03E+04	3.22E-01	4.0E-1	*Li et al., 2012*
Aldolase +triose phosphate isomerase (ALDO +TPI)	1.32E+00	1.63E-01	6.67E-01	7.90E-02	0	9.6E-01	3.22E-01	4.0E-1	*Li et al., 2012*
GA3P Dehydrogenase (GAPDH)	5.27E-01	3.39E-08	3.87E-02	1.96E-09	0	7.87E-01	3.22E-01	4.0E-1	*Li et al., 2012*
Phosphoglycerate Kinase (PGK)	2.38E-01	8.17E-03	1.58E-03	3.19E-02	0	5.88E+02	3.22E-01	4.0E-1	*Li et al., 2012*
Pyruvate Kinase (PK)	2.46E-01	6.75E-03	7.61E-09	3.13E-01	2.67E-03	1.5E+09	3.22E-01	4.0E-1	*Li et al., 2012*
Lactate Dehydrogenase (LDH)	4.88E+44	3.60E-03	1.17E+23	1.30E-15	0	1.51E+09	0	4.0E-1	*Li et al., 2012*
Alanine Aminotransferase (ALT)	9.10E-02	8.44E-04	5.28E-02	1.44E+00	0	2.94E+03	7.95E-02	4.0E-1	*Li et al., 2012*
Lipases (LIPA)	9.0E-03	2.77E-19	1.67E+01	4.52E-15	0	8.75E+00	0	4.0E-1	*Li et al., 2012*
G3P Dehydrogenase (GPDH)	1.16E-02	4.35E-04	1.96E-09	2.51E-02	0	3.41E+08	0	4.0E-1	*Li et al., 2012*
Acyltransferase (Acyl)	1.34E-02	1.31E-06	8.42E-09	2.73E-04	0	3.32E+08	0	4.0E-1	*Li et al., 2012*
Acyl-CoA Synthetase (ACS)	4.31E+00	2.29E-18	3.12E-01	6.21E-14	0	3.75E+05	3.39E-01	4.0E-1	*Li et al., 2012*
ATPase	31.51E+00	3.76E-11	5.92E+00	1.04E-05	0	1.47E+06	7.67E-02*	4.0E-3*	*Li et al., 2012*
Creatine Kinase (CK)	200	5.88E+01	2.97E-04	2.07E+01	0	2.37E+05	0	4.0E-1	*Li et al., 2012*
Adenylate Kinase (AK)	200	4.35E-04	1.32E+02	3.18E-05	0	1.11E-01	0	4.0E-1	*Li et al., 2012*

** - the value was changed during the model fitting to experimental data; K_{eq}^{i} is the equilibrium constant of the i reaction;*

Table S2. The model parameter values for metabolic reactions in the Module "Cytosol" (type II fibers)

Reaction, i	$V_{max}^{f_i}$, $mmol/min$	$V_{max}^{b_i}$, $mmol/min$	$K_m^{f_i}$	$K_m^{b_i}$	$K_m^{Reg_i}$	K_{eq}^{i}	α_i, W^{-1}	τ_i, min	Reference
Glycogen Phosphorylase (GP)	2.55E-01	3.51E+06	3.06E-03	2.93E+05	1.87E-5	6.95E+00	1.51E-01*	4.0E-1	*Li et al., 2012*
Glycogen Synthase (MGS)	3.55E+01	1.21E-06	1.74E+04	1.58E+02	0	2.67E+05	3.39E-01*	2.0E+00	*Li et al., 2012*
Hexokinase (HK)	1.34E+00	9.85E-12	3.29E+00	3.93E-07	2.78E+01	1.63E+04	0	4.0E-1	*Li et al., 2012*
Phosphoglucose Isomerase (PGI)	4.37E-01	2.10E-01	2.78E-01	4.86E-02	0	3.67E-01	3.22E-01	4.0E-1	*Li et al., 2012*
Phosphofructokinase (PFK)	3.04E+00	9.61E-11	2.88E-01	9.42E-08	9.35E-02	1.03E+04	3.22E-01	4.0E-1	*Li et al., 2012*
Aldolase +triose phosphate isomerase (ALDO +TPI)	1.35E+00	1.67E-01	6.67E-01	7.90E-02	0	9.60E-01	3.22E-01	4.0E-1	*Li et al., 2012*
GA3P Dehydrogenase (GAPDH)	6.41E-01	4.13E-08	3.87E-02	1.96E-09	0	7.87E-01	3.22E-01	4.0E-1	*Li et al., 2012*
Phosphoglycerate Kinase (PGK)	2.28E-01	7.83E-03	1.58E-03	3.19E-02	0	5.88E+02	3.22E-01	4.0E-1	*Li et al., 2012*
Pyruvate Kinase (PK)	2.37E-01	6.49E-03	7.61E-09	3.13E-01	2.67E-03	1.50E+09	3.22E-01	4.0E-1	*Li et al., 2012*
Lactate Dehydrogenase (LDH)	9.68E+44*	3.60E-03	1.17E+23	1.30E-15	0	1.51E+09	9.22E-01*	4.0E-1	*Li et al., 2012*
Alanine Aminotransferase (ALT)	1.06E-01	9.84E-04	5.28E-02	1.44E+00	0	2.94E+03	7.95E-02	4.0E-1	*Li et al., 2012*
Lipases (LIPA)	1.52E-02	4.71E-19	1.67E+01	4.52E-15	0	8.75E+00	0	4.0E-1	*Li et al., 2012*
G3P Dehydrogenase (GPDH)	1.37E-02	5.15E-04	1.96E-09	2.51E-02	0	3.41E+08	0	4.0E-1	*Li et al., 2012*
Acyltransferase (Acyl)	1.07E-02	1.05E-06	8.42E-09	2.73E-04	0	3.32E+08	0	4.0E-1	*Li et al., 2012*
Acyl-CoA Synthetase (ACS)	5.22E+00	2.77E-18	3.12E-01	6.21E-14	0	3.75E+05	3.39E-01	4.0E-1	*Li et al., 2012*
ATPase	3.16E+01	3.77E-11	5.92E+00	1.04E-05	0	1.47E+06	6.53E-02*	4.0E-3*	*Li et al., 2012*
Creatine Kinase (CK)	200	5.88E+01	2.97E-04	2.07E+01	0	2.37E+05	0	4.0E-1	*Li et al., 2012*
Adenylate Kinase (AK)	200	4.35E-04	1.32E+02	3.18E-05	0	1.11E-01	0	4.0E-1	*Li et al., 2012*

** - the value was changed during the model fitting to experimental data*

2. Rates of reactions and ODE system of the Module "Mitochondrion"

a. SBGN scheme of the module:

The Mitochondrion module consists of the 8 enzymatic reactions comprising pyruvate oxidation, citric acid (TCA) cycle, lumped β-oxidation of fatty acids and 3 reactions of the oxidative phosphorylation including electron transport chain (ETC+OxPhos in Figure S2). The list of reactions and math equations describing their rates are identical in type I and II fibers, while values of the kinetic parameters are different between two types of the fibers (See Table S3 and Table S4).

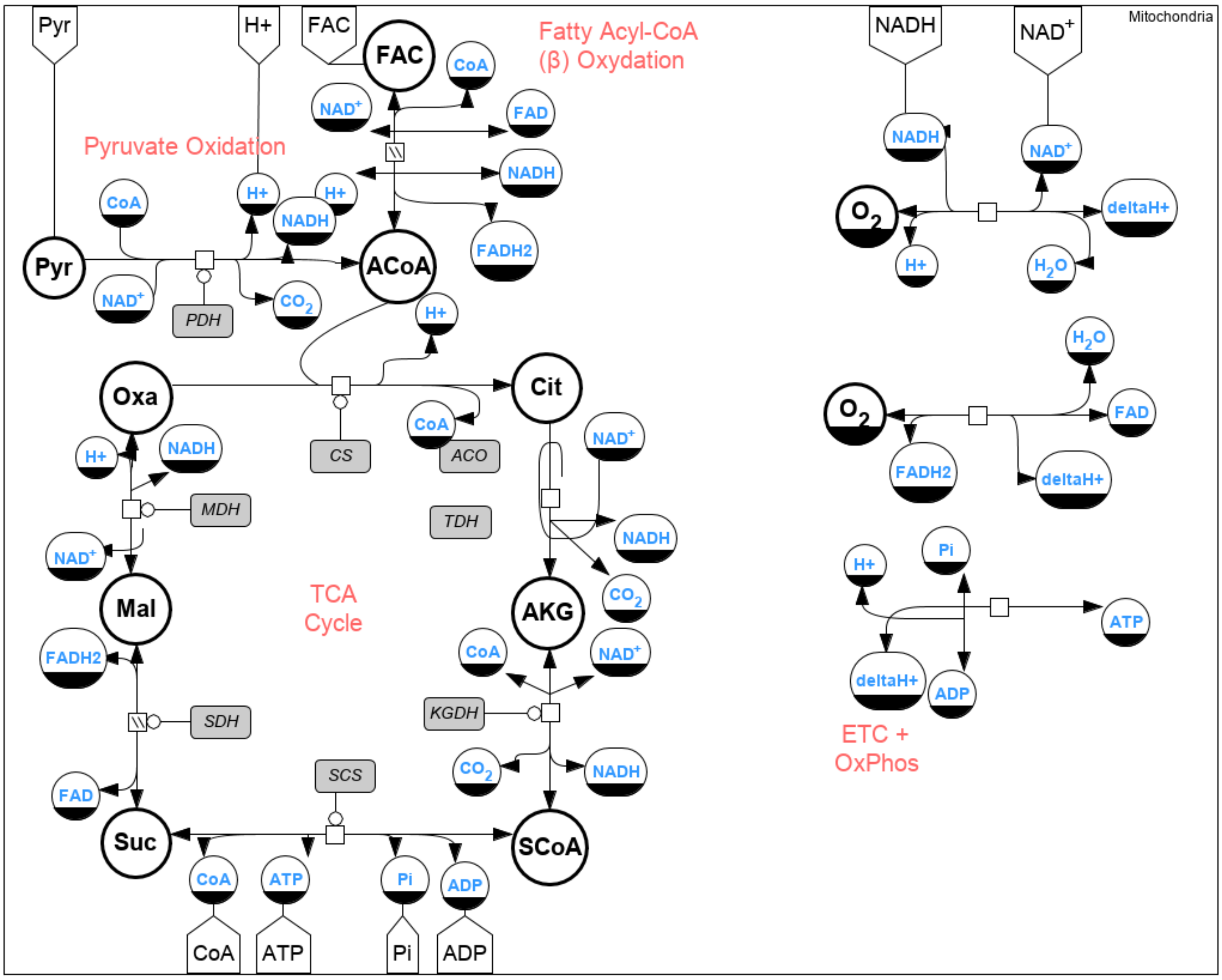

Figure S2. SBGN scheme of the metabolic reactions taking place in the Module "Mitochondrion" (vertex of the bipartite graph ⊸□ corresponds to the biochemical reaction). The diagram of metabolic pathways comprises pyruvate dehydrogenase (PDH), TCA cycle, β-oxidation and oxidative phosphorylation. Metabolites are vertices, while biochemical reactions are arcs of the diagram: Pyr, pyruvate; FAC, fatty acyl-CoA; ACoA, acetyl-CoA; CoA, coenzyme-A; Oxa, oxaloacetate; Cit, citrate; AKG, α-ketoglutarate; SCoA, succinyl-CoA; Suc, succinate; Mal, malate; FADH2, reduced flavin adenine dinucleotide; FAD, oxidized flavin adenine dinucleotide. Enzymes catalyzing biochemical reactions in the cytosol are depicted as rectangles on the diagram: PDH, pyruvate dehydrogenase; CS, citrate synthase; ACO+IDH, aconitase and isocitrate dehydrogenase; KGDH, AKG dehydrogenase; SCS, SCoA synthetase; SDH, succinate dehydrogenase; MDH, malate dehydrogenase. White arrows along the compartment border indicate contact ports for corresponding entities in the mitochondrion.

b. Antimony view which represents generated code according to the visual graph:

```
//used mathematical functions in the module
function stress(a,W,t_start,t)
1.0+a*W*(1.0-exp((t_start-t)/0.4));
end

function pw(base,power)
;
end

function adjustVb(V_f,K_b,K_f,K_eq,ph)
V_f*K_b/(K_f*K_eq)*10.0^(7.6-ph);
end

function MM(r,p,v_f,k_f,v_b,k_b,a,W,t_start,t)
(v_f/k_f*r-v_b/k_b*p)/(1.0+r/k_f+p/k_b)*stress(a,W,t_start,t);
end

function Kinetik2(r,p,v_f,k_f,v_b,k_b,c,a,W,t_start,t)
c*MM(r,p,v_f,k_f,v_b,k_b,a,W,t_start,t);
end

function Kinetik(r,p,v_f,k_f,v_b,k_b,c,k,a,W,t_start,t)
c/(k+c)*MM(r,p,v_f,k_f,v_b,k_b,a,W,t_start,t);
end

//name of the model and components
  //Function definitions
model
Li_2012_Mitochondria_R($Mitochondria.ADP,$Mitochondria.ATP,>A_19,$Mitochondr
ia.CO2,>Ca_mit,$Mitochondria.CoA,$Mitochondria.FAC,>H_Cyt,$Mitochondria.H_,>J
_leak,$Mitochondria,$Mitochondria.NADH,$Mitochondria.NAD_,$Mitochondria.O2,$
Mitochondria.Pi,$Mitochondria.Pyr,<V_CO2_mit_R,<V_O2_mit_R,>V,>W,>t_start)

//the list of used compartment in the module
  compartment Mitochondria;

//the list of used biochemical species in the module
  species ACoA in Mitochondria, ADP in Mitochondria, AKG in Mitochondria, ATP in
Mitochondria, Aconitase in Mitochondria, CO2 in Mitochondria, Cit in Mitochondria,
Citrate_Synthase in Mitochondria, CoA in Mitochondria, FAC in Mitochondria, FAD in
Mitochondria, FADH2 in Mitochondria, H2O in Mitochondria, H_ in Mitochondria,
Isocitrate_Dehydrogenase in Mitochondria, Ketoglutarate_Dehydrogenase in
Mitochondria, Mal in Mitochondria, Malate_Dehydrogenase in Mitochondria, NADH in
```

Mitochondria, NAD_ in Mitochondria, O2 in Mitochondria, Oxa in Mitochondria, Pi in Mitochondria, Pyr in Mitochondria, Pyruvate_Dehydrogenase in Mitochondria, SCoA in Mitochondria, Suc in Mitochondria, Succinate_Dehydrogenase in Mitochondria, Succinyl_CoA_Synthetase in Mitochondria, deltaH_ in Mitochondria;

//the list of used model parameters in the module
var ATP_synthesis_norm, A_19, A_20, A_21, A_22, A_23, A_24, A_25, A_26, A_27, A_28, A_29, C_IMM, Ca_mit, F, G_H, H_Cyt, J_Psi, J_leak, K_19, K_21, K_22, K_CO2hyd, K_MbO2, K_b_19, K_b_20, K_b_21, K_b_22, K_b_23, K_b_24, K_b_25, K_b_26, K_b_27, K_b_28, K_b_29, K_eq_19, K_eq_20, K_eq_21, K_eq_22, K_eq_23, K_eq_24, K_eq_25, K_eq_26, K_eq_27, K_eq_28, K_eq_29, K_f_19, K_f_20, K_f_21, K_f_22, K_f_23, K_f_24, K_f_25, K_f_26, K_f_27, K_f_28, K_f_29, Mb, Psi, R, R19_R, R20_R, R21_R, R22_R, R23_R, R24_R, R25_R, R26_R, R27_R, R28_R, R29_R, Rate_CO2_mit_R, Rate_H_mit_R, Rate_O2_mit_R, Rate_Psi_R, Ratio_H, Rel_ADP, T, V, V_CO2_mit_R, V_O2_mit_R, V_b_19, V_b_20, V_b_21, V_b_22, V_b_23, V_b_24, V_b_25, V_b_26, V_b_27, V_b_28, V_b_29, V_f_19, V_f_20, V_f_21, V_f_22, V_f_23, V_f_24, V_f_25, V_f_26, V_f_27, V_f_28, V_f_29, W, beta_mit, pHlast_mit, ph, rate_oxidation_normalized_R, t_start, unknown;

//the list of values of used model parameters in the module
//Initialization
Mitochondria = 0.4;
ACoA = 0.0166666666666667;
ADP = 8.30666666666667;
AKG = 0.108333333333333;
ATP = 7.068;
CO2 = 1.525;
Cit = 0.791666666666667;
CoA = 0.0333333333333333;
FAC = 0.00145833333333334;
FAD = 1.76666666666667;
FADH2 = 0.2;
Mal = 0.791666666666667;
NADH = 0.4975;
NAD_ = 3.15;
O2 = 0.027;
Oxa = 0.02;
Pi = 1.14583333333333;
Pyr = 0.0208333333333334;
SCoA = 1.08333333333333;
Suc = 0.791666666666667;
A_19 = 5.32468176851059;
A_20 = 0.218112023391122;
A_21 = 0.0794936353702118;
A_22 = 0.0794936353702118;

A_23 = 0.0794936353702118;
A_24 = 0.0794936353702118;
A_25 = 0.0794936353702118;
A_26 = 0.0794936353702118;
A_27 = 0.330739463658112;
A_28 = 0.330739463658112;
A_29 = 0.0275117398623704;
C_IMM = 6.75;
F = 96485.0;
G_H = 17.80458422;
K_19 = 0.361;
K_21 = 0.361;
K_22 = 0.361;
K_CO2hyd = 7.94E-4;
K_MbO2 = 308.6419753;
K_b_19 = 3.81E-7;
K_b_20 = 6.0E-6;
K_b_21 = 9.55E-7;
K_b_22 = 0.0948;
K_b_23 = 0.948;
K_b_24 = 0.33;
K_b_25 = 0.228;
K_b_26 = 3.0E-7;
K_b_27 = 3.15;
K_b_28 = 2.12;
K_b_29 = 8.68;
K_eq_19 = 1.28198016199281E7;
K_eq_20 = 2.40277252251855E185;
K_eq_21 = 5834205.78346589;
K_eq_22 = 1.70937333234441;
K_eq_23 = 7000688.52086883;
K_eq_24 = 2.44222953575007;
K_eq_25 = 16.2066336021045;
K_eq_26 = 0.129664509065361;
K_eq_27 = 9.87814193685763E40;
K_eq_28 = 1.01650487576403E27;
K_eq_29 = 1.64413750999731E-4;
K_f_19 = 0.00315;
K_f_20 = 4.67E-4;
K_f_21 = 4.8E-4;
K_f_22 = 2.99;
K_f_23 = 0.0158;
K_f_24 = 13.5;
K_f_25 = 2.01;
K_f_26 = 2.99;

K_f_27 = 2.05E-6;
K_f_28 = 0.0394;
K_f_29 = 2.71E-4;
Mb = 0.5;
Psi = 0.16;
R = 8.314;
Rel_ADP = 0.90322;
T = 310.15;
V = 2.0;
V_b_19 = 5.36524330272369E-12;
V_b_20 = 5.34432858533328E-189;
V_b_21 = 5.36524330272369E-12;
V_b_22 = 0.0329461108338452;
V_b_23 = 1.26728851241359E-5;
V_b_24 = 0.0126527359471977;
V_b_25 = 0.00996337145392688;
V_b_26 = 1.01827026763956E-6;
V_b_27 = 8.17589044237406E-4;
V_b_28 = 4.49836229612661E-7;
V_b_29 = 1.7635809142657;
V_f_19 = 0.568664744273573;
V_f_20 = 0.0999473857360283;
V_f_21 = 1.85010358968123;
V_f_22 = 1.77624934344806;
V_f_23 = 1.47864869024712;
V_f_24 = 1.26412713156653;
V_f_25 = 1.42350942235471;
V_f_26 = 1.3159340263484;
V_f_27 = 5.25597913690272E31;
V_f_28 = 8.4981473563420201E18;
V_f_29 = 9.05280349607311E-9;
beta_mit = 25.0;
ph = 7.6;

//the list of reactions in the module

//Reactions
R000154: Cit + NAD_ => AKG + CO2 + NADH; R22_R/Mitochondria;
R000155: ACoA + Oxa => Cit + CoA + H_; R21_R/Mitochondria;
R001155_mod: Citrate_Synthase-(R000155;
R000164: CoA_1_R000164 + NAD__1_R000164 + Pyr => ACoA + CO2_1_R000164 + H__1_R000164 + NADH_1_R000164; R19_R/Mitochondria;
R001164_mod: Pyruvate_Dehydrogenase-(R000164;
R20: 7CoA_1_R20 + FAC + 7FAD + 7NAD__1_R20 -> 8ACoA + 7FADH2 + 7H__1_R20 + 7NADH_1_R20; R20_R/Mitochondria;

```
  R23: AKG + CoA_1_R23 + NAD__1_R23 -> CO2_1_R23 + NADH_1_R23 + SCoA;
R23_R/Mitochondria;
  R23_mod1: Ketoglutarate_Dehydrogenase-( R23;
  R24: ADP + Pi + SCoA -> ATP + CoA_1_R24 + Suc; R24_R/Mitochondria;
  R24_mod1: Succinyl_CoA_Synthetase-( R24;
  R25: FAD_1_R25 + Suc -> FADH2_1_R25 + Mal; R25_R/Mitochondria;
  R25_mod1: Succinate_Dehydrogenase-( R25;
  R26: Mal + NAD__1_R26 -> H__1_R26 + NADH_1_R26 + Oxa;
R26_R/Mitochondria;
  R26_mod1: Malate_Dehydrogenase-( R26;
  R27: H__1_R27 + NADH_1_R27 + 0.5O2 -> H2O + NAD__1_R27 + deltaH_;
R27_R/Mitochondria;
  R28: FADH2_1_R28 + 0.5O2_1_R28 -> FAD_1_R28 + H2O_1_R28 +
deltaH__1_R28; R28_R/Mitochondria;
  R29: ADP_1_R29 + H__1_R29 + Pi_1_R29 + deltaH__1_R29 -> ATP_1_R29;
R29_R/Mitochondria;
```

//the list of used constants in the module

```
 //Constants
  const H2O, deltaH_;
```

//the list of used mathematical equations in the module

```
 //Equations
  CO2 '= Rate_CO2_mit_R/V_CO2_mit_R;
  H_ '= Rate_H_mit_R/Mitochondria;
  O2 '= Rate_O2_mit_R/V_O2_mit_R;
  Psi '= Rate_Psi_R/C_IMM;

  H_ = 10.0^(-ph+3.0);
  H_Cyt = 10.0^(-7.1+3.0);

  ATP_synthesis_norm := R29_R/Mitochondria;
  G_H := F*Psi+R*T*ln(H_Cyt/H_);
  Mitochondria := 0.12*V;
  R19_R :=
Kinetik(CoA*NAD_*Pyr,ACoA*CO2*H_*NADH,V_f_19,K_f_19,V_b_19,K_b_19,Rel
_ADP,K_19,A_19,W,t_start,time);
  R20_R :=
MM(CoA*FAC*FAD*NAD_,ACoA*FADH2*H_*NADH,V_f_20,K_f_20,V_b_20,K_b
_20,A_20,W,t_start,time);
  R21_R :=
Kinetik(ACoA*Oxa,Cit*CoA*H_,V_f_21,K_f_21,V_b_21,K_b_21,Rel_ADP,K_21,A_2
1,W,t_start,time);
```

```
  R22_R :=
Kinetik(Cit*NAD_,AKG*CO2*NADH,V_f_22,K_f_22,V_b_22,K_b_22,Rel_ADP,K_2
2,A_22,W,t_start,time);
  R23_R :=
MM(AKG*CoA*NAD_,CO2*NADH*SCoA,V_f_23,K_f_23,V_b_23,K_b_23,A_23,W,
t_start,time);
  R24_R :=
MM(ADP*Pi*SCoA,ATP*CoA*Suc,V_f_24,K_f_24,V_b_24,K_b_24,A_24,W,t_start,ti
me);
  R25_R :=
MM(FAD*Suc,FADH2*Mal,V_f_25,K_f_25,V_b_25,K_b_25,A_25,W,t_start,time);
  R26_R :=
MM(Mal*NAD_,H_*NADH*Oxa,V_f_26,K_f_26,V_b_26,K_b_26,A_26,W,t_start,time
);
  R27_R := MM(H_*NADH*pw(O2,0.5),NAD_,exp(-
10.0*G_H/(R*T))*V_f_27,K_f_27,V_b_27,K_b_27,A_27,W,t_start,time);
  R28_R := MM(FADH2*pw(O2,0.5),FAD,exp(-
6.0*G_H/(R*T))*V_f_28,K_f_28,V_b_28,K_b_28,A_28,W,t_start,time);
  R29_R :=
MM(ADP*H_*Pi,ATP,exp(3.0*G_H/(R*T))*V_f_29,K_f_29,V_b_29,K_b_29,A_29,W,
t_start,time);
  Rate_CO2_mit_R := R23_R+R19_R+R22_R;
  Rate_H_mit_R :=
2.303/beta_mit*H_*(R19_R+7.0*R20_R+R21_R+R26_R+J_leak+3.0*R29_R-R27_R-
R29_R-10.0*R27_R-6.0*R28_R);
  Rate_O2_mit_R := -0.5*(R28_R+R27_R);
  Rate_Psi_R := 10.0*R27_R+6.0*R28_R-3.0*R29_R-J_leak;
  Ratio_H := ln(H_Cyt/H_);
  Rel_ADP := ADP/ATP;
  V_CO2_mit_R := Mitochondria*(1.0+K_CO2hyd/10.0^(-7.1+3.0));
  V_O2_mit_R := Mitochondria*(1.0+Mb*K_MbO2/(1.0+K_MbO2*O2)^2.0);
  V_b_19 := adjustVb(V_f_19,K_b_19,K_f_19,K_eq_19,pHlast_mit);
  V_b_20 := adjustVb(V_f_20,K_b_20,K_f_20,K_eq_20,pHlast_mit);
  V_b_21 := adjustVb(V_f_21,K_b_21,K_f_21,K_eq_21,pHlast_mit);
  V_b_22 := adjustVb(V_f_22,K_b_22,K_f_22,K_eq_22,pHlast_mit);
  V_b_23 := adjustVb(V_f_23,K_b_23,K_f_23,K_eq_23,pHlast_mit);
  V_b_24 := adjustVb(V_f_24,K_b_24,K_f_24,K_eq_24,pHlast_mit);
  V_b_25 := adjustVb(V_f_25,K_b_25,K_f_25,K_eq_25,pHlast_mit);
  V_b_26 := adjustVb(V_f_26,K_b_26,K_f_26,K_eq_26,pHlast_mit);
  V_b_27 := adjustVb(V_f_27,K_b_27,K_f_27,K_eq_27,pHlast_mit);
  V_b_28 := adjustVb(V_f_28,K_b_28,K_f_28,K_eq_28,pHlast_mit);
  V_b_29 := adjustVb(V_f_29,K_b_29,K_f_29,K_eq_29,pHlast_mit);
  pHlast_mit := 3.0-log(H_,10.0);
  rate_oxidation_normalized_R := R20_R/Mitochondria;
```

//the list of used model species and their alias

```
 //Titles
  ADP_1_R29 is "ADP";
  ATP_1_R29 is "ATP";
  Aconitase is "ACO";
  CO2 is "CO<sub>2";
  CO2_1_R000164 is "CO<sub>2";
  CO2_1_R23 is "CO<sub>2";
  Citrate_Synthase is "CS";
  CoA_1_R000164 is "CoA";
  CoA_1_R20 is "CoA";
  CoA_1_R23 is "CoA";
  CoA_1_R24 is "CoA";
  FADH2_1_R25 is "FADH2";
  FADH2_1_R28 is "FADH2";
  FAD_1_R25 is "FAD";
  FAD_1_R28 is "FAD";
  H2O is "H<sub>2</sub>O";
  H2O_1_R28 is "H<sub>2</sub>O";
  H_ is "H+";
  H__1_R000164 is "H+";
  H__1_R20 is "H+";
  H__1_R26 is "H+";
  H__1_R27 is "H+";
  H__1_R29 is "H+";
  Isocitrate_Dehydrogenase is "TDH";
  Ketoglutarate_Dehydrogenase is "KGDH";
  Malate_Dehydrogenase is "MDH";
  NADH_1_R000164 is "NADH";
  NADH_1_R20 is "NADH";
  NADH_1_R23 is "NADH";
  NADH_1_R26 is "NADH";
  NADH_1_R27 is "NADH";
  NAD_ is "NAD<sup>+";
  NAD__1_R000164 is "NAD<sup>+";
  NAD__1_R20 is "NAD<sup>+";
  NAD__1_R23 is "NAD<sup>+";
  NAD__1_R26 is "NAD<sup>+";
  NAD__1_R27 is "NAD<sup>+";
  O2 is "O<sub>2</sub>";
  O2_1_R28 is "O<sub>2</sub>";
  Pi_1_R29 is "Pi";
  Pyruvate_Dehydrogenase is "PDH";
  Succinate_Dehydrogenase is "SDH";
  Succinyl_CoA_Synthetase is "SCS";
```

```
deltaH_ is "deltaH+";
deltaH__1_R28 is "deltaH+";
deltaH__1_R29 is "deltaH+";

//SBGN Properties
@ACoA.sbgnType = "simple chemical";
@ADP.sbgnType = "simple chemical";
@AKG.sbgnType = "simple chemical";
@ATP.sbgnType = "simple chemical";
@Aconitase.sbgnType = "macromolecule";
@CO2.sbgnType = "simple chemical";
@Cit.sbgnType = "simple chemical";
@Citrate_Synthase.sbgnType = "macromolecule";
@CoA.sbgnType = "simple chemical";
@FAC.sbgnType = "simple chemical";
@FAD.sbgnType = "simple chemical";
@FADH2.sbgnType = "simple chemical";
@H2O.sbgnType = "simple chemical";
@H_.sbgnType = "simple chemical";
@Isocitrate_Dehydrogenase.sbgnType = "macromolecule";
@Ketoglutarate_Dehydrogenase.sbgnType = "macromolecule";
@Mal.sbgnType = "simple chemical";
@Malate_Dehydrogenase.sbgnType = "macromolecule";
@NADH.sbgnType = "simple chemical";
@NAD_.sbgnType = "simple chemical";
@O2.sbgnType = "simple chemical";
@Oxa.sbgnType = "simple chemical";
@Pi.sbgnType = "simple chemical";
@Pyr.sbgnType = "simple chemical";
@Pyruvate_Dehydrogenase.sbgnType = "macromolecule";
@SCoA.sbgnType = "simple chemical";
@Suc.sbgnType = "simple chemical";
@Succinate_Dehydrogenase.sbgnType = "macromolecule";
@Succinyl_CoA_Synthetase.sbgnType = "macromolecule";
@deltaH_.sbgnType = "simple chemical";
end
```

c. Mathematical equations of the metabolic reactions in the Module "Mitochondrion":

The biochemical reaction flux expressions and used kinetic laws of the reactions in the Module "Mitochondrion" are originated from Li et al., 2012. According to the original paper, the flux expressions (F_i) for enzymatic reactions include the next set of parameters: $V_{max}^{f_i}$ and $V_{max}^{b_i}$ are the maximal forward and backward *i*- reaction velocities, respectively, while $K_m^{f_i}$ and $K_m^{b_i}$ are the corresponding Michaelis-Menten constants. Some flux expressions ($F_{PDH}, F_{CS}, F_{ACO+IDH}$) in the

Module account for activation and/or inhibition of the metabolic reaction flux by intermediate metabolites in the mitochondrial metabolism, where C_i is the metabolite's concentration and $K_m^{Reg_i}$ is the activation or inhibition constant of the regulatory metabolite in the *i* reaction. The rate of each metabolic or oxidative phosphorylation reaction of the module is multiplied by a linear function of the work rate (W) to consider activation mechanisms related to an exercises response in a similar way to cytosolic compartment. Thus, flux expressions for the metabolic reactions in the Module "Mitochondrion" represents by the next set of equations:

R19. Pyruvate Dehydrogenase (as PDH is shown in Figure S2)

$$F_{PDH} = \left(\frac{\frac{C_{ADP}}{C_{ATP}}}{K_m^{Reg_{PDH}} + \frac{C_{ADP}}{C_{ATP}}}\right) * \left(\frac{\frac{V_{max}^{f_{PDH}}}{K_m^{f_{PDH}}} * C_{Pyr} * C_{CoA} * C_{NAD^+} - \frac{V_{max}^{b_{PDH}}}{K_m^{b_{PDH}}} * C_{ACoA} * C_{CO_2} * C_{NADH} * C_{H^+}}{1 + \frac{C_{Pyr} * C_{CoA} * C_{NAD^+}}{K_m^{f_{PDH}}} + \frac{C_{ACoA} * C_{CO_2} * C_{NADH} * C_{H^+}}{K_m^{b_{PDH}}}}\right) *$$

$$* \left(1 + \alpha_{PDH} * W * \left(1 - e^{\frac{t_{start} - t}{\tau_{PDH}}}\right)\right)$$

R20. β-oxidation of the fatty Acyl-CoA

$$F_{\beta oxi} = \left(\frac{\frac{V_{max}^{f_{\beta oxi}}}{K_m^{f_{\beta oxi}}} * C_{FAC} * C_{CoA} * C_{NAD^+} * C_{FAD} - \frac{V_{max}^{b_{\beta oxi}}}{K_m^{b_{\beta oxi}}} * C_{ACoA} * C_{NADH} * C_{H^+} * C_{FADH_2}}{1 + \frac{C_{FAC} * C_{CoA} * C_{NAD^+} * C_{FAD}}{K_m^{f_{\beta oxi}}} + \frac{C_{ACoA} * C_{NADH} * C_{FADH_2} * C_{H^+}}{K_m^{b_{\beta oxi}}}}\right) *$$

$$* \left(1 + \alpha_{\beta oxi} * W * \left(1 - e^{\frac{t_{start} - t}{\tau_{\beta oxi}}}\right)\right)$$

R21. Citrate Synthase (as CS is shown in Figure S2)

$$F_{CS} = \left(\frac{\frac{C_{ADP}}{C_{ATP}}}{K_m^{Reg_{CS}} + \frac{C_{ADP}}{C_{ATP}}}\right) * \left(\frac{\frac{V_{max}^{f_{CS}}}{K_m^{f_{CS}}} * C_{ACoA} * C_{Oxa} - \frac{V_{max}^{b_{CS}}}{K_m^{b_{CS}}} * C_{Cit} * C_{CoA} * C_{H^+}}{1 + \frac{C_{ACoA} * C_{Oxa}}{K_m^{f_{CS}}} + \frac{C_{Cit} * C_{CoA} * C_{H^+}}{K_m^{b_{CS}}}}\right) * \left(1 + \alpha_{CS} * W * \left(1 - e^{\frac{t_{start} - t}{\tau_{CS}}}\right)\right)$$

R22. Aconitase + Isocitrate Dehydrogenase (as ACO+IDH is shown in Figure S2)

$$F_{ACO+IDH} = \left(\frac{\frac{C_{ADP}}{C_{ATP}}}{K_m^{Reg_{ACO+IDH}} + \frac{C_{ADP}}{C_{ATP}}}\right) * \left(\frac{\frac{V_{max}^{f_{ACO+IDH}}}{K_m^{f_{ACO+IDH}}} * C_{Cit} * C_{NAD^+} - \frac{V_{max}^{b_{ACO+IDH}}}{K_m^{b_{ACO+IDH}}} * C_{AKG} * C_{CO_2} * C_{NADH}}{1 + \frac{C_{Cit} * C_{NAD^+}}{K_m^{f_{ACO+IDH}}} + \frac{C_{AKG} * C_{CO_2} * C_{NADH}}{K_m^{b_{ACO+IDH}}}}\right) *$$

$$* \left(1 + \alpha_{ACO+IDH} * W * \left(1 - e^{\frac{t_{start} - t}{\tau_{ACO+IDH}}}\right)\right)$$

R23. α-Ketoglutarate Dehydrogenase (as KGDH is shown in Figure S2)

$$F_{KGDH} = \left(\frac{\frac{V_{max}^{f_{KGDH}}}{K_m^{f_{KGDH}}} * C_{AKG} * C_{CoA} * C_{NAD^+} - \frac{V_{max}^{b_{KGDH}}}{K_m^{b_{KGDH}}} * C_{SCoA} * C_{CO_2} * C_{NADH}}{1 + \frac{C_{AKG} * C_{CoA} * C_{NAD^+}}{K_m^{f_{KGDH}}} + \frac{C_{SCoA} * C_{CO_2} * C_{NADH}}{K_m^{b_{KGDH}}}}\right) * \left(1 + \alpha_{KGDH} * W * \left(1 - e^{\frac{t_{start} - t}{\tau_{KGDH}}}\right)\right)$$

R24. Succinyl-CoA Synthetase (as SCS is shown in Figure S2)

$$F_{SCS} = \left(\frac{\frac{V_{max}^{fscs}}{K_m^{fscs}} * C_{SCoA} * C_{ADP} * C_{P_i} - \frac{V_{max}^{bscs}}{K_m^{bscs}} * C_{Suc} * C_{CoA} * C_{ATP}}{1 + \frac{C_{SCoA} * C_{ADP} * C_{P_i}}{K_m^{fscs}} + \frac{C_{Suc} * C_{CoA} * C_{ATP}}{K_m^{bscs}}} \right) * (1 + \alpha_{SCS} * W * (1 - e^{\frac{t_{start} - t}{\tau_{SCS}}}))$$

R25. Succinate Dehydrogenase (as SDH is shown in Figure S2)

$$F_{SDH} = \left(\frac{\frac{V_{max}^{fSDH}}{K_m^{fSDH}} * C_{Suc} * C_{FAD} - \frac{V_{max}^{bSDH}}{K_m^{bSDH}} * C_{Mal} * C_{FADH_2}}{1 + \frac{C_{Suc} * C_{FAD}}{K_m^{fSDH}} + \frac{C_{Mal} * C_{FADH_2}}{K_m^{bSDH}}} \right) * (1 + \alpha_{SDH} * W * (1 - e^{\frac{t_{start} - t}{\tau_{SDH}}}))$$

R26. Malate Dehydrogenase (as MDH is shown in Figure S2)

$$F_{MDH} = \left(\frac{\frac{V_{max}^{fMDH}}{K_m^{fMDH}} * C_{Mal} * C_{NAD^+} - \frac{V_{max}^{bMDH}}{K_m^{bMDH}} * C_{Oxa} * C_{NADH} * C_{H^+}}{1 + \frac{C_{Mal} * C_{NAD^+}}{K_m^{fMDH}} + \frac{C_{Oxa} * C_{NADH} * C_{H^+}}{K_m^{bMDH}}} \right) * (1 + \alpha_{MDH} * W * (1 - e^{\frac{t_{start} - t}{\tau_{MDH}}}))$$

R27. Complex I+III+IV (NADH used as a reduced compound)

$$F_{OP_{NADH}} = \left(\frac{e^{\left(-\frac{10\Delta G_{H^+}}{RT}\right)} * \frac{V_{max}^{fOP_{NADH}}}{K_m^{fOP_{NADH}}} * C_{NADH} * C_{O_2}^{0.5} * C_{H^+} - \frac{V_{max}^{bOP_{NADH}}}{K_m^{bOP_{FNADH}}} * C_{NAD^+}}{1 + \frac{C_{NADH} * C_{O_2}^{0.5} * C_{H^+}}{K_m^{fOP_{NADH}}} + \frac{C_{NAD^+}}{K_m^{bOP_{NADH}}}} \right) * (1 + \alpha_{OP_{NADH}} * W * (1 - e^{\frac{t_{start} - t}{\tau_{OP_{NADH}}}}))$$

R28. Complex II+III+IV ($FADH_2$ used as a reduced compound)

$$F_{OP_{FADH_2}} = \left(\frac{e^{\left(-\frac{6\Delta G_{H^+}}{RT}\right)} * \frac{V_{max}^{fOP_{FADH_2}}}{K_m^{fOP_{FADH_2}}} * C_{FADH_2} * C_{O_2}^{0.5} - \frac{V_{max}^{bOP_{FADH_2}}}{K_m^{bOP_{FADH_2}}} * C_{FAD}}{1 + \frac{C_{FADH_2} * C_{O_2}^{0.5}}{K_m^{fOP_{FADH_2}}} + \frac{C_{FAD}}{K_m^{bOP_{FADH_2}}}} \right) * (1 + \alpha_{OP_{FADH_2}} * W * (1 - e^{\frac{t_{start} - t}{\tau_{OP_{FADH_2}}}}))$$

R29. ATP synthesis via F_1F_0-ATP synthase complex or Complex V

$$F_{ATPase} = \left(\frac{e^{\left(\frac{3\Delta G_{H^+}}{RT}\right)} * \frac{V_{max}^{fATPase}}{K_m^{fATPase}} * C_{ADP} * C_{P_i} * C_{H^+} - \frac{V_{max}^{bATPase}}{K_m^{bATPase}} * C_{ATP}}{1 + \frac{C_{ADP} * C_{P_i} * C_{H^+}}{K_m^{fATPase}} + \frac{C_{ATP}}{K_m^{bATPase}}} \right) * (1 + \alpha_{ATPase} * W * (1 - e^{\frac{t_{start} - t}{\tau_{ATPase}}}))$$

According to the above-mentioned rate equations and transport fluxes both between cytosol-mitochondria ($T_{cyt<->mit,i}^{f\ or\ p}$, where superscripts f and p denote facilitated and passive transports, respectively), $type \in (type\ I\ fiber\ (R), type\ II\ fiber (W))$ and blood - muscle fiber compartments ($T_{bl<->cyt,type,i}^{f\ or\ p}$), the tissue cells mitochondrial (V_{mit} indicates the volume of mitochondria in kg wet weight; $V_{mit,R} = 0.12 * V_R$ and $V_{mit,W} = 0.08 * V_W$ are volumes of mitochondria for type I and II fibers, correspondingly, where $V_R = V_w = 0.5 * V_{tis} = 2$ kg w.w., V_R – the type I fiber

volume, V_W – the type II fiber volume and V_{tis} – the volume of muscle cells in the tissue) concentrations of metabolites ($C_{mit,type,i}$) are:

$$V_{mit,type}\frac{dC_{mit,type,Pyr}}{dt} = T^{f}_{cyt<->mit,type,Pyr} - F_{PDH,type}$$

$$V_{mit,type}\frac{dC_{mit,type,ACoA}}{dt} = F_{PDH,type} + 8 * F_{\beta oxi,type} - F_{CS,type}$$

$$V_{mit,type}\frac{dC_{mit,type,CoA}}{dt} = T^{f}_{cyt<->mit,type,CoA} + F_{CS,type} + F_{SCS,type} - F_{PDH,type} - 7 * F_{\beta oxi,type} - F_{KGDH,type}$$

$$V_{mit,type}\frac{dC_{mit,type,FAC}}{dt} = T^{f}_{cyt<->mit,type,FAC} - F_{\beta oxi,type}$$

$$V_{mit,type}\frac{dC_{mit,type,Oxa}}{dt} = F_{MDH,type} - F_{CS,type}$$

$$V_{mit,type}\frac{dC_{mit,type,Cit}}{dt} = F_{CS,type} - F_{ACO+IDH,type}$$

$$V_{mit,type}\frac{dC_{mit,type,AKG}}{dt} = F_{ACO+IDH,type} - F_{KGDH,type}$$

$$V_{mit,type}\frac{dC_{mit,type,SCoA}}{dt} = F_{KGDH,type} - F_{SCS,type}$$

$$V_{mit,type}\frac{dC_{mit,type,Suc}}{dt} = F_{SCS,type} - F_{SDH,type}$$

$$V_{mit,type}\frac{dC_{mit,type,Mal}}{dt} = F_{SDH,type} - F_{MDH,type}$$

$$V_{mit,type,CO_2}\frac{dC^{F}_{mit,type,CO_2}}{dt} = T^{p}_{cyt<->mit,type,CO_2} + F_{PDH,type} + F_{ACO+IDH,type} + F_{KGDH,type}$$

$$V_{mit,type,O_2}\frac{dC^{F}_{mit,type,O_2}}{dt} = T^{p}_{cyt<->mit,type,O_2} - \frac{1}{2} * F_{OP_{NADH,type}} - \frac{1}{2} * F_{OP_{FADH_2,type}}$$

$$V_{mit,type}\frac{dC_{mit,type,P_i}}{dt} = T^{f}_{cyt<->mit,type,P_i} - F_{SCS,type} - F_{ATPase,type}$$

$$V_{mit,type}\frac{dC_{mit,type,ATP}}{dt} = -V_{mit,type}\frac{dC_{mit,type,ADP}}{dt} = T^{f}_{cyt<->mit,type,\frac{ATP}{ADP}} + F_{SCS,type} + F_{ATPase,type}$$

$$V_{mit,type}\frac{dC_{mit,type,NADH}}{dt} = -V_{mit,type}\frac{dC_{mit,type,NAD^+}}{dt} = T^{f}_{cyt<->mit,type,\frac{NADH}{NAD^+}} + F_{PDH,type} + 7 * F_{\beta oxi,type} + F_{ACO+IDH,type} + F_{KGDH,type} + F_{MDH,type} - F_{OP_{NADH,type}}$$

$$V_{mit,type}\frac{dC_{mit,type,FADH_2}}{dt} = -V_{mit,type}\frac{dC_{mit,type,FAD}}{dt} = 7 * F_{\beta oxi,type} + F_{SDH,type} - F_{OP_{FADH_2},type}$$

$$V_{mit,type}\frac{dC_{mit,type,H^+}}{dt} = T^{f}_{cyt<->mit,type,H^+} + \frac{2.303}{\beta_{mit}} * C_{mit,type,H^+} * \Big(T^{leak}_{cyt<->mit,type,H^+} + F_{PDH,type} + 7 * F_{\beta oxi,type} + F_{CS,type} + F_{MDH,type} - F_{PK,type} - 6 * F_{OP_{FADH_2,type}} - (10+1) * F_{OP_{NADH,type}} - (3-1) * F_{ATPase,type}\Big)$$

where β_{mit} = 25 mM/pH is buffering capacity of mitochondria for protons;

$$C_{IMM}\frac{d\Delta\Psi_{type}}{dt} = 10 * F_{OP_{NADH,type}} + 6 * F_{OP_{FADH_2,type}} - 3 * F_{ATPase,type} - T^{leak}_{cyt<\to mit,type,H^+}$$

where $\Delta\Psi$ is the mitochondrial membrane potential and C_{IMM} = 6.75*10^{-3} mmol/mV is the capacity of the inner mitochondrial membrane.

Table S3. The model parameter values for metabolic reactions in the Module "Mitochondrion" (type I fibers)

Reaction, i	$V_{max}^{f_i}$, $mmol/min$	$V_{max}^{b_i}$, $mmol/min$	$K_m^{f_i}$	$K_m^{b_i}$	$K_m^{Reg_i}$	K_{eq}^i	α_i, W^{-1}	τ_i, min	Reference
Pyruvate Dehydrogenase (PDH)	5.69E-01	5.37E-12	3.15E-03	3.81E-07	3.61E-1	1.28E+07	5.32E+00*	4.0E-1	*Li et al., 2012*
β-oxidation (βoxi)	1.00E-01	5.34E-189	4.67E-04	6.00E-06	0	2.4E+185	2.18E-01	4.0E-1	*Li et al., 2012*
Citrate Synthase (CS)	1.85E+00	5.37E-12	4.80E-04	9.55E-07	3.61E-1	5.83E+06	7.94E-02	4.0E-1	*Li et al., 2012*
Aconitase + Isocitrate Dehydrogenase (ACO+IDH)	1.78E+00	3.30E-02	2.99E+00	9.48E-02	3.61E-1	1.71E+00	7.94E-02	4.0E-1	*Li et al., 2012*
AKG Dehydrogenase (KGDH)	1.48E+00	1.27E-05	1.58E-02	9.48E-01	0	7.00E+06	7.94E-02	4.0E-1	*Li et al., 2012*
Succinyl-CoA Synthetase (SCS)	1.26E+00	1.27E-02	1.35E+01	3.30E-01	0	2.44E+00	7.94E-02	4.0E-1	*Li et al., 2012*
Succinate Dehydrogenase (SDH)	1.42E+00	1.00E-02	2.01E+00	2.28E-01	0	1.62E+01	7.94E-02	4.0E-1	*Li et al., 2012*
Malate Dehydrogenase (MDH)	1.32E+00	1.02E-06	2.99E+00	3.00E-07	0	1.30E-01	7.94E-02	4.0E-1	*Li et al., 2012*
Complex I+III+IV (OP_{NADH})	5.26E+31	8.18E-04	2.05E-06	3.15E+00	0	9.88E+40	3.31E-01	4.0E-1	*Li et al., 2012*
Complex II+III+IV (OP_{FADH_2})	8.50E+18	4.50E-07	3.94E-02	2.12E+00	0	1.02E+27	3.31E-01	4.0E-1	*Li et al., 2012*
ATP synthase ($ATPase$)	9.05E-09	1.76E+00	2.71E-04	8.68E+00	0	1.64E-04	2.75E-02	4.0E-1	*Li et al., 2012*

** - the value was changed during the model fitting to experimental data;* K_{eq}^i *is the equilibrium constant of the i reaction;*

Table S4. The model parameter values for metabolic reactions in the Module "Mitochondrion" (type II fibers)

Reaction, i	$V_{max}^{f_i}$, $mmol/min$	$V_{max}^{b_i}$, $mmol/min$	$K_m^{f_i}$	$K_m^{b_i}$	$K_m^{Reg_i}$	K_{eq}^i	α_i, W^{-1}	τ_i, min	Reference
Pyruvate Dehydrogenase (PDH)	3.99E-01	3.76E-12	3.15E-03	3.81E-07	3.61E-1	1.28E+07	8.32E+00*	4.0E-1	*Li et al., 2012*
β-oxidation (βoxi)	5.55E-02	2.97E-189	4.67E-04	6.00E-06	0	2.4E+185	2.18E-01	4.0E-1	*Li et al., 2012*
Citrate Synthase (CS)	1.49E+00	5.07E-10	4.80E-04	9.55E-07	3.61E-1	5.83E+06	7.94E-02	4.0E-1	*Li et al., 2012*
Aconitase + Isocitrate Dehydrogenase (ACO+IDH)	1.63E+00	3.02E-02	2.99E+00	9.48E-02	3.61E-1	1.71E+00	7.94E-02	4.0E-1	*Li et al., 2012*
AKG Dehydrogenase (KGDH)	9.96E-01	8.54E-06	1.58E-02	9.48E-01	0	7.00E+06	7.94E-02	4.0E-1	*Li et al., 2012*
Succinyl-CoA Synthetase (SCS)	1.34E+00	1.34E-02	1.35E+01	3.30E-01	0	2.44E+00	7.94E-02	4.0E-1	*Li et al., 2012*
Succinate Dehydrogenase (SDH)	1.09E+00	7.65E-03	2.01E+00	2.28E-01	0	1.62E+01	7.94E-02	4.0E-1	*Li et al., 2012*
Malate Dehydrogenase (MDH)	1.15E+00	8.91E-07	2.99E+00	3.00E-07	0	1.30E-01	7.94E-02	4.0E-1	*Li et al., 2012*
Complex I+III+IV (OP_{NADH})	5.26E+31	8.18E-04	2.05E-06	3.15E+00	0	9.88E+40	4.29E-03*	4.0E-1	*Li et al., 2012*
Complex II+III+IV (OP_{FADH_2})	7.44E+18	3.94E-07	3.94E-02	2.12E+00	0	1.02E+27	4.29E-03*	4.0E-1	*Li et al., 2012*
ATP synthase ($ATPase$)	1.01E-08	1.97E+00	2.71E-04	8.68E+00	0	1.64E-04	2.75E-02	4.0E-1	*Li et al., 2012*

** - the value was changed during the model fitting to experimental data;* K_{eq}^i *is the equilibrium constant of the i reaction;*

3. Rates of reactions and ODE system of the Module "Transport: Cytosol-Mitochondria"

a. SBGN scheme of the module:

The "Transport: Cytosol-Mitochondria" module (Figure S3) consists of the 9 transport reactions which represent facilitated transport of glucose, pyruvate, FAC, CoA, P_i and protons (H^+), passive transport reactions for CO_2 and O_2 and two exchange or effective transport fluxes for pairs: redox state ($RS = \frac{NADH}{NAD^+}$) and phosphorylation state ($PS = \frac{ATP}{ADP}$) across the inner membrane of mitochondria using phenomenological equations (see below). The list of reactions and math equations describing their rates are identical in type I and II fibers, while values of the kinetic parameters are different between two types of the fibers (See Table S5 and Table S6).

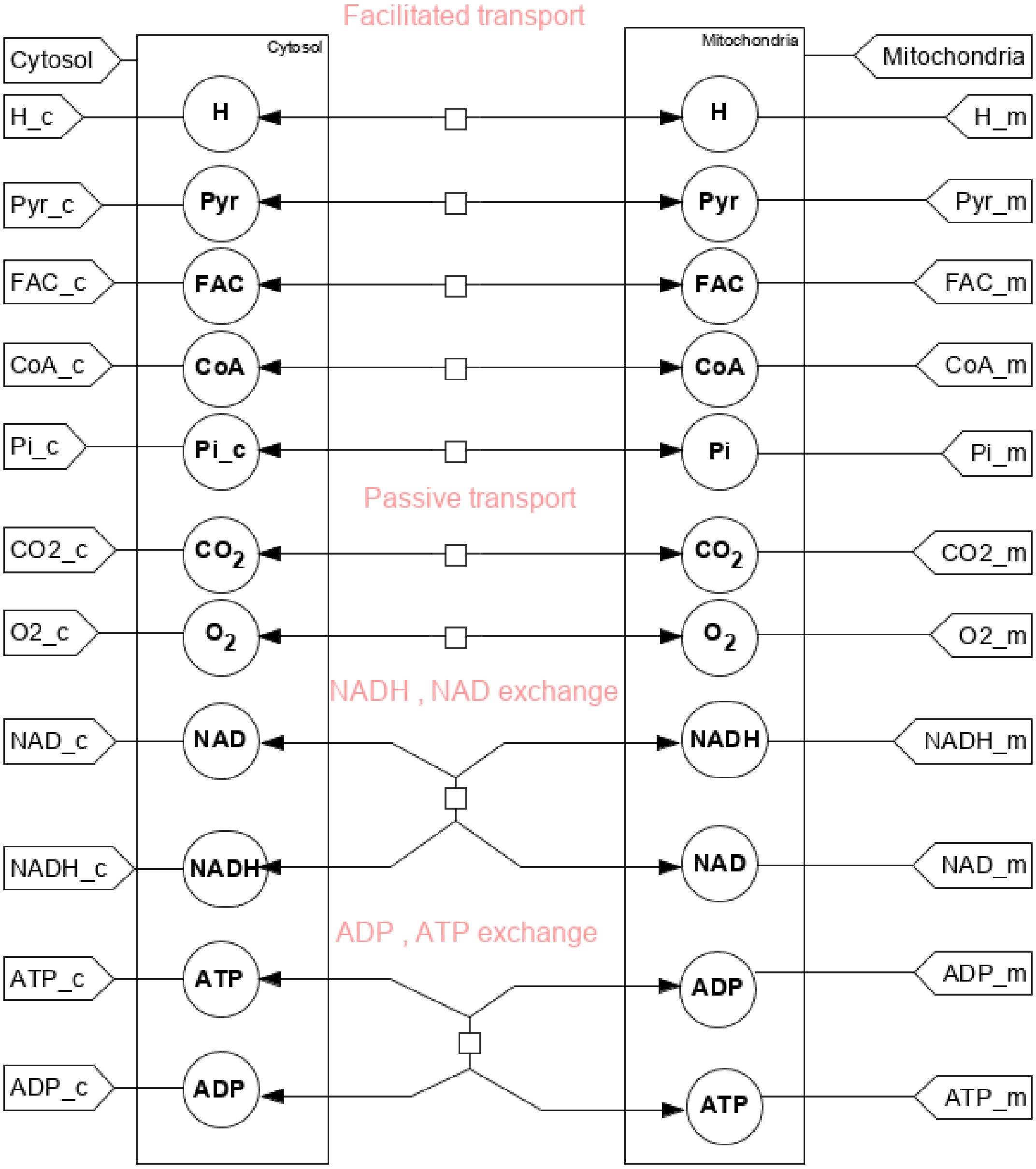

Figure S3. SBGN scheme of the transport reactions (vertex of the bipartite graph ⊣□⊢ corresponds to the reaction) taking place in the Module "Transport: Cytosol-Mitochondria". All abbreviations and aliases of metabolites correspond to the above-mentioned descriptions. Specification for cytosol and mitochondria

compartmental affiliation of a certain metabolite designated by "_c" and "_m", respectively. White arrows along the compartment border indicate contact ports for corresponding entities in the cytosol or in the mitochondrion.

b. Antimony view which represents generated code according to the visual graph:

//used mathematical functions in the module

```
function tissue_conc(cyt,mit,Cytosol,Mitochondria)
(cyt*Cytosol+mit*Mitochondria)/(Cytosol+Mitochondria);
end

function stress(a,W,t_start,t)
1.0+a*W*(1.0-exp((t_start-t)/0.4));
end

function Transport(T,M,C_1,C_2,a,W,t_start,t)
T*(C_1/(M+C_1)-C_2/(M+C_2))*stress(a,W,t_start,t);
end

function Exchange(T,M_1,M_2,S_1,S_2,a,W,t_start,t)
T*(S_1/(M_1+S_1)-S_2/(M_2+S_2))*stress(a,W,t_start,t);
end
```

//name of the model and components

```
  //Function definitions
model
Cytosol_Mitochondria_Transport_R($Cytosol.ADP_c,$Mitochondria.ADP_m,$Cytosol.ATP_c,$Mitochondria.ATP_m,$Cytosol.CO2_c,$Mitochondria.CO2_m,$Cytosol.CoA_c,$Mitochondria.CoA_m,$Cytosol,$Cytosol.FAC_c,$Mitochondria.FAC_m,$Cytosol.H_c,$Mitochondria.H_m,<J_leak_R,$Mitochondria,$Cytosol.NADH_c,$Mitochondria.NADH_m,$Cytosol.NAD_c,$Mitochondria.NAD_m,$Cytosol.O2_c,$Mitochondria.O2_m,$Cytosol.Pi_c,$Mitochondria.Pi_m,$Cytosol.Pyr_c,$Mitochondria.Pyr_m,<Pyr_tis_R,>V_CO2_c,>V_CO2_m,>V_O2_c,>V_O2_m,>V,>W,>t_start)
```

//the list of used compartment in the module

```
  compartment Cytosol, Mitochondria;
```

//the list of used biochemical species in the module

```
  species ADP_c in Cytosol, ATP_c in Cytosol, CO2_c in Cytosol, CoA_c in Cytosol, FAC_c in Cytosol, H_c in Cytosol, NADH_c in Cytosol, NAD_c in Cytosol, O2_c in Cytosol, Pi_c in Cytosol, Pyr_c in Cytosol, ADP_m in Mitochondria, ATP_m in Mitochondria, CO2_m in Mitochondria, CoA_m in Mitochondria, FAC_m in Mitochondria, H_m in Mitochondria, NADH_m in Mitochondria, NAD_m in Mitochondria, O2_m in Mitochondria, Pi_m in Mitochondria, Pyr_m in Mitochondria;
```

//the list of used model parameters in the module

var ADP_tis, ADP_tis_R, ATP_tis, ATP_tis_R, A_CO2, A_C_m_FAC, A_H, A_O2, A_c_m_ATP, A_c_m_CoA, A_c_m_NAD, A_c_m_Pi, A_c_m_Pyr, A_leak, H_tis, J_CO2_R, J_H_R, J_O2_R, J_c_m_ADP_R, J_c_m_ATP_R, J_c_m_CoA_R, J_c_m_FAC_R, J_c_m_NAD_R, J_c_m_Pi_R, J_c_m_Pyr_R, J_leak_R, M_ADP_1, M_ADP_2, M_CoA, M_FAC, M_H, M_NADH_1, M_NADH_2, M_Pi, M_Pyr, O2_tis, O2_tis_R, PS_cyt, PS_mit, Pi_tis, Pi_tis_R, Pyr_tis, Pyr_tis_R, RS_cyt, RS_mit, T_ADP, T_CoA, T_FAC, T_H, T_NADH, T_Pi, T_Pyr, V, V_CO2_c, V_CO2_m, V_O2_c, V_O2_m, W, lambda_CO2, lambda_O2, lambda_leak, pH_R, t_start, unknown;

//the list of values of used model parameters in the module

```
//Initialization
Cytosol = 3.6;
Mitochondria = 0.4;
A_CO2 = 0.243650793650794;
A_C_m_FAC = 0.0448390383649842;
A_H = 0.0794936353702118;
A_O2 = 0.243650793650794;
A_c_m_ATP = 0.0968209338728255;
A_c_m_CoA = 0.0794936353702118;
A_c_m_NAD = 0.14013918012936;
A_c_m_Pi = 0.0968209338728255;
A_c_m_Pyr = 0.859222067987832;
M_ADP_1 = 666.3667042;
M_ADP_2 = 0.553571429;
M_CoA = 0.04;
M_FAC = 0.00175;
M_H = 2.51E-5;
M_NADH_1 = 6.17333E-4;
M_NADH_2 = 15.79365079;
M_Pi = 1.375;
M_Pyr = 0.0528;
T_ADP = 10.5028697345622;
T_CoA = 0.25696;
T_FAC = 0.159452857142857;
T_H = 5.28620939843403E-5;
T_NADH = 0.191870083148804;
T_Pi = 25.297305;
T_Pyr = 0.536887313463204;
V = 2.0;
lambda_CO2 = 7.72540983606558;
lambda_O2 = 179.148148148148;
lambda_leak = 1.70106821034608;
```

//the list of reactions in the module

```
//Reactions
R000095: ADP_m + ATP_c -> ADP_c + ATP_m; 0.0;
R000103: NADH_c + NAD_m -> NADH_m + NAD_c; 0.0;
R1: Pyr_c -> Pyr_m; 0.0;
R2: FAC_c -> FAC_m; 0.0;
R3: CoA_c -> CoA_m; 0.0;
R4: H_c -> H_m; 0.0;
R5: Pi_c -> Pi_m; 0.0;
R6: O2_c -> O2_m; 0.0;
R7: CO2_c -> CO2_m; 0.0;
```

//the list of used mathematical equations in the module

```
//Equations
ADP_c '= J_c_m_ATP_R/Cytosol;
ADP_m '= -J_c_m_ATP_R/Mitochondria;
ATP_c '= -J_c_m_ATP_R/Cytosol;
ATP_m '= J_c_m_ATP_R/Mitochondria;
CO2_c '= -J_CO2_R/V_CO2_c;
CO2_m '= J_CO2_R/V_CO2_m;
CoA_c '= -J_c_m_CoA_R/Cytosol;
CoA_m '= J_c_m_CoA_R/Mitochondria;
FAC_c '= -J_c_m_FAC_R/Cytosol;
FAC_m '= J_c_m_FAC_R/Mitochondria;
H_c '= -J_H_R/(Cytosol*1000.0);
H_m '= J_H_R/Mitochondria;
NADH_c '= -J_c_m_NAD_R/Cytosol;
NADH_m '= J_c_m_NAD_R/Mitochondria;
NAD_c '= J_c_m_NAD_R/Cytosol;
NAD_m '= -J_c_m_NAD_R/Mitochondria;
O2_c '= -J_O2_R/V_O2_c;
O2_m '= J_O2_R/V_O2_m;
Pi_c '= -J_c_m_Pi_R/Cytosol;
Pi_m '= J_c_m_Pi_R/Mitochondria;
Pyr_c '= -J_c_m_Pyr_R/Cytosol;
Pyr_m '= J_c_m_Pyr_R/Mitochondria;

ADP_tis_R := tissue_conc(ADP_c,ADP_m,Cytosol,Mitochondria);
ATP_tis_R := tissue_conc(ATP_c,ATP_m,Cytosol,Mitochondria);
Cytosol := 0.88*V;
H_tis := tissue_conc(H_c,H_m,Cytosol,Mitochondria);
J_CO2_R := lambda_CO2*(CO2_c-CO2_m)*stress(A_CO2,W,t_start,time);
J_H_R := Transport(T_H,M_H,H_c,H_m,A_H,W,t_start,time);
J_O2_R := lambda_O2*(O2_c-O2_m)*stress(A_O2,W,t_start,time);
```

```
  J_c_m_ATP_R := Exchange(T_ADP,M_ADP_1,M_ADP_2,ATP_c/ADP_c,ATP_m/ADP_m,A_c_m_ATP,W,t_start,time);
  J_c_m_CoA_R := Transport(T_CoA,M_CoA,CoA_c,CoA_m,A_c_m_CoA,W,t_start,time);
  J_c_m_FAC_R := Transport(T_FAC,M_FAC,FAC_c,FAC_m,A_C_m_FAC,W,t_start,time);
  J_c_m_NAD_R := Exchange(T_NADH,M_NADH_1,M_NADH_2,NADH_c/NAD_c,NADH_m/NAD_m,A_c_m_NAD,W,t_start,time);
  J_c_m_Pi_R := Transport(T_Pi,M_Pi,Pi_c,Pi_m,A_c_m_Pi,W,t_start,time);
  J_c_m_Pyr_R := Transport(T_Pyr,M_Pyr,Pyr_c,Pyr_m,A_c_m_Pyr,W,t_start,time);
  J_leak_R := lambda_leak*H_c/H_m*stress(A_leak,W,t_start,time);
  Mitochondria := 0.12*V;
  O2_tis_R := tissue_conc(O2_c,O2_m,Cytosol,Mitochondria);
  PS_cyt := ATP_c/ADP_c;
  PS_mit := ATP_m/ADP_m;
  Pi_tis_R := tissue_conc(Pi_c,Pi_m,Cytosol,Mitochondria);
  Pyr_tis_R := tissue_conc(Pyr_c,Pyr_m,Cytosol,Mitochondria);
  RS_cyt := NADH_c/NAD_c*5000.0;
  RS_mit := NADH_m/NAD_m*10.0;
  pH_R := 3.0-log(H_tis,10.0);
```

//the list of used model species and their alias

```
  //Titles
  ADP_c is "ADP";
  ATP_c is "ATP";
  CO2_c is "CO<sub>2";
  CoA_c is "CoA";
  FAC_c is "FAC";
  H_c is "H";
  NADH_c is "NADH";
  NAD_c is "NAD";
  O2_c is "O<sub>2";
  Pyr_c is "Pyr";
  ADP_m is "ADP";
  ATP_m is "ATP";
  CO2_m is "CO<sub>2";
  CoA_m is "CoA";
  FAC_m is "FAC";
  H_m is "H";
  NADH_m is "NADH";
  NAD_m is "NAD";
  O2_m is "O<sub>2";
  Pi_m is "Pi";
```

```
Pyr_m is "Pyr";

//SBGN Properties
@ADP_c.sbgnType = "simple chemical";
@ATP_c.sbgnType = "simple chemical";
@CO2_c.sbgnType = "simple chemical";
@CoA_c.sbgnType = "simple chemical";
@FAC_c.sbgnType = "simple chemical";
@H_c.sbgnType = "simple chemical";
@NADH_c.sbgnType = "simple chemical";
@NAD_c.sbgnType = "simple chemical";
@O2_c.sbgnType = "simple chemical";
@Pi_c.sbgnType = "simple chemical";
@Pyr_c.sbgnType = "simple chemical";
@ADP_m.sbgnType = "simple chemical";
@ATP_m.sbgnType = "simple chemical";
@CO2_m.sbgnType = "simple chemical";
@CoA_m.sbgnType = "simple chemical";
@FAC_m.sbgnType = "simple chemical";
@H_m.sbgnType = "simple chemical";
@NADH_m.sbgnType = "simple chemical";
@NAD_m.sbgnType = "simple chemical";
@O2_m.sbgnType = "simple chemical";
@Pi_m.sbgnType = "simple chemical";
@Pyr_m.sbgnType = "simple chemical";
end
```

c. Mathematical equations of the metabolic reactions in the Module "Transport: Cytosol-Mitochondria":

The inter-compartmental metabolites transport is described as passive or facilitated (carrier mediated) fluxes according to the original paper for this Module (Li et al., 2012). By analogy with metabolic rates all transport flux equations are multiplied by a linear function to consider exercise effect on transport processes: $Function(W) = 1 + \alpha_i * W * (1 - e^{\frac{t_{start}-t}{\tau_i}})$ (see above details for parameters meaning). The basic transport flux equation for passive (superscript p) diffusion of species i between cytosol and mitochondria is

$T^{p}_{cyt<->mit,type,i} = \lambda_{cyt<->mit,type,i} * \left(C_{cyt,type,i} - C_{mit,type,i}\right) * (1 + \alpha_i * W * (1 - e^{\frac{t_{start}-t}{\tau_i}}))$, where $\lambda_{cyt<->mit,type,i}$ is the permeability-surface area coefficient, $C_{cyt,type,i}$ and $C_{mit,type,i}$ are concentrations of the species i in cytosol and mitochondria, respectively; $i \in (CO_2, O_2)$ and $type \in (type\ I\ fiber, type\ II\ fiber)$, while for facilitated (superscript f) transport is

$$T^{f}_{cyt<->mit,type,i} = R_{\max_{transport}cyt<->mit,type,i} * \left(\frac{C_{cyt,type,i}}{K_{M_{cyt<->mit,i}} + C_{cyt,type,i}} - \frac{C_{mit,type,i}}{K_{M_{cyt<->mit,i}} + C_{mit,type,i}}\right) *$$

$* (1 + \alpha_i * W * (1 - e^{\frac{t_{start}-t}{\tau_i}}))$, where $R_{\max_{transport}cyt<->mit,type,i}$ is the maximal flux rate for facilitated transport, $C_{cyt,type,i}$ and $C_{mit,type,i}$ are concentrations of the species i in cytosol and mitochondria, respectively; $i \in (H^+, Pyr, FAC, CoA, P_i)$ and $type \in (type\ I\ fiber, type\ II\ fiber)$.

As mentioned above, to describe the transport of two coupled pairs ($RS = \frac{NADH}{NAD^+}$, $PS = \frac{ATP}{ADP}$) exchange transport equations have been used in the next view:

$$T^f_{cyt<\to mit,,typeNADH} = -T^f_{cyt<\to mit,type,NAD^+} = R_{\max_{transport}cyt<\to mit,type,\mathrm{RS}} * \left(\frac{RS_{cyt,type}}{K^{cyt}_{M_{cyt<\to mit,type,\mathrm{RS}}} + RS_{cyt,type}} - \frac{RS_{mit,type}}{K^{mit}_{M_{cyt<\to mit,type,\mathrm{RS}}} + RS_{mit,type}}\right) * (1 + \alpha_{i,type} * W * (1 - e^{\frac{t_{start}-t}{\tau_i}}))$$

$$T^f_{cyt<->mit,type,ATP} = -T^f_{cyt<->mit,type,ADP} = R_{\max_{transport}cyt<->mit,type,PS} * \left(\frac{PS_{cyt,type}}{K^{cyt}_{M_{cyt<->mit,type,PS}} + PS_{cyt,type}} - \frac{PS_{mit,type}}{K^{mit}_{M_{cyt<->mit,type,PS}} + PS_{mit,type}}\right) * (1 + \alpha_{i,type} * W * (1 - e^{\frac{t_{start}-t}{\tau_i}}))$$

Table S5. The model parameter values for metabolic reactions in the Module "Transport: Cytosol-Mitochondria" (type I fibers)

Species, i	$\lambda_{cyt<->mit,i}$, $l/min/kg$	$R_{max_{transport}cyt<\to mit,i}$, $mmol/min$	$K_{M_{cyt<\to mit,i}}$, $mmol/L$	α_i, W^{-1}	τ_i, min	Reference
CO_2	7.73	N/A	N/A	2.44E-01	4.0E-1	*Li et al., 2012*
O_2	179.15	N/A	N/A	2.44E-01	4.0E-1	*Li et al., 2012*
H^+	N/A	5.29E-05	2.51E-05	7.95E-02	4.0E-1	*Li et al., 2012*
Pyr	N/A	5.37E-01	5.28E-02	8.59E-01	4.0E-1	*Li et al., 2012*
FAC	N/A	1.59E-01	1.75E-03	4.48E-02	4.0E-1	*Li et al., 2012*
CoA	N/A	2.57E-01	4.00E-02	7.95E-02	4.0E-1	*Li et al., 2012*
P_i	N/A	2.53E+01	1.38E+00	9.68E-02	4.0E-1	*Li et al., 2012*
$NADH/NAD^+$	N/A	1.92E-01	6.17E-04 (*cyt*)<br>1.58E+01 (*mit*)	1.40E-01	4.0E-1	*Li et al., 2012*
ATP/ADP	N/A	1.05E+01	6.66E+02 (*cyt*)<br>5.54E-01 (*mit*)	9.68E-02	4.0E-1	*Li et al., 2012*

Table S6. The model parameter values for metabolic reactions in the Module "Transport: Cytosol-Mitochondria" (type II fibers)

Species, i	$\lambda_{cyt<->mit,i}$, $l/min/kg$	$R_{max_{transport}cyt<\to mit,i}$, $mmol/min$	$K_{M_{cyt<\to mit,i}}$, $mmol/L$	α_i, W^{-1}	τ_i, min	Reference
CO_2	7.73	N/A	N/A	2.44E-01*	4.0E-1	*Li et al., 2012*
O_2	179.15	N/A	N/A	2.44E-01*	4.0E-1	*Li et al., 2012*
H^+	N/A	5.29E-05	2.51E-05	7.95E-02	4.0E-1	*Li et al., 2012*
Pyr	N/A	9.75E-01	5.28E-02	8.59E-01*	4.0E-1	*Li et al., 2012*
FAC	N/A	3.09E-01	1.75E-03	4.48E-02	4.0E-1	*Li et al., 2012*
CoA	N/A	1.45E-01	4.00E-02	7.95E-02	4.0E-1	*Li et al., 2012*
P_i	N/A	4.90E+01	1.38E+00	9.68E-02*	4.0E-1	*Li et al., 2012*
$NADH/NAD^+$	N/A	1.92E-01	6.17E-04 (*cyt*)<br>1.58E+01 (*mit*)	1.08E+00*	4.0E-1	*Li et al., 2012*
ATP/ADP	N/A	9.66E+00	6.66E+02 (*cyt*)<br>5.54E-01 (*mit*)	9.68E-02*	4.0E-1	*Li et al., 2012*

** - the value was changed during the model fitting to experimental data;*

4. Rates of reactions and ODE system of the Module "Capillary Blood"

a. SBGN scheme of the module:

According to the original publication (Li et al., 2012) for the metabolic part of our integrated model, the capillary blood and tissue interstitial fluid domains are assumed to be in equilibrium with each other and the species concentrations in these two domains are considered as equal and the total effective blood volume (V_{bl}) comprises the total effective volume of capillary blood and interstitial fluid space. The "Capillary Blood" module (Figure S4) consists of the 9 reactions which represent the dynamic mass balance of chemical species in the blood domain including glucose, pyruvate, Lac, Ala, Glr, FFA, CO_2, O_2, and protons (H^+) and taking into account their concentration differences between arterial and capillary (equal to the venous) domains. The system of ordinary differential equations for the dynamic mass balance is presented below in the corresponding subsection.

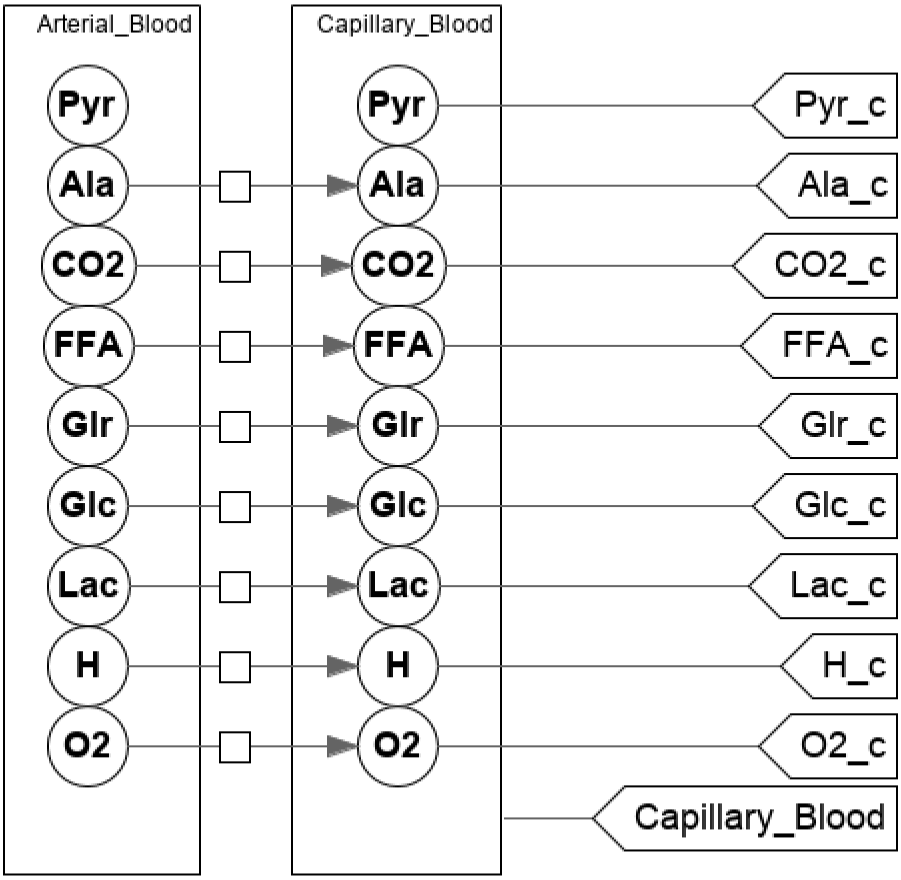

Figure S4. SBGN scheme of the reactions (vertex of the bipartite graph ⊣□⊢ corresponds to the reaction) taking place in the Module "Capillary Blood". All abbreviations and aliases of metabolites correspond to the above-mentioned descriptions. Specification for a certain metabolite to arterial and capillary blood domains' affiliation designated by "_a" and "_c", respectively. White arrows along the compartment border indicate contact ports for corresponding entities in the Module.

b. Antimony view which represents generated code according to the visual graph:

//name of the model and components

model
Capillary_Blood_Flow($Capillary_Blood.Ala_c,$Capillary_Blood.CO2_c,$Capillary_Blood,$Capillary_Blood.FFA_c,$Capillary_Blood.Glc_c,$Capillary_Blood.Glr_c,$Capillary_Blood.H_c,$Capillary_Blood.Lac_c,$Capillary_Blood.O2_c,$Capillary_Blood.Pyr_c,
>Q,<V_CO2_v,<V_O2_v,>V_mus)

//the list of used compartment in the module
compartment Arterial_Blood, Capillary_Blood;

//the list of used biochemical species in the module
species Ala_a in Arterial_Blood, CO2_a in Arterial_Blood, FFA_a in Arterial_Blood, Glc_a in Arterial_Blood, Glr_a in Arterial_Blood, H_a in Arterial_Blood, Lac_a in Arterial_Blood, O2_a in Arterial_Blood, Pyr_a in Arterial_Blood, Ala_c in Capillary_Blood, CO2_c in Capillary_Blood, FFA_c in Capillary_Blood, Glc_c in Capillary_Blood, Glr_c in Capillary_Blood, H_c in Capillary_Blood, Lac_c in Capillary_Blood, O2_c in Capillary_Blood, Pyr_c in Capillary_Blood;

//the list of used model parameters in the module
var C50HbCO_2, C50HbO_2, CHCO_3_a, CHCO_3_c, CHCO_3_pl_a, CHCO_3_pl_c, CHCO_3_rbc_a, CHCO_3_rbc_c, CH_isf, CH_pl, CH_rbc, CHbCO_2_Max, CHbCO_2_a, CHbCO_2_c, CHbO_2_Max, CHbO_2_a, CHbO_2_c, CHb_rbc, CO2_Tot_a, CO2_Tot_c, DCHCO_3_bl, DCHCO_3_isf, DCHCO_3_pl, DCHCO_3_rbc, DCHbCO_2_a, DCHbCO_2_c, DCHbO_2_a, DCHbO_2_c, DSHbCO_2_a, DSHbCO_2_c, DSHbO_2_a, DSHbO_2_c, Hct, KHbCO_2, KHbO_2, KeqCO_2_hyd, O2_Tot_a, O2_Tot_c, P50HbCO_2, P50HbO_2, Q, Q_Ala, Q_CO2, Q_FFA, Q_Glc, Q_Glr, Q_H, Q_Lac, Q_O2, Q_Pyr, R_cap, R_rbc, SHbCO_2_a, SHbCO_2_c, SHbO_2_a, SHbO_2_c, V_CO2_a, V_CO2_v, V_O2_a, V_O2_v, V_b, V_isf, V_mus, alphaCO_2, alphaO_2, nH, ph, ph_c;

//the list of values of used model parameters in the module
//Initialization
Arterial_Blood = 1.0;
Ala_a = 0.25;
CO2_a = 1.22;
FFA_a = 0.7;
Glc_a = 5.0;
Glr_a = 0.04;
H_a = 3.9810717E-5;
Lac_a = 0.5;
O2_a = 0.135;
Pyr_a = 0.08;
Capillary_Blood = 0.5;
Ala_c = 0.32222222;
CO2_c = 1.329987443;
FFA_c = 0.618888888;

```
   Glc_c = 4.78333333333;
   Glr_c = 0.048888889;
   H_c = 4.7863E-5;
   Lac_c = 0.6;
   O2_c = 0.04902533783;
   Pyr_c = 0.06666666666;
   C50HbO_2 = 26.8;
   CHb_rbc = 5.2;
   Hct = 0.45;
   KeqCO_2_hyd = 7.94E-4;
   P50HbCO_2 = 265.0;
   P50HbO_2 = 26.8;
   Q = 0.9;
   V_isf = 0.5;
   V_mus = 5.0;
   alphaCO_2 = 0.0305;
   alphaO_2 = 0.00135;
   nH = 2.7;

//the list of reactions in the module
   //Reactions
   R000026: Ala_a => Ala_c; 0.0;
   R000029: CO2_a => CO2_c; 0.0;
   R000032: FFA_a => FFA_c; 0.0;
   R000035: Glr_a => Glr_c; 0.0;
   R000038: Glc_a => Glc_c; 0.0;
   R000041: Lac_a => Lac_c; 0.0;
   R000044: H_a => H_c; 0.0;
   R000047: O2_a => O2_c; 0.0;

//the list of used mathematical equations in the module
   //Equations
   Ala_c '= Q_Ala/Capillary_Blood;
   CO2_c '= Q_CO2/V_CO2_v;
   FFA_c '= Q_FFA/Capillary_Blood;
   Glc_c '= Q_Glc/Capillary_Blood;
   Glr_c '= Q_Glr/Capillary_Blood;
   H_c '= Q_H/Capillary_Blood;
   Lac_c '= Q_Lac/Capillary_Blood;
   O2_c '= Q_O2/V_O2_v;
   Pyr_c '= Q_Pyr/Capillary_Blood;

   H_c = 10.0^(-4.32);

   C50HbCO_2 := alphaCO_2*P50HbCO_2;
```

```
C50HbO_2 := alphaO_2*P50HbO_2;
CHCO_3_a := (1.0-Hct)*CHCO_3_pl_a+Hct*CHCO_3_rbc_a;
CHCO_3_c := (1.0-Hct)*CHCO_3_pl_c+Hct*CHCO_3_rbc_c;
CHCO_3_pl_a := KeqCO_2_hyd*CO2_a/CH_pl;
CHCO_3_pl_c := KeqCO_2_hyd*CO2_c/CH_pl;
CHCO_3_rbc_a := R_rbc*CHCO_3_pl_a;
CHCO_3_rbc_c := R_rbc*CHCO_3_pl_c;
CH_isf := 10.0^(-7.2+3.0);
CH_pl := 10.0^(-7.4+3.0);
CH_rbc := 10.0^(-7.24+3.0);
CHbCO_2_Max := 4.0*Hct*CHb_rbc;
CHbCO_2_a := CHbCO_2_Max*SHbCO_2_a;
CHbCO_2_c := CHbCO_2_Max*SHbCO_2_c;
CHbO_2_Max := 4.0*Hct*CHb_rbc;
CHbO_2_a := CHbO_2_Max*SHbO_2_a;
CHbO_2_c := CHbO_2_Max*SHbO_2_c;
CO2_Tot_a := CO2_a+CHbCO_2_a+CHCO_3_a;
CO2_Tot_c := CO2_c+CHbCO_2_c+CHCO_3_c;
Capillary_Blood := V_b+V_isf;
DCHCO_3_bl := (1.0-Hct)*DCHCO_3_pl+Hct*DCHCO_3_rbc;
DCHCO_3_isf := R_cap*DCHCO_3_pl;
DCHCO_3_pl := KeqCO_2_hyd/CH_pl;
DCHCO_3_rbc := R_rbc*DCHCO_3_pl;
DCHbCO_2_a := CHbCO_2_Max*DSHbCO_2_a;
DCHbCO_2_c := CHbCO_2_Max*DSHbCO_2_c;
DCHbO_2_a := CHbO_2_Max*DSHbO_2_a;
DCHbO_2_c := CHbO_2_Max*DSHbO_2_c;
DSHbCO_2_a := KHbCO_2/(1.0+KHbCO_2*CO2_a)^2.0;
DSHbCO_2_c := KHbCO_2/(1.0+KHbCO_2*CO2_c)^2.0;
DSHbO_2_a := nH*KHbO_2*O2_a^(nH-1.0)/(1.0+KHbO_2*O2_a^nH)^2.0;
DSHbO_2_c := nH*KHbO_2*O2_c^(nH-1.0)/(1.0+KHbO_2*O2_c^nH)^2.0;
KHbCO_2 := 1.0/C50HbCO_2;
KHbO_2 := 1.0/C50HbO_2^nH;
O2_Tot_a := O2_a+CHbO_2_a;
O2_Tot_c := O2_c+CHbO_2_c;
Q_Ala := Q*(Ala_a-Ala_c);
Q_CO2 := Q*(CO2_Tot_a-CO2_Tot_c);
Q_FFA := Q*(FFA_a-FFA_c);
Q_Glc := Q*(Glc_a-Glc_c);
Q_Glr := Q*(Glr_a-Glr_c);
Q_H := Q*(H_a-H_c);
Q_Lac := Q*(Lac_a-Lac_c);
Q_O2 := Q*(O2_Tot_a-O2_Tot_c);
Q_Pyr := Q*(Pyr_a-Pyr_c);
R_cap := CH_pl/CH_isf;
```

```
R_rbc := CH_pl/CH_rbc;
SHbCO_2_a := KHbCO_2*CO2_a/(1.0+KHbCO_2*CO2_a);
SHbCO_2_c := KHbCO_2*CO2_c/(1.0+KHbCO_2*CO2_c);
SHbO_2_a := KHbO_2*O2_a^nH/(1.0+KHbO_2*O2_a^nH);
SHbO_2_c := KHbO_2*O2_c^nH/(1.0+KHbO_2*O2_c^nH);
V_CO2_a := V_b*(1.0+DCHbCO_2_a+DCHCO_3_bl)+V_isf*(1.0+DCHCO_3_isf);
V_CO2_v := V_b*(1.0+DCHbCO_2_c+DCHCO_3_bl)+V_isf*(1.0+DCHCO_3_isf);
V_O2_a := V_b*(1.0+DCHbO_2_a)+V_isf;
V_O2_v := V_b*(1.0+DCHbO_2_c)+V_isf;
V_b := 0.07*V_mus;
V_isf := 0.13*V_mus;
ph_c := 3.0-log(H_c,10.0);
```

//the list of used model species and their alias

```
//Titles
//Titles
Ala_a is "Ala";
CO2_a is "CO2";
FFA_a is "FFA";
Glc_a is "Glc";
Glr_a is "Glr";
H_a is "H";
Lac_a is "Lac";
O2_a is "O2";
Pyr_a is "Pyr";
Ala_c is "Ala";
CO2_c is "CO2";
FFA_c is "FFA";
Glc_c is "Glc";
Glr_c is "Glr";
H_c is "H";
Lac_c is "Lac";
O2_c is "O2";
Pyr_c is "Pyr";

//SBGN Properties
@Ala_a.sbgnType = "simple chemical";
@CO2_a.sbgnType = "simple chemical";
@FFA_a.sbgnType = "simple chemical";
@Glc_a.sbgnType = "simple chemical";
@Glr_a.sbgnType = "simple chemical";
@H_a.sbgnType = "simple chemical";
@Lac_a.sbgnType = "simple chemical";
@O2_a.sbgnType = "simple chemical";
@Pyr_a.sbgnType = "simple chemical";
```

```
        @Ala_c.sbgnType = "simple chemical";
        @CO2_c.sbgnType = "simple chemical";
        @FFA_c.sbgnType = "simple chemical";
        @Glc_c.sbgnType = "simple chemical";
        @Glr_c.sbgnType = "simple chemical";
        @H_c.sbgnType = "simple chemical";
        @Lac_c.sbgnType = "simple chemical";
        @O2_c.sbgnType = "simple chemical";
        @Pyr_c.sbgnType = "simple chemical";
end
```

c. Mathematical equations of the metabolic reactions in the Module "Capillary Blood":

The dynamic mass balance of a chemical species *i* in the Module "Capillary Blood" is originated from Li et al., 2012 and has the following general form:

$$V_{bl}\frac{dC_{bl,i}}{dt} = Q * \left(C_{art,i} - C_{bl,i}\right) - T^{f\ or\ p}_{bl<->cyt,R,i} * V_R - T^{f\ or\ p}_{bl<->cyt,W,i} * V_W$$

Where $T^{f\ or\ p}_{bl<->cyt,R,i}$ and $T^{f\ or\ p}_{bl<->cyt,W,i}$ (superscripts f and p denote facilitated and passive transports, respectively) are transport fluxes both between blood-cytosol of the type I and type II fibers, correspondingly; $C_{bl,i}$ and $C_{art,i}$ are the capillary blood species concentration and the arterial species concentration, respectively; V_{bl} is the total effective volume of blood and tissue interstitial fluid domains, $V_{bl} = 0.2 * V_{mus}, V_{tis} = 0.8 * V_{mus}$ where $V_{mus} = V_{tis} + V_b$ – the skeletal muscle volume (5 kg w.w.); Q is the blood flow ($Q_0 = 0.9$ L/min, muscle blood flow at rest for 2 legs). In response to an increase in the power, the muscle blood flow increases according to the generalized enhancement function: $Q = Q_0 * (1 + \alpha_i * W * (1 - e^{\frac{t_{start}-t}{\tau_i}}))$ (see above details for parameters meaning). Based on that the dynamic mass balance comprising all species in the Module are:

$$V_{bl}\frac{dC_{bl,Glc}}{dt} = Q * \left(C_{art,Glc} - C_{bl,Glc}\right) - T^{f}_{bl<->cyt,R,Glc} * V_R - T^{f}_{bl<->cyt,W,Glc} * V_W$$
$$V_{bl}\frac{dC_{bl,Pyr}}{dt} = Q * \left(C_{art,Pyr} - C_{bl,Pyr}\right) - T^{f}_{bl<->cyt,R,Pyr} * V_R - T^{f}_{bl<->cyt,W,Pyr} * V_W$$
$$V_{bl}\frac{dC_{bl,Lac}}{dt} = Q * \left(C_{art,Lac} - C_{bl,Lac}\right) - T^{f}_{bl<->cyt,R,Lac} * V_R - T^{f}_{bl<->cyt,W,Lac} * V_W$$
$$V_{bl}\frac{dC_{bl,Ala}}{dt} = Q * \left(C_{art,Ala} - C_{bl,Ala}\right) - T^{p}_{bl<->cyt,R,Ala} * V_R - T^{p}_{bl<->cyt,W,Ala} * V_W$$
$$V_{bl}\frac{dC_{bl,Glr}}{dt} = Q * \left(C_{art,Glr} - C_{bl,Glr}\right) - T^{p}_{bl<->cyt,R,Glr} * V_R - T^{p}_{bl<->cyt,W,Glr} * V_W$$
$$V_{bl}\frac{dC_{bl,FFA}}{dt} = Q * \left(C_{art,FFA} - C_{bl,FFA}\right) - T^{f}_{bl<->cyt,R,FFA} * V_R - T^{f}_{bl<->cyt,W,FFA} * V_W$$

$$V_{bl,CO_2}\frac{dC^F_{cyt,CO_2}}{dt} = Q * \left(C^T_{art,CO_2} - C^T_{bl,CO_2}\right) - T^p_{bl<->cyt,R,CO_2} * V_R - T^p_{bl<->cyt,W,CO_2} * V_W$$

$$V_{bl,O_2}\frac{dC^F_{cyt,O_2}}{dt} = Q * \left(C^T_{art,O_2} - C^T_{bl,O_2}\right) - T^p_{bl<->cyt,R,O_2} * V_R - T^p_{bl<->cyt,W,O_2} * V_W$$

$$V_{bl}\frac{dC_{bl,H^+}}{dt} = Q * \left(C_{art,H^+} - C_{bl,H^+}\right) - T^p_{bl<->cyt,R,H^+} * V_R - T^p_{bl<->cyt,W,H^+} * V_W$$

The rate equations for transport fluxes between blood and cytosol as well as kinetic parameter values (See Table S7 and Table S8) are presented below in the description of the Module "Transport: Blood-Cytosol". The derivations of kinetic laws for such variables as C^F_{cyt,CO_2}, C^F_{cyt,O_2} and C^T_{x,CO_2}, C^T_{x,O_2} ($x = art, bl$) corresponding to free (the superscript 'F') and total (the superscript 'T') concentrations of carbon dioxide and oxygen, respectively, as well as for the effective volumes or volumes of distributions of CO_2 and O_2 in compartments (capillary blood, cytosol and mitochondria): $V_{cyt,type,CO_2}, V_{mit,type,CO_2}, V_{bl,CO_2}$ and $V_{cyt,type,O_2}, V_{mit,type,O_2}, V_{bl,O_2}$, respectively are completely based on the mathematical formulas presented in the original publication for the metabolic module (Li et al., 2012). The parameter values used in these kinetic laws and initial values of the species concentrations in the arterial and capillary blood compartments precisely correspond to the original ones.

5. Rates of reactions and ODE system of the Module “Transport: Blood-Cytosol”

d. SBGN scheme of the module:

The “Transport: Blood-Cytosol” module (Figure S5) consists of the 9 transport reactions which represent facilitated transport of glucose, pyruvate, Lac, FFA and protons (H^+), passive transport reactions for CO_2, O_2, Ala and Glr. The list of reactions and math equations describing their rates are identical in type I and II fibers, while values of the kinetic parameters are different between two types of the fibers (See Table S7 and Table S8).

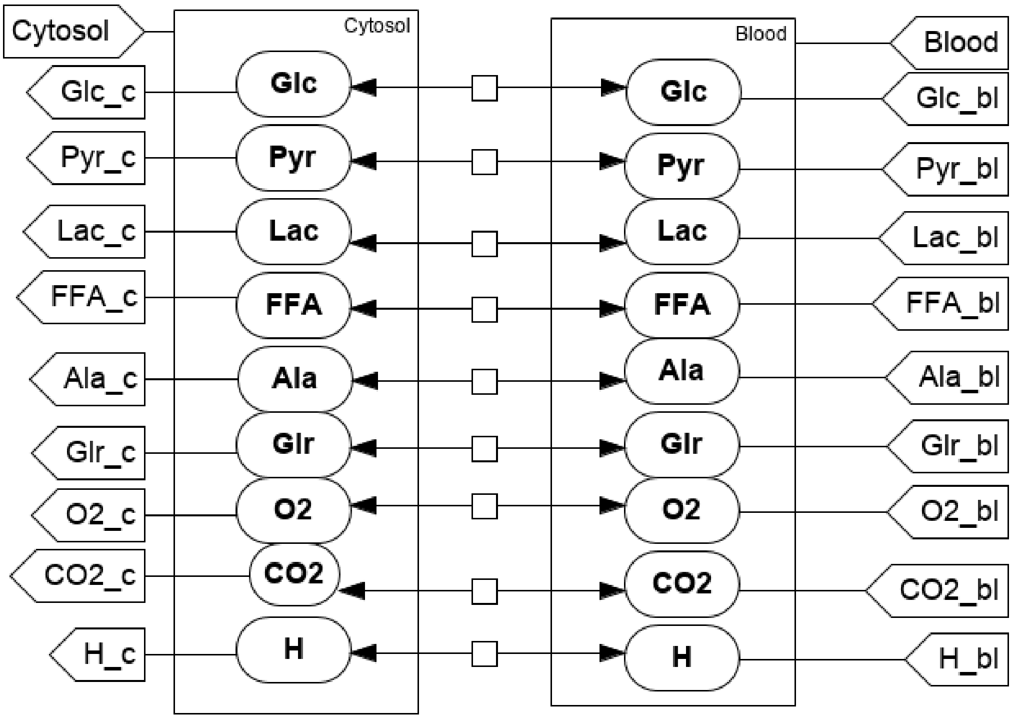

Figure S5. SBGN scheme of the transport reactions (vertex of the bipartite graph -□- corresponds to the reaction) taking place in the Module “Transport: Blood-Cytosol”. All abbreviations and aliases of metabolites correspond to the above-mentioned descriptions. Specification for a certain metabolite to cytosol and blood compartmental affiliation designated by “_c” and “_bl”, respectively. White arrows along the compartment border indicate contact ports for corresponding entities in the cytosol or in the blood.

e. Antimony view which represents generated code according to the visual graph:

//used mathematical functions in the module

```
function stress(a,W,t_start,t)
1.0+a*W*(1.0-exp((t_start-t)/0.4));
end

function Transport(T,M,C_1,C_2,a,W,t_start,t)
T*(C_1/(M+C_1)-C_2/(M+C_2))*stress(a,W,t_start,t);
end
```

//name of the model and components

//Function definitions
model
Cytosol_Capillary_Transport_R($Blood.Ala_bl,$Cytosol.Ala_c,$Blood,$Blood.CO2_bl,$Cytosol.CO2_c,$Cytosol,$Blood.FFA_bl,$Cytosol.FFA_c,$Blood.Glc_bl,$Cytosol.Glc_c,$Blood.Glr_bl,$Cytosol.Glr_c,$Blood.H_bl,$Cytosol.H_c,$Blood.Lac_bl,$Cytosol.Lac_c,$Blood.O2_bl,$Cytosol.O2_c,$Blood.Pyr_bl,$Cytosol.Pyr_c,>V_CO2_bl,>V_CO2_c,>V_O2_bl,>V_O2_c,>V_mus,>V_R,>W,>t_start)

//the list of used compartment in the module
compartment Blood, Cytosol;

//the list of used biochemical species in the module
species Ala_bl in Blood, CO2_bl in Blood, FFA_bl in Blood, Glc_bl in Blood, Glr_bl in Blood, H_bl in Blood, Lac_bl in Blood, O2_bl in Blood, Pyr_bl in Blood, Ala_c in Cytosol, CO2_c in Cytosol, FFA_c in Cytosol, Glc_c in Cytosol, Glr_c in Cytosol, H_c in Cytosol, Lac_c in Cytosol, O2_c in Cytosol, Pyr_c in Cytosol;

//the list of used model parameters in the module
var A_Ala, A_CO2, A_FFA, A_Glc, A_H, A_Lac, A_O2, A_Pyr, J_Ala, J_CO2, J_FFA, J_Glc, J_Glr, J_H, J_Lac, J_O2, J_Pyr, M_FFA, M_Glc, M_Lac, M_Pyr, M_bR_H, T_FFA, T_Glc, T_H, T_Lac, T_Pyr, V_CO2_bl, V_CO2_c, V_O2_bl, V_O2_c, V_R, V_mus, W, lambda_Ala, lambda_CO2, lambda_Glr, lambda_O2, t_start;

//the list of values of used model parameters in the module
//Initialization
Blood = 1.0;
Ala_bl = 0.322;
CO2_bl = 1.33;
FFA_bl = 0.619;
Glc_bl = 4.7833;
Glr_bl = 0.0489;
H_bl = 4.78E-5;
Lac_bl = 0.6;
O2_bl = 0.049;
Pyr_bl = 0.0617;
Cytosol = 3.6;
Ala_c = 1.4444;
CO2_c = 1.403;
FFA_c = 0.5;
Glc_c = 0.5556;
Glr_c = 0.0722;
H_c = 7.94E-5;
Lac_c = 0.8667;
O2_c = 0.0338;
Pyr_c = 0.0528;

```
A_Ala = 0.0555555555555;
A_CO2 = 0.243650793650794;
A_FFA = 0.0242063492063492;
A_Glc = 0.0304761904761905;
A_H = 0.02420634920635;
A_Lac = 0.180952380952381;
A_O2 = 0.243650793650794;
A_Pyr = 1.87380952380952;
M_FFA = 1.0;
M_Glc = 2.5;
M_Lac = 5.0;
M_Pyr = 1.0;
M_bR_H = 4.79E-5;
T_FFA = 0.522979717271052;
T_Glc = 0.206758177570094;
T_H = 2.92188186358879E-5;
T_Lac = 1.036;
T_Pyr = 0.531582089552239;
V_O2_c = 2.0;
V_R = 5.0;
V_mus = 5.0;
lambda_Ala = 0.0378084606345476;
lambda_CO2 = 12.9087384285338;
lambda_Glr = 0.160161779575329;
lambda_O2 = 79.1635519605644;
```

//the list of reactions in the module

```
//Reactions
R000004: Glc_c -> Glc_bl; 0.0;
R000007: Pyr_c -> Pyr_bl; 0.0;
R000010: Lac_c -> Lac_bl; 0.0;
R000013: FFA_c -> FFA_bl; 0.0;
R000016: Ala_c -> Ala_bl; 0.0;
R000019: Glr_c -> Glr_bl; 0.0;
R000022: O2_c -> O2_bl; 0.0;
R000025: CO2_c -> CO2_bl; 0.0;
R000028: H_c -> H_bl; 0.0;
```

//the list of used mathematical equations in the module

```
//Equations
Ala_bl '= J_Ala/Blood;
Ala_c '= -J_Ala/Cytosol;
CO2_bl '= J_CO2/V_CO2_bl;
CO2_c '= -J_CO2/V_CO2_c;
FFA_bl '= J_FFA/Blood;
```

```
FFA_c '= -J_FFA/Cytosol;
Glc_bl '= J_Glc/Blood;
Glc_c '= -J_Glc/Cytosol;
Glr_bl '= J_Glr/Blood;
Glr_c '= -J_Glr/Cytosol;
H_bl '= J_H/Blood;
H_c '= -J_H/(Cytosol*1000.0);
Lac_bl '= J_Lac/Blood;
Lac_c '= -J_Lac/Cytosol;
O2_bl '= J_O2/V_O2_bl;
O2_c '= -J_O2/V_O2_c;
Pyr_bl '= J_Pyr/Blood;
Pyr_c '= -J_Pyr/Cytosol;

Blood := V_mus*0.2;
Cytosol := 0.88*V_R;
J_Ala := lambda_Ala*(Ala_c-Ala_bl)*stress(A_Ala,W,t_start,time);
J_CO2 := lambda_CO2*(CO2_c-CO2_bl)*stress(A_CO2,W,t_start,time);
J_FFA := Transport(T_FFA,M_FFA,FFA_c,FFA_bl,A_FFA,W,t_start,time);
J_Glc := Transport(T_Glc,M_Glc,Glc_c,Glc_bl,A_Glc,W,t_start,time);
J_Glr := lambda_Glr*(Glr_c-Glr_bl);
J_H := Transport(T_H,M_bR_H,H_c,H_bl,A_H,W,t_start,time);
J_Lac := Transport(T_Lac,M_Lac,Lac_c,Lac_bl,A_Lac,W,t_start,time);
J_O2 := lambda_O2*(O2_c-O2_bl)*stress(A_O2,W,t_start,time);
J_Pyr := Transport(T_Pyr,M_Pyr,Pyr_c,Pyr_bl,A_Pyr,W,t_start,time);
```

//the list of used model species and their alias

```
 //Titles
Ala_bl is "Ala";
CO2_bl is "CO2";
FFA_bl is "FFA";
Glc_bl is "Glc";
Glr_bl is "Glr";
H_bl is "H";
Lac_bl is "Lac";
O2_bl is "O2";
Pyr_bl is "Pyr";
Ala_c is "Ala";
CO2_c is "CO2";
FFA_c is "FFA";
Glc_c is "Glc";
Glr_c is "Glr";
H_c is "H";
Lac_c is "Lac";
O2_c is "O2";
```

```
    Pyr_c is "Pyr";

    //SBGN Properties
    @Ala_bl.sbgnType = "simple chemical";
    @CO2_bl.sbgnType = "simple chemical";
    @FFA_bl.sbgnType = "simple chemical";
    @Glc_bl.sbgnType = "simple chemical";
    @Glr_bl.sbgnType = "simple chemical";
    @H_bl.sbgnType = "simple chemical";
    @Lac_bl.sbgnType = "simple chemical";
    @O2_bl.sbgnType = "simple chemical";
    @Pyr_bl.sbgnType = "simple chemical";
    @Ala_c.sbgnType = "simple chemical";
    @CO2_c.sbgnType = "simple chemical";
    @FFA_c.sbgnType = "simple chemical";
    @Glc_c.sbgnType = "simple chemical";
    @Glr_c.sbgnType = "simple chemical";
    @H_c.sbgnType = "simple chemical";
    @Lac_c.sbgnType = "simple chemical";
    @O2_c.sbgnType = "simple chemical";
    @Pyr_c.sbgnType = "simple chemical";
end
```

f. Mathematical equations of the metabolic reactions in the Module "Transport: Blood-Cytosol":

The inter-compartmental metabolites transport is described as passive or facilitated (carrier mediated) fluxes according to the original paper for this Module (Li et al., 2012). By analogy with metabolic rates all transport flux equations are multiplied by a linear function to consider exercise effect on transport processes: $Function(W) = 1 + \alpha_i * W * (1 - e^{\frac{t_{start} - t}{\tau_i}})$ (see above details for parameters meaning). The basic transport flux equation for passive (superscript p) diffusion of species i between the blood and cytosol is

$T^{p}_{bl<->cyt,type,i} = \lambda_{bl<->cyt,type,i} * \left(C_{bl,i} - C_{cyt,type,i}\right) * (1 + \alpha_i * W * (1 - e^{\frac{t_{start} - t}{\tau_i}}))$, where $\lambda_{bl<->cyt,type,i}$ is the permeability-surface area coefficient, $C_{bl,i}$ and $C_{cyt,type,i}$ are concentrations of the species i in the blood and cytosol, respectively; $i \in (CO_2, O_2, Ala, Glr)$ and $type \in (type\ I\ fiber, type\ II\ fiber)$, while for facilitated (superscript f) transport is

$$T^{f}_{bl<->cyt,type,i} = R_{\max_{transport} bl<->cyt,type,i} * \left(\frac{C_{bl,i}}{K_{M_{bl<->cyt,i}} + C_{bl,i}} - \frac{C_{cyt,type,i}}{K_{M_{bl<->cyt,i}} + C_{cyt,type,i}}\right) *$$

$* (1 + \alpha_i * W * (1 - e^{\frac{t_{start} - t}{\tau_i}}))$, where $R_{\max_{transport} cyt<->mit,type,i}$ is the maximal flux rate for facilitated transport, $C_{bl,i}$ and $C_{cyt,type,i}$ are concentrations of the species i in the blood and cytosol, respectively; $i \in (Glc, Pyr, Lac, FFA, H^+)$ and $type \in (type\ I\ fiber, type\ II\ fiber)$.

Table S7. The model parameter values for metabolic reactions in the Module "Transport: Blood-Cytosol" (type I fibers)

Species,i	$\lambda_{cyt<->mit,i}$, $l/min/kg$	$R_{max_{transport}cyt<\to mit,i}$, $mmol/min/kg$	$K_{M_{cyt<\to mit,i}}$, $mmol/kg$	α_i, W^{-1}	τ_i, min	Reference
CO_2	12.91	N/A	N/A	2.44E-01	4.0E-1	*Li et al., 2012*
O_2	79.16	N/A	N/A	2.44E-01	4.0E-1	*Li et al., 2012*
Ala	3.78E-02	N/A	N/A	5.56E-02	4.0E-1	*Li et al., 2012*
Glr	1.60E-01	N/A	N/A	0	4.0E-1	*Li et al., 2012*
Glc	N/A	2.07E-01	2.50E+00	3.05E-02	4.0E-1	*Li et al., 2012*
Pyr	N/A	5.32E-01	1.00E+00	1.87E+00	4.0E-1	*Li et al., 2012*
Lac	N/A	1.04E+00	5.00E+00	1.81E-01	4.0E-1	*Li et al., 2012*
FFA	N/A	5.23E-01	1.00E+00	2.42E-02	4.0E-1	*Li et al., 2012*
H^+	N/A	2.92E-05	4.79E-05	2.42E-02	4.0E-1	*Li et al., 2012*

Table S8. The model parameter values for metabolic reactions in the Module "Transport: Blood-Cytosol" (type II fibers)

Species,i	$\lambda_{cyt<->mit,i}$, $l/min/kg$	$R_{max_{transport}cyt<\to mit,i}$, $mmol/min/kg$	$K_{M_{cyt<\to mit,i}}$, $mmol/kg$	α_i, W^{-1}	τ_i, min	Reference
CO_2	12.91	N/A	N/A	2.44E-01	4.0E-1	*Li et al., 2012*
O_2	79.16	N/A	N/A	2.44E-01	4.0E-1	*Li et al., 2012*
Ala	2.37E-02	N/A	N/A	5.56E-02	4.0E-1	*Li et al., 2012*
Glr	1.84E-01	N/A	N/A	0	4.0E-1	*Li et al., 2012*
Glc	N/A	2.04E-01	2.50E+00	3.05E-02	4.0E-1	*Li et al., 2012*
Pyr	N/A	4.48E-01	1.00E+00	1.87E+00	4.0E-1	*Li et al., 2012*
Lac	N/A	1.19E+00	5.00E+00	1.81E-01	4.0E-1	*Li et al., 2012*
FFA	N/A	4.60E-01	1.00E+00	2.42E-02	4.0E-1	*Li et al., 2012*
H^+	N/A	2.92E-05	4.79E-05	2.42E-02	4.0E-1	*Li et al., 2012*

6. Rates of reactions in the Module "Ca-signaling pathway"

a. SBGN scheme of the module:

The "Ca-signaling pathway" module (Figure S6) consists of the cascade of 8 bimolecular reactions including Ca-Calmodulin complex formation, an activation of CAMKII (Ca^{2+}/calmodulin(CaM)-dependent protein kinase II) and CAMKKII kinases as well as Calcineurin (CaN), phosphorylation reactions for AMPK (AMP-activated protein Kinase), CREB (cAMP-Responsive Element-Binding protein) and PGC1α (Peroxisome proliferator-activated receptor Gamma Coactivator-1 alpha) factors and dephosphorylation of the CRTC (CREB-Regulated Transcription Coactivator). The list of reactions and math equations describing their rates are identical in type I and II fibers, while values of the kinetic parameters are different between two types of the fibers (See differences below).

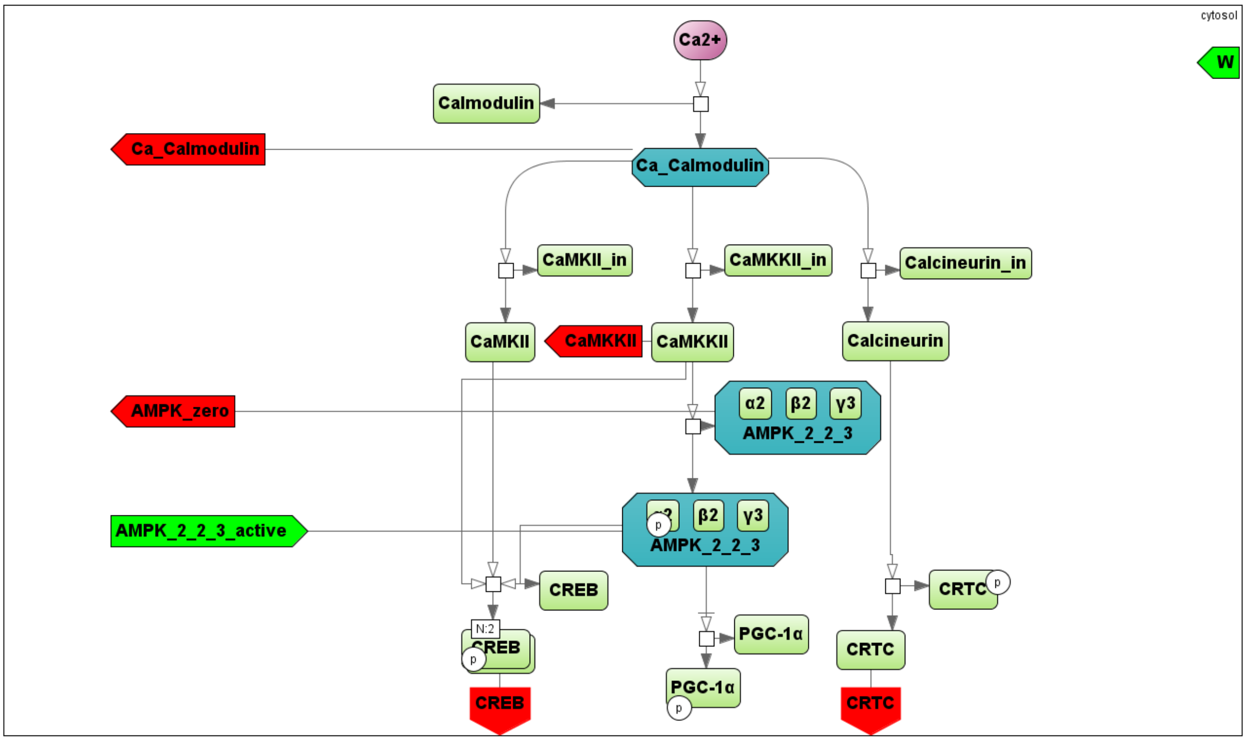

Figure S6. SBGN scheme of the signaling cascade reactions (vertex of the bipartite graph ─□─ corresponds to the reaction) taking place in the Module "Ca-signaling pathway". All abbreviations and aliases of proteins correspond to the above-mentioned descriptions. Red arrows (Ca_Calmodulin, CAMKKII, AMPK_zero, CREB and CRTC) correspond to output ports, while green arrow indicates input port (AMPK_2_2_3_active).

b. Antimony view which represents generated code according to the visual graph:

//name of the model and components

model Ca_signaling_pathway_R(>W,>$cytosol.AMPK_2_2_4,<$cytosol.AMPK_2_2_3,<$cytosol.CREB_1,<$cytosol.CRTC_1,<$cytosol.CaMKKII,<$cytosol.Ca_Calmodulin)

//the list of used compartment in the module

compartment cytosol;

//the list of used biochemical species in the module

species AMPK_2_2_3 in cytosol, AMPK_2_2_4 in cytosol, CREB in cytosol, CREB_1 in cytosol, CRTC in cytosol, CRTC_1 in cytosol, Ca2_ in cytosol, CaMKII in cytosol, CaMKII_in in cytosol, CaMKKII in cytosol, CaMKKII_in in cytosol, Ca_Calmodulin in cytosol, Calcineurin in cytosol, Calcineurin_in in cytosol, Calmodulin in cytosol, PGC_1alpha in cytosol, PGC_1alpha_1 in cytosol;

//the list of used model parameters in the module

var Ca_Calmodulin_R, Ca_Calmodulin_initial, FC_CAMK2_R, FC_CAMK2_R_1, FC_Ca_Calmodulin_R, K_AMPKin_toAMPKp, K_AMPKp_toAMPKin, K_CREB_toCREBp, K_CREBp_toCREB, K_CRTC_toCRTCp, K_CRTCp_toCRTC, K_CaCaM_assos, K_CaCaM_diss, K_CaMKII_toCaMKII_in, K_CaMKIIin_toCaMKII, K_CaMKKII_toCaMKKIIin, K_CaMKKIIin_toCaMKKII, K_CaN_toCaNin, K_CaNin_toCaN, K_PGC1alpha_toPGC1alphap, K_PGC1alphap_toPGC1alpha, K_X_protein_translation, K_basal_X, K_bind_PGC1a_func_prot, K_degr_PGC1a_prot, K_degr_mRNA_X_fact, K_transport_cyt_to_nucl, Perc_Ca_Calm_R, W, alpha_R, h_X, k_ex, unknown;

//the list of values of used model parameters in the module

//Initialization
cytosol = 1.0;
AMPK_2_2_3 = 0.36;
CREB = 0.23;
CRTC = 0.1;
Ca2_ = 0.1;
CaMKII_in = 1.0;
CaMKKII_in = 0.04;
Calcineurin_in = 0.003;
Calmodulin = 6.0;
PGC_1alpha = 0.1;
K_AMPKin_toAMPKp = 10.0;
K_AMPKp_toAMPKin = 0.01;
K_CREB_toCREBp = 0.1;
K_CREBp_toCREB = 0.1;
K_CRTC_toCRTCp = 0.15;
K_CRTCp_toCRTC = 0.15;
K_CaCaM_assos = 0.5;
K_CaCaM_diss = 100.0;
K_CaMKII_toCaMKII_in = 132.0;
K_CaMKIIin_toCaMKII = 3940.0;
K_CaMKKII_toCaMKKIIin = 132.0;
K_CaMKKIIin_toCaMKKII = 3940.0;
K_CaN_toCaNin = 0.072;

K_CaNin_toCaN = 2760.0;
K_PGC1alpha_toPGC1alphap = 0.1;
K_PGC1alphap_toPGC1alpha = 0.1;
K_X_protein_translation = 0.01;
K_basal_X = 0.24;
K_bind_PGC1a_func_prot = 0.0035;
K_degr_PGC1a_prot = 0.01;
K_degr_mRNA_X_fact = 0.05;
K_transport_cyt_to_nucl = 0.05;
alpha_R = 0.038;
h_X = 2.0;
k_ex = 1.0;

//the list of reactions in the module

//Reactions
R000008: Calmodulin -> Ca_Calmodulin;
K_CaCaM_assos*Calmodulin*k_ex^2.0*Ca2_^2.0-K_CaCaM_diss*Ca_Calmodulin;
R000018_mod: Ca2_-(R000008;
R000014: CaMKII_in -> CaMKII;
K_CaMKIIin_toCaMKII*CaMKII_in*Ca_Calmodulin-
K_CaMKII_toCaMKII_in*CaMKII;
R000114_mod: Ca_Calmodulin-(R000014;
R000020: CaMKKII_in -> CaMKKII;
K_CaMKKIIin_toCaMKKII*CaMKKII_in*Ca_Calmodulin-
K_CaMKKII_toCaMKKIIin*CaMKKII;
R000012_mod: Ca_Calmodulin-(R000020;
R000026: Calcineurin_in -> Calcineurin;
K_CaNin_toCaN*Calcineurin_in*Ca_Calmodulin-K_CaN_toCaNin*Calcineurin;
R000126_mod: Ca_Calmodulin-(R000026;
R000051: AMPK_2_2_3 -> AMPK_2_2_4;
K_AMPKin_toAMPKp*AMPK_2_2_3*CaMKKII-
K_AMPKp_toAMPKin*AMPK_2_2_4;
R000151_mod: CaMKKII-(R000051;
R000058: PGC_1alpha -> PGC_1alpha_1;
K_PGC1alpha_toPGC1alphap*PGC_1alpha*AMPK_2_2_4-
K_PGC1alphap_toPGC1alpha*PGC_1alpha_1;
R000158_mod: AMPK_2_2_4-(R000058;
R000065: CREB -> CREB_1;
K_CREB_toCREBp*CREB*CaMKII*CaMKKII*AMPK_2_2_4-
K_CREBp_toCREB*CREB_1;
R000165_mod: AMPK_2_2_4-(R000065;
R000165_mod: CaMKII-(R000065;
R000165_mod: CaMKKII-(R000065;
R000074: CRTC -> CRTC_1; K_CRTCp_toCRTC*CRTC*Calcineurin-
K_CRTC_toCRTCp*CRTC_1;

R000174_mod: Calcineurin-(R000074;

//Constants
const AMPK_2_2_3, CREB, CRTC, CaMKII_in, CaMKKII_in, Calcineurin_in, Calmodulin, PGC_1alpha;

//the list of used mathematical equations in the module
//Equations
Ca_Calmodulin_R := Ca_Calmodulin;
FC_CAMK2_R := CaMKII/CaMKII_in;
FC_CAMK2_R_1 := CaMKII/(CaMKII+CaMKII_in);
FC_Ca_Calmodulin_R := piecewise(1.0,time<120.0,Ca_Calmodulin/Ca_Calmodulin_initial,time>=120.0);
Perc_Ca_Calm_R := Ca_Calmodulin/(Ca_Calmodulin+Calmodulin);
k_ex := piecewise(1.0,W==0.0,1.0+alpha_R*W,W>0.0);

//the list of used model species and their alias
//Titles
AMPK_2_2_4 is "AMPK_2_2_3";
CREB_1 is "CREB";
CRTC_1 is "CRTC";
Ca2_ is "Ca2+";
PGC_1alpha is "PGC-1α";
PGC_1alpha_1 is "PGC-1α";

//SBGN Properties
@AMPK_2_2_3.sbgnType = "complex";
@AMPK_2_2_3.sbgnViewTitle = "alpha2:betta2:gamma3";
@AMPK_2_2_4.sbgnType = "complex";
@AMPK_2_2_4.sbgnViewTitle = "alpha2{p}:betta2:gamma3";
@CREB.sbgnType = "macromolecule";
@CREB_1.sbgnType = "macromolecule";
@CREB_1.sbgnViewTitle = "(CREB_1{p})2";
@CRTC.sbgnType = "macromolecule";
@CRTC.sbgnViewTitle = "CRTC{p}";
@CRTC_1.sbgnType = "macromolecule";
@Ca2_.sbgnType = "simple chemical";
@CaMKII.sbgnType = "macromolecule";
@CaMKII_in.sbgnType = "macromolecule";
@CaMKKII.sbgnType = "macromolecule";
@CaMKKII_in.sbgnType = "macromolecule";
@Ca_Calmodulin.sbgnType = "complex";
@Calcineurin.sbgnType = "macromolecule";
@Calcineurin_in.sbgnType = "macromolecule";
@Calmodulin.sbgnType = "macromolecule";

@PGC_1alpha.sbgnType = "macromolecule";
@PGC_1alpha_1.sbgnType = "macromolecule";
@PGC_1alpha_1.sbgnViewTitle = "PGC_1alpha_1{p}";
end

c. Mathematical equations of the metabolic reactions in the Module "Ca-signaling pathway":

The Ca^{2+}-dependent signaling pathway is described in the cytosol compartment using classic bimolecular kinetic laws for reactions rates. Thus, according to the theoretical basics, the rate of the reversible binding of Ca^{2+} with CaM protein (F_{CaCaM}) which is a trigger activating calcium signaling transduction pathway is represented by the next mathematical equation:

$$F_{CaCaM} = K_{CaCaM_{assoc}} * (K_{ex} * C_{Ca^{2+}})^2 * C_{CaM} - K_{CaCaM_{dis}} * C_{CaCaM}$$

where $K_{CaCaM_{assoc}} = 0.5\ \mu M^{-2} * min^{-1}$ and $K_{CaCaM_{dis}} = 100\ min^{-1}$ (Cui and Kaandorp, 2008) are the rate constants of association and dissociation of the complex, respectively; K_{ex} is the constant of sharp change of the calcium concentration due to the exercise. Initial values for $C_{Ca^{2+}}$, C_{CaM} are 0.1 μM and 6 μM, correspondingly (Saucerman and Bers, 2008; Eilers et al., 2014). Calmodulin can bind 4 calcium molecules, two to the carboxyl or C-terminal EF hand (forming Ca2CaM) and then two to the amino or N-terminal EF hand (forming Ca4CaM). There are several detailed kinetic models simulating different subnetworks of Ca-dependent signaling pathway in cardiac myocytes and soleus muscle (Cui and Kaandorp, 2008; Saucerman and Bers, 2008; Eilers et al., 2014). We extended the pathway network including AMPK, CREB and CRTC components, but assumed that minimal number of binding ions to activate the complex is two. In contrast to the metabolic rates and transport fluxes, the exercise impact on the signaling activation simulates as a linear dependence of the parameter K_{ex} on the work rate by means of piecewise function:

$K_{ex} = \begin{cases} 1.0 & if\ W = 0.0 \\ 1.0 + \alpha_{type} * W, & if\ W > 0.0 \end{cases}$, where α_{type} is the activation coefficient and $type \in (type\ I\ fiber, type\ II\ fiber)$: $\alpha_R = 0.038$ and $\alpha_W = 0.05$ (model fitting to experimental data on intensity-dependent increase in phosphorylation of CAMKII and AMPK α2 and γ3 isoforms. See Figure 8A-C and Figure 8F-H in the main text).

Rates of subsequent reactions of the signaling pathway are described by the next set of equations:

$$F_{CAMKII_{acivation}} = K_{CAMKII_{toCAMKII^*}} * C_{CAMKII} * C_{CaCaM} - K_{CAMKII^*_{toCAMKII}} * C_{CAMKII^*}$$

where $K_{CAMKII_{toCAMKII^*}} = 3940\ \mu M^{-1} * min^{-1}$ and $K_{CAMKII^*_{toCAMKII}} = 132\ min^{-1}$ (Saucerman and Bers, 2008) are forward and backward rate constants of the CAMKII activation ($F_{CAMKII_{acivation}}$), correspondingly, while C_{CAMKII} and C_{CAMKII^*} are concentrations of non-active and active forms of CAMKII, respectively. Initial value of the C_{CAMKII} equals to 1 μM (Saucerman and Bers, 2008; Eilers et al., 2014).

$$F_{CAMKKII_{acivation}} = K_{CAMKKII_{toCAMKKII^*}} * C_{CAMKKII} * C_{CaCaM} - K_{CAMKKII^*_{toCAMKKII}} * C_{CAMKKII^*}$$

where $K_{CAMKKII_{toCAMKKII^*}} = 3940\,\mu M^{-1} * min^{-1}$ and $K_{CAMKKII^*_{toCAMKKII}} = 132\,min^{-1}$ (as corresponding parameters for CAMKII) are forward and backward rate constants of the CAMKKII activation ($F_{CAMKKII_{acivation}}$), correspondingly, while $C_{CAMKKII}$ and $C_{CAMKKII^*}$ are concentrations of non-active and active forms of CAMKKII, respectively. Initial value of the $C_{CAMKKII}$ equals to 0.04 µM (Akberdin et al., 2020).

$$F_{CaN_{activation}} = K_{CaN_{CaN^*}} * C_{CaN} * C_{CaCaM} - K_{CaN^*_{toCaN}} * C_{CaN^*}$$

where $K_{CaN_{CaN^*}} = 2760\,\mu M^{-1} * min^{-1}$ and $K_{CaN^*_{toCaN}} = 0.072\,min^{-1}$ (Cui and Kaandorp, 2008) are forward and backward rate constants of the CaN activation ($F_{CaN_{activation}}$), correspondingly, while C_{CaN} and C_{CaN^*} are concentrations of non-active and active forms of CaN, respectively. Initial value of the C_{CaN} equals to 0.003 µM (Saucerman and Bers, 2008; Akberdin et al., 2020).

$$F_{AMPK_{phoshorylation}} = K_{AMPK_{AMPK^p}} * C_{AMPK} * C_{CaMKKII^*} - K_{AMPK^p_{AMPK}} * C_{AMPK^p}$$

where $K_{AMPK_{AMPK^p}} = 10\,\mu M^{-1} * min^{-1}$ and $K_{AMPK^p_{AMPK}} = 0.01\,min^{-1}$ (Sonntag et al., 2012) are forward and backward rate constants of the AMPK phosphorylation ($F_{AMPK_{phoshorylation}}$), correspondingly, while C_{AMPK} and C_{AMPK^p} are concentrations of non-phosphorylated and phosphorylated (active) forms of AMPK, respectively. Initial value of the C_{AMPK} is 0.36 µM (Akberdin et al., 2020), which is used in the Module "AMPK" to estimate initial concentrations of three AMPK isoforms via input-output ports or connections between submodules according to the meta-model structure in the BioUML platform (Kolpakov et al., 2019).

$$F_{CREB_{phoshorylation}} = K_{CREB_{CREB^p}} * C_{CREB} * C_{CAMKII^*} * C_{CaMKKII^*} * C_{AMPK^p} - K_{CREB^p_{CREB}} * C_{CREB^p}$$

where $K_{CREB_{CREB^p}} = 0.1\,\mu M^{-3} * min^{-1}$ and $K_{CREB^p_{CREB}} = 0.1\,min^{-1}$ (Akberdin et al., 2020) are forward and backward rate constants of the CREB phosphorylation ($F_{CREB_{phoshorylation}}$), correspondingly, while C_{CREB} and C_{CREB^p} are concentrations of non-phosphorylated and phosphorylated (active) forms of CREB, respectively. Initial value of the C_{CREB} is 0.23 µM (Akberdin et al., 2020).

$$F_{PGC1\alpha_{phoshorylation}} = K_{PGC1\alpha_{PGC1\alpha^p}} * C_{PGC1\alpha} * C_{AMPK^p} - K_{PGC1\alpha^p_{PGC1\alpha}} * C_{PGC1\alpha^p}$$

where $K_{PGC1\alpha_{PGC1\alpha^p}} = 0.1\,\mu M^{-1} * min^{-1}$ and $K_{PGC1\alpha^p_{PGC1\alpha}} = 0.1\,min^{-1}$ (Akberdin et al., 2020) are forward and backward rate constants of the PGC1α phosphorylation ($F_{PGC1\alpha_{phoshorylation}}$), correspondingly, while $C_{PGC1\alpha}$ and $C_{PGC1\alpha^p}$ are concentrations of non-phosphorylated and phosphorylated (active) forms of PGC1α, respectively. This reaction has been added to the model for further extensions and consideration some feedbacks between PGC1α, its transcription regulators and components on the metabolic level.

$$F_{CRTC_{dephoshorylation}} = K_{CRTC_{CRTC}^{p}} * C_{CRTC^p} * C_{CaN^*} - K_{CRTC_{CRTC^p}} * C_{CRTC}$$

where $K_{CRTC_{CRTC}^{p}} = 0.15\ \mu M^{-1} * min^{-1}$ and $K_{CRTC_{CRTC^p}} = 0.15\ min^{-1}$ (Akberdin et al., 2020) are forward and backward rate constants of the CRTC dephosphorylation ($F_{CRTC_{dephoshorylation}}$), correspondingly, while C_{CRTC} and C_{CRTC^p} are concentrations of non-phosphorylated and phosphorylated forms of CRTC, respectively. Initial value of the C_{CRTC^p} is 0.1 μM (Akberdin et al., 2020).

7. Rates of reactions in the Module “AMPK”

a. SBGN scheme of the module:

The AMP-activated protein kinase (AMPK) has an important role in the regulation of cellular energy homeostasis. The kinase is activated in response to an exercise due to a rise in the AMP:ATP ratio that occurs as a result of ATP concentration’s decrease. The AMPK kinase is a trimeric complex containing α/β/γ subunits. According to the experimental data (Birk and Wojtaszewski, 2006), only three AMPK α/β/γ-heterotrimers (α1/β2/γ1, α2/β2/γ1 and α2/β2/γ3) are present in human skeletal muscle, the approximate distribution of which can be estimated as ~15% α1/β2/γ1, ~65% α2/β2/γ1 and~20% α2/β2/γ3. These ratios were used to calculate initial concentration values of three isoforms in the Module based on the concentration of the overall AMPK (“AMPK_initial” in the Figure S7, see above). The protein content of different isoforms and their catalytic activities changes differently in response to exercise training. Moreover, activation of the kinase as well as of its downstream targets is fiber type-specific. To take into account all these facts and simulate different isoform-specific responses of the AMPK during an exercise and recovery, the “AMPK” module (Figure S7) has been integrated into the model. The diagram consists of the cascade of 3 biomolecular reactions for each isoform that includes 1) binding of AMP/ATP with AMPK γ subunit, which allosterically activates the complex; 2) subsequent phosphorylation of the activated complex by upstream LKB1 and CAMKKII kinases, 3) formation of the overall pool of activated and phosphorylated AMPK forms; and eventually, formation of the generalized pool of all AMPK isoforms. Last two reactions were included in the model for further analysis but did not fitted in the current version of the integrated model. The list of reactions and math equations describing their rates are identical in type I and II fibers, while values of the kinetic parameters are different between two types of the fibers (See differences below).

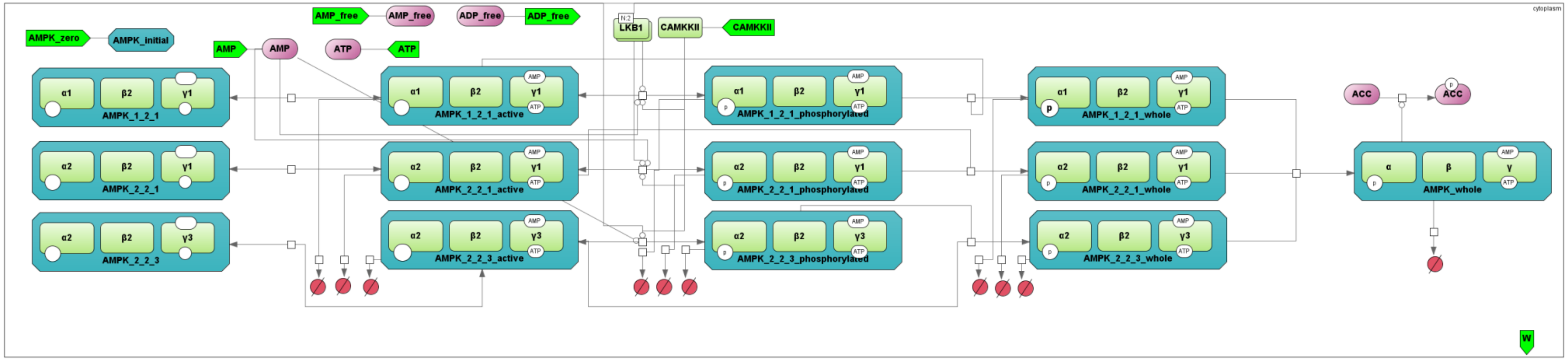

Figure S7. SBGN scheme of the cascade activation and phosphorylation reactions (vertex of the bipartite graph corresponds to the reaction) taking place in the Module "AMPK". All abbreviations and aliases of proteins correspond to the above-mentioned descriptions. Each subunit of the AMPK isoform is depicted on the diagram to specify the binding region of AMP/ATP and phosphorylation of the certain subunit by kinases. Products of some reactions or dead ends are results of the degradation processes. Green arrows (CAMKKII, AMPK_zero, AMP, ATP, AMP_free, ADP_free and W) correspond to input ports.

b. Antimony view which represents generated code according to the visual graph:

//name of the model and components

model
AMPK_module_R(>$cytoplasm.ADP_free_R,>$cytoplasm.AMPK_initial,>$cytoplasm.AMP_1,>$cytoplasm.AMP_free_R,>$cytoplasm.ATP_1,>$cytoplasm.CAMKKII,>W)

//the list of used compartment in the module

compartment cytoplasm;

//the list of used biochemical species in the module

species ACC in cytoplasm, ACC_1 in cytoplasm, ADP_free_R in cytoplasm, AMPK_1_2_1 in cytoplasm, AMPK_1_2_10 in cytoplasm, AMPK_1_2_11 in cytoplasm, AMPK_1_2_12 in cytoplasm, AMPK_1_2_13 in cytoplasm, AMPK_1_2_2 in cytoplasm, AMPK_1_2_3 in cytoplasm, AMPK_1_2_4 in cytoplasm, AMPK_1_2_5 in cytoplasm, AMPK_1_2_6 in cytoplasm, AMPK_1_2_7 in cytoplasm, AMPK_1_2_8 in cytoplasm, AMPK_1_2_9 in cytoplasm, AMPK_initial in cytoplasm, AMP_1 in cytoplasm, AMP_ATP in cytoplasm, AMP_free_R in cytoplasm, ATP_1 in cytoplasm, CAMKKII in cytoplasm, LKB1 in cytoplasm;

//the list of used model parameters in the module

var AMPK_a1_ratio, AMPK_a2_g1_ratio, AMPK_a2_g3_ratio, AMPK_alpha2_ratio_R, AMPK_g1_activity_R, AMPK_g2_activity_R, AMPK_g3_activity_R, AMPK_gamma3_ratio_R, AMPK_ratio, K_AMPK1act_AMPK1whole, K_AMPK1p_AMPK1whole, K_AMPK2act_AMPK2whole, K_AMPK2p_AMPK2whole, K_AMPK3act_AMPK3whole, K_AMPK3p_AMPK3whole, K_AMPK_act, K_act_1, K_act_1_back, K_act_2, K_act_2_back, K_act_3, K_act_3_back, K_activity_iso_a1, K_activity_iso_a2_g1, K_activity_iso_a2_g3, K_degr_AMPK1act, K_degr_AMPK1p, K_degr_AMPK1whole, K_degr_AMPK2act, K_degr_AMPK2p, K_degr_AMPK2whole, K_degr_AMPK3act, K_degr_AMPK3p, K_degr_AMPK3whole, K_degr_AMPKwhole, K_m_18, K_m_19, K_m_AMPK_act, V__forward_AMPK1act_AMPK1p, V__forward_AMPK1act_AMPK1p_CAMKKII, V__forward_AMPK2act_AMPK2p, V__forward_AMPK2act_AMPK2p_CAMKKII, V__forward_AMPK3act_AMPK3p, V__forward_AMPK3act_AMPK3p_CAMKKII, V__max_forward_AMPK1act_AMPK1p, V__max_forward_AMPK1act_AMPK1p_CAMKKII, V__max_forward_AMPK2act_AMPK2p, V__max_forward_AMPK2act_AMPK2p_CAMKKII, V__max_forward_AMPK3act_AMPK3p, V__max_forward_AMPK3act_AMPK3p_CAMKKII, V_back_AMPK1p_AMPK1act, V_back_AMPK2p_AMPK2act, V_back_AMPK3p_AMPK3act, V_back_iso1, V_back_iso2, V_back_iso3, V_forward_iso1, V_forward_iso2, V_forward_iso3, W, alpha_R, h_1, h_1_back, h_2, h_2_back, h_3, h_3_back, unknown;

//the list of values of used model parameters in the module

//Initialization
cytoplasm = 1.0;

```
ACC = 0.5;
AMPK_1_2_1 = 8.0E-4;
AMPK_1_2_2 = 0.0034;
AMPK_1_2_3 = 1.3E-4;
LKB1 = 2.0E-4;
K_AMPK_act = 0.1;
K_act_1 = 0.25;
K_act_1_back = 0.25;
K_act_2 = 0.54;
K_act_2_back = 0.54;
K_act_3 = 0.44;
K_act_3_back = 100.0;
K_activity_iso_a1 = 440.0;
K_activity_iso_a2_g1 = 240.0;
K_activity_iso_a2_g3 = 120.0;
K_m_18 = 1.4;
K_m_19 = 0.067;
K_m_AMPK_act = 0.5;
V__forward_AMPK1act_AMPK1p = 1.2;
V__forward_AMPK1act_AMPK1p_CAMKKII = 1.2;
V__forward_AMPK2act_AMPK2p = 2.0;
V__forward_AMPK2act_AMPK2p_CAMKKII = 2.0;
V__forward_AMPK3act_AMPK3p = 0.1;
V__forward_AMPK3act_AMPK3p_CAMKKII = 0.1;
V_back_AMPK1p_AMPK1act = 0.032;
V_back_AMPK2p_AMPK2act = 0.032;
V_back_AMPK3p_AMPK3act = 0.006;
V_back_iso1 = 0.425;
V_back_iso2 = 0.425;
V_back_iso3 = 0.225;
V_forward_iso1 = 90.0;
V_forward_iso2 = 90.0;
V_forward_iso3 = 90.0;
alpha_R = 0.05;
h_1 = 1.0;
h_1_back = 1.0;
h_2 = 1.0;
h_2_back = 1.0;
h_3 = 2.0;
h_3_back = 4.0;
```

//the list of reactions in the module

```
  //Reactions
R000056: AMPK_1_2_2 -> AMPK_1_2_5;
V_forward_iso2*AMPK_1_2_2*AMP_ATP-V_back_iso2*AMPK_1_2_5;
```

R000060: AMPK_1_2_3 -> AMPK_1_2_6;
V_forward_iso3*AMPK_1_2_3*AMP_ATP-V_back_iso3*AMPK_1_2_6;
R000086: AMPK_1_2_4 -> AMPK_1_2_7;
V__forward_AMPK1act_AMPK1p*LKB1*(AMPK_1_2_4/(K_m_18+AMPK_1_2_4))*(AMP_1^h_1/K_act_1^h_1/(1.0+AMP_1^h_1/K_act_1^h_1))+V__forward_AMPK1act_AMPK1p_CAMKKII*CAMKKII*(AMPK_1_2_4/(K_m_18+AMPK_1_2_4))-V_back_AMPK1p_AMPK1act*(AMPK_1_2_7/(K_m_19+AMPK_1_2_7))*(1.0/(1.0+AMP_ATP^h_1_back/K_act_1_back^h_1_back));
R000186_mod: LKB1-(R000086;
R000186_mod: AMP_1-(R000086;
R000186_mod: CAMKKII-(R000086;
R000090: AMPK_1_2_5 -> AMPK_1_2_8;
V__forward_AMPK2act_AMPK2p*LKB1*(AMPK_1_2_5/(K_m_18+AMPK_1_2_5))*(AMP_1^h_2/K_act_2^h_2/(1.0+AMP_1^h_2/K_act_2^h_2))+V__forward_AMPK2act_AMPK2p_CAMKKII*CAMKKII*(AMPK_1_2_5/(K_m_18+AMPK_1_2_5))-V_back_AMPK2p_AMPK2act*(AMPK_1_2_8/(K_m_19+AMPK_1_2_8))*(1.0/(1.0+AMP_ATP^h_2_back/K_act_2_back^h_2_back));
R000019_mod: LKB1-(R000090;
R000019_mod: AMP_1-(R000090;
R000019_mod: CAMKKII-(R000090;
R000094: AMPK_1_2_6 -> AMPK_1_2_9;
V__forward_AMPK3act_AMPK3p*LKB1*(AMPK_1_2_6/(K_m_18+AMPK_1_2_6))*(AMP_1^h_3/K_act_3^h_3/(1.0+AMP_1^h_3/K_act_3^h_3))+V__forward_AMPK3act_AMPK3p_CAMKKII*CAMKKII*(AMPK_1_2_6/(K_m_18+AMPK_1_2_6))-V_back_AMPK3p_AMPK3act*(AMPK_1_2_9/(K_m_19+AMPK_1_2_9))*(1.0/(1.0+AMP_ATP^h_3_back/K_act_3_back^h_3_back));
R000194_mod: LKB1-(R000094;
R000194_mod: AMP_1-(R000094;
R000194_mod: CAMKKII-(R000094;
R000119: AMPK_1_2_4 + AMPK_1_2_7 => AMPK_1_2_10;
K_AMPK1p_AMPK1whole*AMPK_1_2_7+K_AMPK1act_AMPK1whole*AMPK_1_2_4;
R000123: AMPK_1_2_5 + AMPK_1_2_8 => AMPK_1_2_11;
K_AMPK2p_AMPK2whole*AMPK_1_2_8+K_AMPK2act_AMPK2whole*AMPK_1_2_5;
R000127: AMPK_1_2_6 + AMPK_1_2_9 => AMPK_1_2_12;
K_AMPK3p_AMPK3whole*AMPK_1_2_9+K_AMPK3act_AMPK3whole*AMPK_1_2_6;
R000138: AMPK_1_2_10 + AMPK_1_2_11 + AMPK_1_2_12 => AMPK_1_2_13;
AMPK_1_2_10+AMPK_1_2_11+AMPK_1_2_12;
R000143: AMPK_1_2_4 =>; K_degr_AMPK1act*AMPK_1_2_4;
R000147: AMPK_1_2_5 =>; K_degr_AMPK2act*AMPK_1_2_5;
R000151: AMPK_1_2_6 =>; K_degr_AMPK3act*AMPK_1_2_6;
R000155: AMPK_1_2_7 =>; K_degr_AMPK1p*AMPK_1_2_7;
R000159: AMPK_1_2_8 =>; K_degr_AMPK2p*AMPK_1_2_8;

R000163: AMPK_1_2_9 =>; K_degr_AMPK3p*AMPK_1_2_9;
R000167: AMPK_1_2_10 =>; K_degr_AMPK1whole*AMPK_1_2_10;
R000171: AMPK_1_2_11 =>; K_degr_AMPK2whole*AMPK_1_2_11;
R000175: AMPK_1_2_12 =>; K_degr_AMPK3whole*AMPK_1_2_12;
R000186: AMPK_1_2_13 =>; K_degr_AMPKwhole*AMPK_1_2_13;
R000196: ACC => ACC_1;
K_AMPK_act*ACC*AMPK_1_2_13/(K_m_AMPK_act+ACC);
R001196_mod: AMPK_1_2_13-(R000196;
R000209: AMPK_1_2_1 -> AMPK_1_2_4;
V_forward_iso1*AMPK_1_2_1*AMP_ATP-V_back_iso1*AMPK_1_2_4;

//Constants
const ACC, LKB1;

//the list of used mathematical equations in the module

//Equations
AMPK_1_2_1 := 0.15*AMPK_initial;
AMPK_1_2_2 := 0.65*AMPK_initial;
AMPK_1_2_3 := 0.2*AMPK_initial;
AMPK_a1_ratio := AMPK_1_2_7/(AMPK_1_2_1+AMPK_1_2_4+AMPK_1_2_7);
AMPK_a2_g1_ratio := AMPK_1_2_8/(AMPK_1_2_2+AMPK_1_2_5+AMPK_1_2_8);
AMPK_a2_g3_ratio := AMPK_1_2_9/(AMPK_1_2_3+AMPK_1_2_6+AMPK_1_2_9);
AMPK_alpha2_ratio_R :=
(AMPK_1_2_8+AMPK_1_2_9)/(AMPK_1_2_2+AMPK_1_2_3+AMPK_1_2_5+AMPK_1_2_6+AMPK_1_2_8+AMPK_1_2_9)*100.0;
AMPK_g1_activity_R := K_activity_iso_a1*AMPK_1_2_7;
AMPK_g2_activity_R := K_activity_iso_a2_g1*AMPK_1_2_8;
AMPK_g3_activity_R := K_activity_iso_a2_g3*AMPK_1_2_9;
AMPK_gamma3_ratio_R :=
AMPK_1_2_9/(AMPK_1_2_3+AMPK_1_2_6+AMPK_1_2_9)*100.0;
AMPK_ratio :=
(AMPK_1_2_7+AMPK_1_2_8+AMPK_1_2_9)/(AMPK_1_2_1+AMPK_1_2_2+AMPK_1_2_3+AMPK_1_2_4+AMPK_1_2_5+AMPK_1_2_6+AMPK_1_2_7+AMPK_1_2_8+AMPK_1_2_9);
AMP_ATP := piecewise(2.0*(AMP_1/ATP_1),W==175.0,AMP_1/ATP_1);
K_act_3 := piecewise(0.44,W==0.0,0.44,W>0.0);
V__forward_AMPK3act_AMPK3p := piecewise(0.1,W==0.0,0.1+alpha_R*W,W>0.0);
V__forward_AMPK3act_AMPK3p_CAMKKII :=
piecewise(0.1,W==0.0,0.1+alpha_R*W,W>0.0);
V__max_forward_AMPK1act_AMPK1p := piecewise(0.1,W==0.0,2.0,W>0.0);
V__max_forward_AMPK1act_AMPK1p_CAMKKII :=
piecewise(0.1,W==0.0,2.0,W>0.0);
V__max_forward_AMPK2act_AMPK2p := piecewise(0.1,W==0.0,2.0,W>0.0);
V__max_forward_AMPK2act_AMPK2p_CAMKKII :=
piecewise(0.1,W==0.0,2.0,W>0.0);

//the list of used model species and their alias

```
  //Titles
ACC_1 is "ACC";
AMPK_1_2_10 is "AMPK_1_2_1_whole";
AMPK_1_2_11 is "AMPK_2_2_1_whole";
AMPK_1_2_12 is "AMPK_2_2_3_whole";
AMPK_1_2_13 is "AMPK_whole";
AMPK_1_2_2 is "AMPK_2_2_1";
AMPK_1_2_3 is "AMPK_2_2_3";
AMPK_1_2_4 is "AMPK_1_2_1_active";
AMPK_1_2_5 is "AMPK_2_2_1_active";
AMPK_1_2_6 is "AMPK_2_2_3_active";
AMPK_1_2_7 is "AMPK_1_2_1_phosphorylated";
AMPK_1_2_8 is "AMPK_2_2_1_phosphorylated";
AMPK_1_2_9 is "AMPK_2_2_3_phosphorylated";
AMP_1 is "AMP";
ATP_1 is "ATP";

//SBGN Properties
@ACC.sbgnType = "simple chemical";
@ACC_1.sbgnType = "simple chemical";
@ADP_free_R.sbgnType = "simple chemical";
@AMPK_1_2_1.sbgnType = "complex";
@AMPK_1_2_1.sbgnViewTitle = "alpha:betta:gamma{}{}";
@AMPK_1_2_10.sbgnType = "complex";
@AMPK_1_2_10.sbgnViewTitle = "alpha:betta:gamma{AMP}{ATP}";
@AMPK_1_2_11.sbgnType = "complex";
@AMPK_1_2_11.sbgnViewTitle = "alpha:betta:gamma{AMP}{ATP}";
@AMPK_1_2_12.sbgnType = "complex";
@AMPK_1_2_12.sbgnViewTitle = "alpha:betta:gamma{AMP}{ATP}";
@AMPK_1_2_13.sbgnType = "complex";
@AMPK_1_2_13.sbgnViewTitle = "alpha:betta:gamma{AMP}{ATP}";
@AMPK_1_2_2.sbgnType = "complex";
@AMPK_1_2_2.sbgnViewTitle = "alpha:betta:gamma{}{}";
@AMPK_1_2_3.sbgnType = "complex";
@AMPK_1_2_3.sbgnViewTitle = "alpha:betta:gamma{}{}";
@AMPK_1_2_4.sbgnType = "complex";
@AMPK_1_2_4.sbgnViewTitle = "alpha:betta:gamma{AMP}{ATP}";
@AMPK_1_2_5.sbgnType = "complex";
@AMPK_1_2_5.sbgnViewTitle = "alpha:betta:gamma{AMP}{ATP}";
@AMPK_1_2_6.sbgnType = "complex";
@AMPK_1_2_6.sbgnViewTitle = "alpha:betta:gamma{AMP}{ATP}";
@AMPK_1_2_7.sbgnType = "complex";
@AMPK_1_2_7.sbgnViewTitle = "alpha:betta:gamma{AMP}{ATP}";
```

```
  @AMPK_1_2_8.sbgnType = "complex";
  @AMPK_1_2_8.sbgnViewTitle = "alpha:betta:gamma{AMP}{ATP}";
  @AMPK_1_2_9.sbgnType = "complex";
  @AMPK_1_2_9.sbgnViewTitle = "alpha:betta:gamma{AMP}{ATP}";
  @AMPK_initial.sbgnType = "complex";
  @AMP_1.sbgnType = "simple chemical";
  @AMP_ATP.sbgnType = "macromolecule";
  @AMP_free_R.sbgnType = "simple chemical";
  @ATP_1.sbgnType = "simple chemical";
  @CAMKKII.sbgnType = "macromolecule";
  @LKB1.sbgnType = "macromolecule";
  @LKB1.sbgnViewTitle = "(LKB1)2";
end
```

c. Mathematical equations of the metabolic reactions in the Module "AMPK":

AMPK activation and phosphorylation processes are described in the cytosol compartment using classic bimolecular kinetic laws for reactions rates. Thus, according to the theoretical basics, the rate of the reversible binding of AMP/ATP ($F_{i_{\frac{AMP}{ATP}}}$) with a particular AMPK isoform which allosterically activates the kinase is represented by the next mathematical equation:

$$F_{i_{\frac{AMP}{ATP}}} = K_{forward_{i_{\frac{AMP}{ATP}}}} * C_i * C_{\frac{AMP}{ATP}} - K_{backward_{i_{\frac{AMP}{ATP}}}} * C_{i_{\frac{AMP}{ATP}}}$$

where $i \in (\alpha1\beta2\gamma1, \alpha2\beta2\gamma1, \alpha2\beta2\gamma3)$, $K_{forward_{i_{\frac{AMP}{ATP}}}} = 90\ \mu M^{-1} * min^{-1}$ and $K_{backward_{\alpha1\beta2\gamma1_{\frac{AMP}{ATP}}}} = K_{backward_{\alpha2\beta2\gamma1_{\frac{AMP}{ATP}}}} = 0.425\ min^{-1}$; $K_{backward_{\alpha2\beta2\gamma3_{\frac{AMP}{ATP}}}} = 0.225\ min^{-1}$(Coccimiglio and Clarke, 2020). These parameter values are identical in both muscle fiber types. C_i and $C_{i_{\frac{AMP}{ATP}}}$ correspond to concentrations of non-active and allosterically activated forms of the *i* AMPK isoform.

The subsequent reversible reaction of the activated isoform phosphorylation and dephosphorylation has more complex rate equation ($F_{i_{phoshorylation}}$) due to the alternative phosphorylations by two kinases and allosteric regulation of the phosphorylation/dephosphorylation processes:

$$F_{i_{phoshorylation}} = K_{phoshorylation_{i_{LKB1}}} * C_{LKB1} * \frac{C_{i_{\frac{AMP}{ATP}}}}{K_{M_{LKB1}} + C_{i_{\frac{AMP}{ATP}}}} * \frac{\left(\frac{C_{AMP}}{K_{act_i}}\right)^{h_i}}{1 + \left(\frac{C_{AMP}}{K_{act_i}}\right)^{h_i}}$$

$$+ K_{phoshorylation_{i_{CAMKKII}}} * C_{CAMKKII} * \frac{C_{i_{\frac{AMP}{ATP}}}}{K_{M_{CAMKKII}} + C_{i_{\frac{AMP}{ATP}}}}$$

$$- K_{dephoshorylation_i} * \frac{C_{i_p}}{K_{M_{dp}} + C_{i_p}} * \frac{1}{1 + \left(\frac{C_{\frac{AMP}{ATP}}}{K_{act_{i_{dp}}}}\right)^{h_{i_{dp}}}}$$

where $i \in (\alpha1\beta2\gamma1, \alpha2\beta2\gamma1, \alpha2\beta2\gamma3)$, C_{i_p} is the concentration of corresponding phosphorylated AMPK isoform; $K_{phoshorylation_{i_{LKB1}}} = K_{phoshorylation_{i_{CAMKKII}}} = 1.2, 2.0 \text{ and } 0.1\ \mu M^{-1} * min^{-1}$, while $K_{dephoshorylation_i} = 0.032, 0.032 \text{ and } 0.006\ \mu M * min^{-1}$for $\alpha1\beta2\gamma1, \alpha2\beta2\gamma1$ and $\alpha2\beta2\gamma3$, correspondingly (Coccimiglio and Clarke, 2020) and values of the rate constants for different isoforms do not depend on the fiber type. $K_{M_{LKB1}} = 1.4\ \mu M$ and $K_{M_{CAMKKII}} = 0.067\ \mu M$ (Coccimiglio and Clarke, 2020) designate Michaelis constants for phosphorylation reactions by LKB1 and CAMKKII, respectively, which are also fiber type independent, while $C_{LKB1} = 0.2\ \mu M$ (data analysis of Popov et al., 2019 see in SM of Akberdin et al., 2020; an assumption is the LKB1 concentration does not significantly change) and $C_{CAMKKII}$ indicate concentrations of these kinases. $K_{act_i} = 0.25, 0.54 \text{ and } 0.44\ \mu M$ and $K_{act_{i_{dp}}} = 0.25, 0.54 \text{ and } 100\ \mu M$ (Coccimiglio and Clarke, 2020; and model fitting to experimental data on intensity-dependent increase in phosphorylation of AMPK α2 and γ3 isoforms. See description in the main text) correspond to threshold parameters of the allosteric regulation of the phosphorylation/dephosphorylation for $\alpha1\beta2\gamma1, \alpha2\beta2\gamma1$ and $\alpha2\beta2\gamma3$, correspondingly and values of these parameters are identical in both fiber types. h_i and $h_{i_{dp}}$are Hill coefficients of corresponding allosteric regulations: $h_{\alpha1\beta2\gamma1} = h_{\alpha1\beta2\gamma1_{dp}} = 1$, $h_{\alpha2\beta2\gamma1} = h_{\alpha2\beta2\gamma1_{dp}} = 1$,and $h_{\alpha2\beta2\gamma3} = 2$; $h_{\alpha2\beta2\gamma3_{dp}} = 4$ in both fiber types (model fitting). According to the experimental data, activation of $\alpha2\beta2\gamma3$ isoform of the AMPK complex due to the exercise is a major contributor to the response on the level of AMPK downstream targets. To take it into account we assumed that forward rates of the isoform phosphorylation by LKB1 and CAMKKII kinases leading to the formation of the active AMPK form change during the exercise as a linear dependence of these rates on the work rate by means of the piecewise function:

$$K_{phoshorylation_{\alpha2\beta2\gamma3_{LKB1}}} = K_{phoshorylation_{\alpha2\beta2\gamma3_{CAMKKII}}} = \begin{cases} 0.1 & if\ W = 0.0 \\ 0.1 + \alpha_{type} * W, & if\ W > 0.0 \end{cases},$$

where α_{type} is the activation coefficient and $type \in (type\ I\ fiber, type\ II\ fiber)$: $\alpha_R = 0.05$ and $\alpha_W = 0.08$ (model fitting to experimental data on intensity-dependent increase in phosphorylation of CAMKII and AMPK α2 and γ3 isoforms. See Figure 8A-C and Figure 8F-H in the main text).

To estimate the percentage of all α2 phosphorylated proteins and of the phosphorylated γ3 heterotrimers in type I fibers and type II fibers we used the next mathematical equations:

$$\alpha 2_{ratio_{type}} = \frac{C_{\alpha 2\beta 2\gamma 1_p} + C_{\alpha 2\beta 2\gamma 3_p}}{C_{\alpha 2\beta 2\gamma 1} + C_{\alpha 2\beta 2\gamma 3} + C_{\alpha 2\beta 2\gamma 1_{\frac{AMP}{ATP}}} + C_{\alpha 2\beta 2\gamma 3_{\frac{AMP}{ATP}}} + C_{\alpha 2\beta 2\gamma 1_p} + C_{\alpha 2\beta 2\gamma 3_p}} * 100\%$$

$$\gamma 3_{ratio_{type}} = \frac{C_{\alpha 2\beta 2\gamma 3_p}}{C_{\alpha 2\beta 2\gamma 3} + C_{\alpha 2\beta 2\gamma 3_{\frac{AMP}{ATP}}} + C_{\alpha 2\beta 2\gamma 3_p}} * 100\%$$

8. Rates of reactions in the Module "Gene expression regulation"

a. SBGN scheme of the module:

The module consists of the several submodules each of which describe the expression regulation of a certain gene (*NR4A2*, *NR4A3*, intermediate regulatory *X* factor and *PPARGC1α*. The general overview of the model is presented in Figure S8A, while diagrams for each submodule are depicted in Figure S8B-E. Thus, concentrations of the activated CREB and CRTC are links connecting levels of the signaling pathways and gene expression regulation (Figure S8A). The diagram of the expression regulation of X factor also contains a cytoplasm compartment to take into account a time delay required for such processes as the protein maturation and transport processes between nucleus and cytoplasm. The list of reactions and math equations describing their rates are identical in type I and II fibers, while values of the kinetic parameters are different between two types of the fibers (See differences below).

A

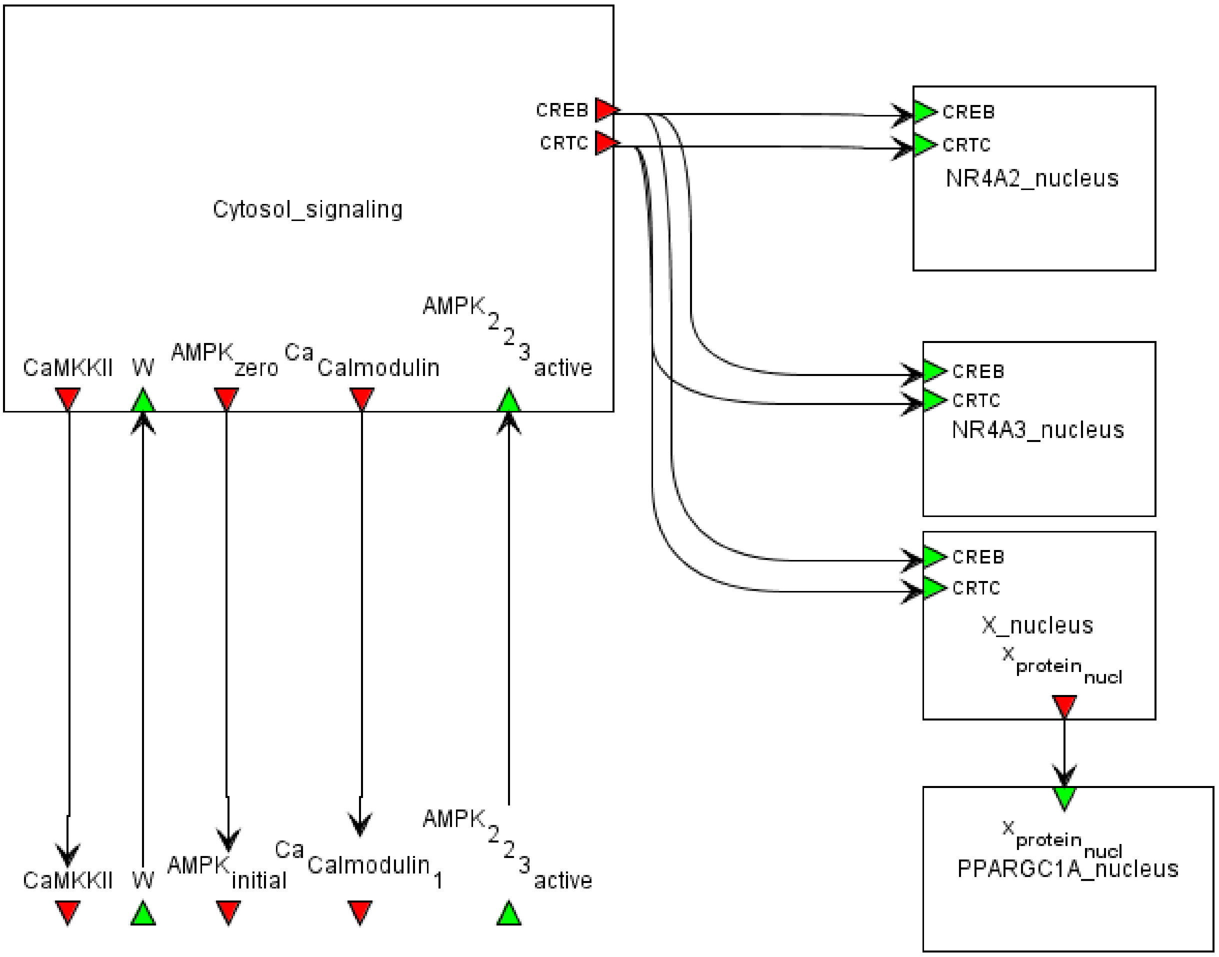

B

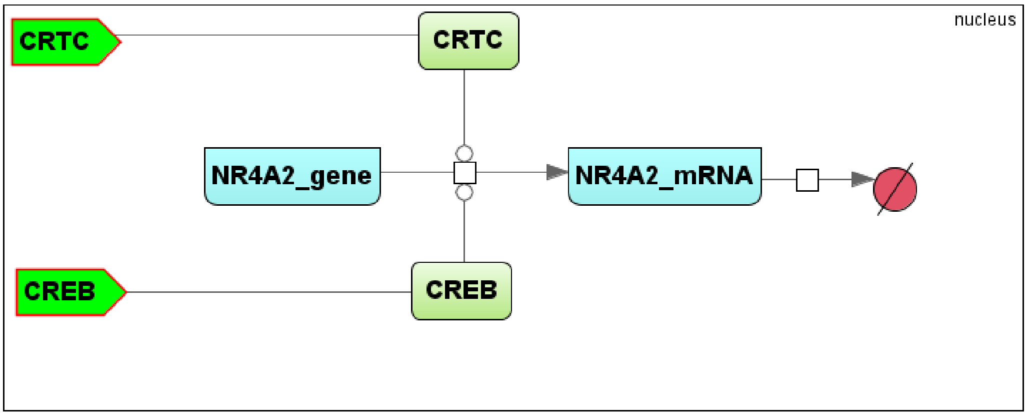

nucleus
CRTC
CRTC
NR4A2_gene
NR4A2_mRNA
CREB
CREB

C

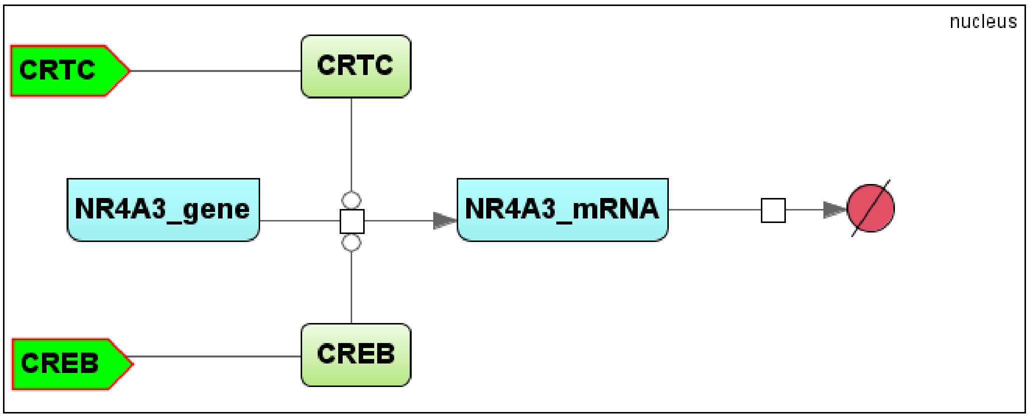

nucleus
CRTC
CRTC
NR4A3_gene
NR4A3_mRNA
CREB
CREB

D

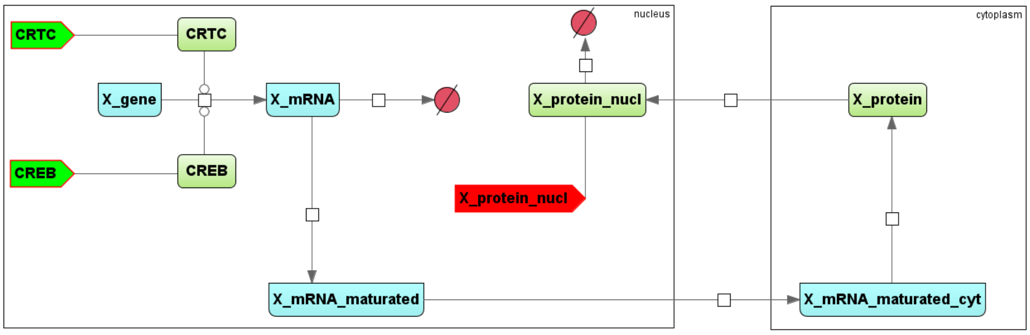

nucleus
cytoplasm
CRTC
CRTC
X_gene
X_mRNA
X_protein_nucl
X_protein
CREB
CREB
X_protein_nucl
X_mRNA_maturated
X_mRNA_maturated_cyt

E

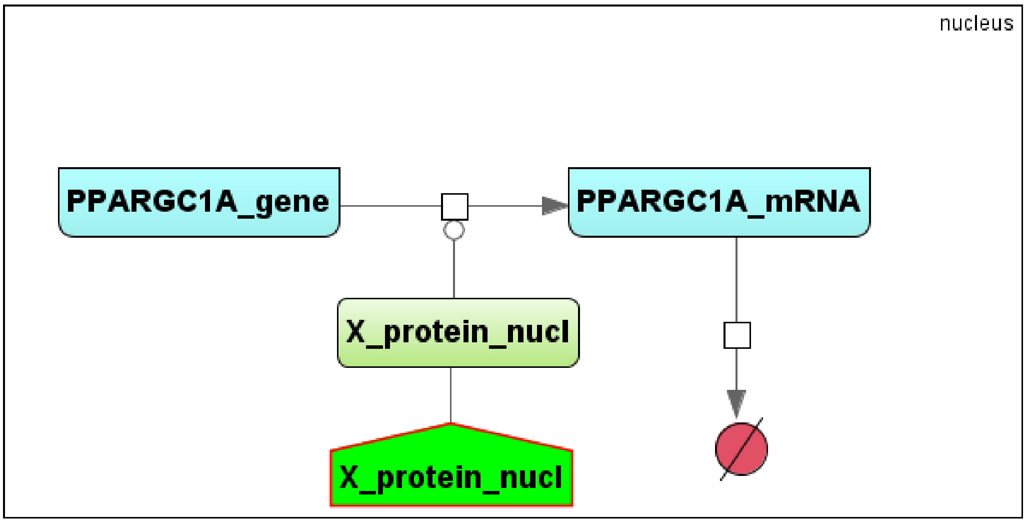

nucleus
PPARGC1A_gene
PPARGC1A_mRNA
X_protein_nucl
X_protein_nucl

Figure S8. SBGN scheme of reactions related to the gene expression regulation processes (vertex of the bipartite graph corresponds to the reaction) taking place in the Module "Gene expression regulation": A) general overview of the Module linking outcome of the signaling pathway (CREB and CRTC as TFs regulation expression of genes); B) a submodule of *NR4A2* expression regulation; C) a submodule of *NR4A3* expression regulation; D) a submodule of *X* factor expression regulation; E) a submodule of *PPARGC1α* expression regulation. The last submodule includes two alternative independent regulatory impacts by CREB and X factor to consider different hypothesis of the *PPARGC1α* expression regulation and fit the model simulations to the experimental data (Popov et al., 2019). All abbreviations and aliases of proteins correspond to the above-mentioned descriptions. Products of some reactions or dead ends are results of the degradation processes. Red arrows correspond to output ports from the compartment, while green arrows indicate input ports to the corresponding module.

b. Antimony view which represents generated code according to the visual graph:

for general module:

//name of the model and its submodules

```
model
Ca_signaling_R(>Ca_signaling_pathway_R.$cytosol.AMPK_2_2_4,<Ca_signaling_pathway_R.$cytosol.AMPK_2_2_3,<Ca_signaling_pathway_R.$cytosol.CaMKKII,<Ca_signaling_pathway_R.$cytosol.Ca_Calmodulin,>Ca_signaling_pathway_R.W)

  import "data/Collaboration (git)/Muscle metabolism/Models/Integrated Model 2021/Ca_signaling_pathway_R";
  Ca_signaling_pathway_R: Ca_signaling_pathway_R();

  import "data/Collaboration (git)/Muscle metabolism/Models/Integrated Model 2021/NR4A2_gene regulation_R";
  NR4A2: NR4A2_gene regulation_R();

  import "data/Collaboration (git)/Muscle metabolism/Models/Integrated Model 2021/NR4A3_gene regulation_R";
  NR4A3_gene regulation: NR4A3_gene regulation_R();

  import "data/Collaboration (git)/Muscle metabolism/Models/Integrated Model 2021/PPARGC1A_gene regulation_R";
  PPARGC1A_gene regulation: PPARGC1A_gene regulation_R();

  import "data/Collaboration (git)/Muscle metabolism/Models/Integrated Model 2021/X_gene regulation_R";
  X_gene regulation: X_gene regulation_R();


  //Connections
  NR4A2.CREB is Ca_signaling_pathway_R.CREB_1;
  NR4A2.CRTC is Ca_signaling_pathway_R.CRTC_1;
  NR4A3_gene regulation.CREB is Ca_signaling_pathway_R.CREB_1;
```

NR4A3_gene regulation.CRTC is Ca_signaling_pathway_R.CRTC_1;
X_gene regulation.CRTC is Ca_signaling_pathway_R.CRTC_1;
X_gene regulation.CREB is Ca_signaling_pathway_R.CREB_1;
PPARGC1A_gene regulation.X_protein_nucl is X_gene regulation.X_protein_nucl;
end

for *NR4A2* expression regulation:

//name of the model and components

model NR4A2_gene_regulation_R(>$nucleus.CREB,>$nucleus.CRTC)

//the list of used compartment in the module

compartment nucleus;

//the list of used biochemical species in the module

species CREB in nucleus, CRTC in nucleus;

//the list of used model parameters in the module

gene NR4A2_gene in nucleus, NR4A2_mRNA in nucleus;
var FC_NR4A2_R, K_basal_NR4A2, K_bind_1_CREB_NR4A2, K_bind_CREB, K_bind_CRTC, K_degr_mRNA_NR4A2, NR4A2_initial, W_bind_CREB_CRTC, h, h1;

//the list of values of used model parameters in the module

//Initialization
nucleus = 1.0;
NR4A2_gene = 1.0;
K_basal_NR4A2 = 0.5;
K_bind_1_CREB_NR4A2 = 0.001;
K_bind_CREB = 0.005;
K_bind_CRTC = 0.01;
K_degr_mRNA_NR4A2 = 0.01;
W_bind_CREB_CRTC = 0.1;
h = 2.0;
h1 = 2.0;

//the list of reactions in the module

R000005: NR4A2_mRNA =>; K_degr_mRNA_NR4A2*NR4A2_mRNA;
R000011: NR4A2_gene => NR4A2_mRNA; (K_basal_NR4A2+(CREB/K_bind_CREB)^h+W_bind_CREB_CRTC*(CREB/K_bind_CREB)^h*(CRTC/K_bind_CRTC)^h1)/(1.0+(CREB/K_bind_CREB)^h+W_bind_CREB_CRTC*(CREB/K_bind_CREB)^h*(CRTC/K_bind_CRTC)^h1)*((CREB/K_bind_1_CREB_NR4A2)^h/(1.0+(CREB/K_bind_1_CREB_NR4A2)^h));
R000111_mod: CREB-(R000011;
R000111_mod: CRTC-(R000011;

//Constants
const NR4A2_gene;

//the list of used model species and their alias
//SBGN Properties
@CREB.sbgnType = "macromolecule";
@CRTC.sbgnType = "macromolecule";
end

for *NR4A3* expression regulation:
//name of the model and components
model NR4A3_gene_regulation_R(>$nucleus.CREB,>$nucleus.CRTC)

//the list of used compartment in the module
compartment nucleus;

//the list of used biochemical species in the module
species CREB in nucleus, CRTC in nucleus;

//the list of used model parameters in the module
gene NR4A3_gene in nucleus, NR4A3_mRNA in nucleus;
var FC_NR4A3_R, K_basal_NR4A2, K_basal_NR4A3, K_bind_1_CREB_NR4A3_1, K_bind_CREB_NR4A3, K_bind_CRTC_NR4A3, K_degr_mRNA_NR4A3, NR4A3_initial, W_bind_CREB_CRTC, h, h1, h2;

//the list of values of used model parameters in the module
//Initialization
nucleus = 1.0;
NR4A3_gene = 1.0;
K_basal_NR4A2 = 0.5;
K_basal_NR4A3 = 0.25;
K_bind_1_CREB_NR4A3_1 = 1.0E-5;
K_bind_CREB_NR4A3 = 5.0E-5;
K_bind_CRTC_NR4A3 = 0.001;
K_degr_mRNA_NR4A3 = 0.07;
W_bind_CREB_CRTC = 0.1;
h = 2.0;
h1 = 2.0;
h2 = 2.0;

//the list of reactions in the module
//Reactions

NR4A3_regulation_expression: NR4A3_gene => NR4A3_mRNA; K_basal_NR4A3+(K_basal_NR4A2+(delay(CREB,0.5)/K_bind_CREB_NR4A3)^h+W_bind_CREB_CRTC*(delay(CREB,0.5)/K_bind_CREB_NR4A3)^h*(delay(CRTC,0.5)/K_bind_CRTC_NR4A3)^h1)/(1.0+(delay(CREB,0.5)/K_bind_CREB_NR4A3)^h+W_bind_CREB_CRTC*(delay(CREB,0.5)/K_bind_CREB_NR4A3)^h*(delay(CRTC,0.5)/K_bind_CRTC_NR4A3)^h1)*((delay(CREB,0.5)/K_bind_1_CREB_NR4A3_1)^h2/(1.0+(delay(CREB,0.5)/K_bind_1_CREB_NR4A3_1)^h2));
NR4A3_regulation_expression_mod1: CREB-(NR4A3_regulation_expression;
NR4A3_regulation_expression_mod1: CRTC-(NR4A3_regulation_expression;
R000004: NR4A3_mRNA =>; K_degr_mRNA_NR4A3*NR4A3_mRNA;

//Constants
const NR4A3_gene;

//the list of used model species and their alias
//SBGN Properties
@CREB.sbgnType = "macromolecule";
@CRTC.sbgnType = "macromolecule";
end

for *X* factor expression regulation:
//name of the model and components
model
X_gene_regulation_R(>$nucleus.CREB,>$nucleus.CRTC,<$nucleus.X_protein_nucl)

//the list of used compartment in the module
compartment cytoplasm, nucleus;

//the list of used biochemical species in the module
species X_protein in cytoplasm, CREB in nucleus, CRTC in nucleus, X_protein_nucl in nucleus;

//the list of used model parameters in the module
gene X_mRNA_maturated_cyt in cytoplasm, X_gene in nucleus, X_mRNA in nucleus, X_mRNA_maturated in nucleus;
var FC_X_R, K_X_mRNA_matur, K_X_protein_translation, K_basal_X_mRNA, K_bind_1_CREB, K_bind_CREB, K_bind_CRTC, K_degr_mRNA_X, K_degr_prot_nucl_X, K_transport_cyt_nucl, K_transport_nucl_cyt, W_bind_CREB_CRTC, X_factor_initial, h, h1;

//the list of values of used model parameters in the module
//Initialization

```
cytoplasm = 1.0;
nucleus = 1.0;
X_gene = 1.0;
K_X_mRNA_matur = 0.5;
K_X_protein_translation = 0.01;
K_basal_X_mRNA = 0.1;
K_bind_1_CREB = 0.01;
K_bind_CREB = 0.005;
K_bind_CRTC = 0.01;
K_degr_mRNA_X = 0.01;
K_degr_prot_nucl_X = 0.01;
K_transport_cyt_nucl = 0.05;
K_transport_nucl_cyt = 0.05;
W_bind_CREB_CRTC = 0.1;
h = 2.0;
h1 = 2.0;
```

//the list of reactions in the module

```
  //Reactions
R000026: X_mRNA_maturated => X_mRNA_maturated_cyt;
K_transport_nucl_cyt*X_mRNA_maturated;
R000034: X_protein => X_protein_nucl; K_transport_cyt_nucl*X_protein;
R000030: X_mRNA_maturated_cyt => X_protein;
K_X_protein_translation*X_mRNA_maturated_cyt;
R000005: X_mRNA =>; K_degr_mRNA_X*X_mRNA;
R000011: X_gene => X_mRNA;
(K_basal_X_mRNA+(CREB/K_bind_CREB)^h+W_bind_CREB_CRTC*(CREB/K_bind
_CREB)^h*(CRTC/K_bind_CRTC)^h1)/(1.0+(CREB/K_bind_CREB)^h+W_bind_CREB
_CRTC*(CREB/K_bind_CREB)^h*(CRTC/K_bind_CRTC)^h1)*((CREB/K_bind_1_CR
EB)^h/(1.0+(CREB/K_bind_1_CREB)^h));
R000111_mod: CREB-( R000011;
R000111_mod: CRTC-( R000011;
R000021: X_mRNA => X_mRNA_maturated; K_X_mRNA_matur*X_mRNA;
R000039: X_protein_nucl =>; K_degr_prot_nucl_X*X_protein_nucl;

//Constants
const X_gene;
```

//the list of used model species and their alias

```
 //SBGN Properties
@X_protein.sbgnType = "macromolecule";
@CREB.sbgnType = "macromolecule";
@CRTC.sbgnType = "macromolecule";
@X_protein_nucl.sbgnType = "macromolecule";
```

end

for *PPARGC1α* expression regulation:
//name of the model and components
model PPARGC1A_gene_regulation_R(>$nucleus.CREB,>$nucleus.X_protein_nucl)

//the list of used compartment in the module
compartment nucleus;

//the list of used biochemical species in the module
species CREB in nucleus, X_protein_nucl in nucleus;

//the list of used model parameters in the module
gene PPARGC1A_gene in nucleus, PPARGC1A_mRNA in nucleus;
var FC_PGC1A_R, K_basal_PPARGC1A, K_bind_CREB, K_bind_X, K_degr_mRNA_PPARGC1A, PGC1A_initial, h_X;

//the list of values of used model parameters in the module
//Initialization
nucleus = 1.0;
PPARGC1A_gene = 1.0;
K_basal_PPARGC1A = 0.24;
K_bind_CREB = 7.0E-5;
K_bind_X = 3.5E-4;
K_degr_mRNA_PPARGC1A = 0.05;
h_X = 2.0;

//the list of reactions in the module
//Reactions
R000008: PPARGC1A_gene => PPARGC1A_mRNA; (K_basal_PPARGC1A+(X_protein_nucl/K_bind_X)^h_X+(CREB/K_bind_CREB)^h_X)/(1.0+(X_protein_nucl/K_bind_X)^h_X+(CREB/K_bind_CREB)^h_X);
R000018_mod: X_protein_nucl-(R000008;
R000018_mod: CREB-(R000008;
R000012: PPARGC1A_mRNA =>; K_degr_mRNA_PPARGC1A*PPARGC1A_mRNA;

//Constants
const PPARGC1A_gene;

//the list of used model species and their alias
//SBGN Properties
@CREB.sbgnType = "macromolecule";
@X_protein_nucl.sbgnType = "macromolecule";
end

c. Mathematical equations of the metabolic reactions in the Module "Gene expression regulation":

As mentioned above, each submodule of the "Gene expression regulation" module represents a transcription regulation of the corresponding gene taking into account mRNA transcription and degradation processes. Thus, the transcription rate of the *NR4A2* gene ($F_{NR4A2_{transcription}}$) comprises 1) basal transcription proportionally of the constant, $K_{NR4A2_{basal}} = 0.5\,\mu M * min^{-1}$ (the same for both fiber types) and 2) non-competitive binding of the active CREB forms in two different sites taking into account cooperative effect of the CREB-CRTC bindings in one of the sites assuming analogous regulatory mechanism as for *PPARGC1α* transcription (Popov, 2018; Berdeaux and Hutchins, 2019). The rate is described by the next equation:

$$F_{NR4A2_{transcription}} = \frac{K_{NR4A2_{basal}} + \left(\frac{C_{CREB^p}}{K_{NR4A2_{CREB^p}}}\right)^h + w_{NR4A2_{CREB^p+CRTC}} * \left(\frac{C_{CREB^p}}{K_{NR4A2_{CREB^p}}}\right)^h * \left(\frac{C_{CRTC}}{K_{NR4A2_{CRTC}}}\right)^{h_1}}{1 + \left(\frac{C_{CREB^p}}{K_{NR4A2_{CREB^p}}}\right)^h + w_{NR4A2_{CREB^p+CRTC}} * \left(\frac{C_{CREB^p}}{K_{NR4A2_{CREB^p}}}\right)^h * \left(\frac{C_{CRTC}}{K_{NR4A2_{CRTC}}}\right)^{h_1}} * \frac{\left(\frac{C_{CREB^p}}{K_{NR4A2_{CREB^p}{}_1}}\right)^h}{1 + \left(\frac{C_{CREB^p}}{K_{NR4A2_{CREB^p}{}_1}}\right)^h}$$

where $K_{NR4A2_{CREB^p}} = 0.005\,\mu M$ and $K_{NR4A2_{CRTC}} = 0.01\,\mu M$ designate effective dissociation constants between the active CREB and CRTC forms and their binding sites of the *NR4A2* gene, respectively, while $K_{NR4A2_{CREB^p}{}_1} = 0.001\,\mu M$ is the effective dissociation constant between the active CREB form and its alternative binding site of the *NR4A2* gene; $h = h_1 = 2$ correspond to Hill coefficients for *NR4A2* transcription regulation by the active forms of CREB and CRTC factors, respectively; $w_{NR4A2_{CREB^p+CRTC}} = 0.1$ is a constant of the cooperative effect upon an joint impact of CREB and CRTC factors on *NR4A2* transcription. Values of all parameters are identical in both fiber types and based on the model fitting to the experimentally measured transcription dynamics of these genes as a response to the exercise (Popov et al., 2019). The rate of NR4A2 mRNA degradation is described as:

$$F_{NR4A2_{degradation}} = K_{NR4A2_{degradation}} * C_{NR4A2_{mRNA}}$$

where $K_{NR4A2_{degradation}} = 0.01\,min^{-1}$ is the degradation constant the value of which is identical in both fiber types.

The transcription dynamics of the *NR4A3* gene ($F_{NR4A3_{transcription}}$) demonstrates some time delay compared to the *NR4A3* gene transcription (Popov et al., 2019), while transcription factors regulating its transcription are also CREB and CRTC proteins. To consider the fact we assumed the *NR4A3* transcription rate is based on the same kinetic law but with some time delay ($\tau = 0.5\ min$) of the CREB and CRTC regulations. The rate is described by the next equation:

$$F_{NR4A3_{transcription}} = K_{NR4A3_{basal}} + \frac{K_{NR4A2_{basal}} + \left(\frac{C_{CREB^p}(t-\tau)}{K_{NR4A3_{CREB^p}}}\right)^h + w_{NR4A3_{CREB^p+CRTC}} * \left(\frac{C_{CREB^p}(t-\tau)}{K_{NR4A3_{CREB^p}}}\right)^h * \left(\frac{C_{CRTC}(t-\tau)}{K_{NR4A3_{CRTC}}}\right)^{h_1}}{1 + \left(\frac{C_{CREB^p}(t-\tau)}{K_{NR4A3_{CREB^p}}}\right)^h + w_{NR4A3_{CREB^p+CRTC}} * \left(\frac{C_{CREB^p}(t-\tau)}{K_{NR4A3_{CREB^p}}}\right)^h * \left(\frac{C_{CRTC}(t-\tau)}{K_{NR4A3_{CRTC}}}\right)^{h_1}} * \frac{\left(\frac{C_{CREB^p}(t-\tau)}{K_{NR4A3_{CREB^p}{}_1}}\right)^h}{1 + \left(\frac{C_{CREB^p}(t-\tau)}{K_{NR4A3_{CREB^p}{}_1}}\right)^h}$$

where $K_{NR4A3_{basal}} = 0.25\ \mu M * min^{-1}$ is the basal transcription rate constant; $K_{NR4A3_{CREB^p}} = 5.0E - 5\ \mu M$ and $K_{NR4A3_{CRTC}} = 0.001\ \mu M$ designate effective dissociation constants between the active CREB and CRTC forms and their binding sites of the *NR4A3* gene, respectively, while $K_{NR4A3_{CREB^p}{}_1} = 1.0E - 5\ \mu M$ is the effective dissociation constant between the active CREB form and its alternative binding site of the *NR4A3* gene; $h = h_1 = 2$ correspond to Hill coefficients for *NR4A3* transcription regulation by the active forms of CREB and CRTC factors, respectively; $w_{NR4A3_{CREB^p+CRTC}} = 0.1$ is a constant of the cooperative effect upon an joint impact of CREB and CRTC factors on *NR4A3* transcription. Values of all parameters are identical in both fiber types. The rate of NR4A3 mRNA degradation is described as:

$$F_{NR4A3_{degradation}} = K_{NR4A3_{degradation}} * C_{NR4A3_{mRNA}}$$

where $K_{NR4A3_{degradation}} = 0.07\ min^{-1}$ is the degradation constant the value of which is identical in both fiber types.

The transcription rate of the *X* factor ($F_{X_{transcription}}$) is also described by the same kinetics law as for *NR4A2* gene as well as parameter values are identical in both fiber types:

$$F_{X_{transcription}} = \frac{K_{X_{basal}} + \left(\frac{C_{CREB^p}}{K_{X_{CREB^p}}}\right)^h + w_{X_{CREB^p+CRTC}} * \left(\frac{C_{CREB^p}}{K_{X_{CREB^p}}}\right)^h * \left(\frac{C_{CRTC}}{K_{X_{CRTC}}}\right)^{h_1}}{1 + \left(\frac{C_{CREB^p}}{K_{X_{CREB^p}}}\right)^h + w_{X_{CREB^p+CRTC}} * \left(\frac{C_{CREB^p}}{K_{X_{CREB^p}}}\right)^h * \left(\frac{C_{CRTC}}{K_{X_{CRTC}}}\right)^{h_1}} * \\ * \frac{\left(\frac{C_{CREB^p}}{K_{X_{CREB^p}_1}}\right)^h}{1 + \left(\frac{C_{CREB^p}}{K_{X_{CREB^p}_1}}\right)^h}$$

The biological meaning of the parameters is equal to above-mentioned parameters for other genes, while values of these parameters for *X* factor transcription are:

$K_{X_{basal}} = 0.1\, \mu M * min^{-1}$; $K_{X_{CREB^p}} = 0.005\, \mu M$; $K_{X_{CRTC}} = 0.01\, \mu M$; $K_{X_{CREB^p}_1} = 0.01\, \mu M$; $w_{X_{CREB^p+CRTC}} = 0.1$; $h = h_1 = 2$.

The rate of X mRNA degradation is described as:

$$F_{X_{mRNA_{degradation}}} = K_{X_{mRNA_{degradation}}} * C_{X_{mRNA_{nucleus}}}$$

where $K_{X_{mRNA_{degradation}}} = 0.01\, min^{-1}$ is the degradation constant the value of which is identical in both fiber types. As mentioned above, we also considered processes of the maturation and transport of synthetized X mRNA and protein between nucleus and cytosol compartments. To describe the kinetics of these processes we used a classic first-order kinetic law:

$$F_{X_{mRNA_{processing}}} = K_{X_{mRNA_{processing}}} * C_{X_{mRNA_{nucleus}}}$$

$$F_{transport_{mRNA_{nucl<->cyt}}} = K_{transport_{mRNA_{nucl<->cyt}}} * C_{X_{mRNA_{maturated_{nucleus}}}}$$

$$F_{X_{protein_{translation}}} = K_{X_{protein_{translation}}} * C_{X_{mRNA_{maturated_{cyt}}}}$$

$$F_{transport_{protein_{cyt<->nucl}}} = K_{transport_{protein_{cyt<->nucl}}} * C_{X_{protein_{cyt}}}$$

$$F_{X_{protein_{degradation}}} = K_{X_{protein_{degradation}}} * C_{X_{protein_{nucleus}}}$$

where $C_{X_{mRNA_{nucleus}}}$, $C_{X_{mRNA_{maturated_{nucleus}}}}$, $C_{X_{mRNA_{maturated_{cyt}}}}$, $C_{X_{protein_{cyt}}}$, $C_{X_{protein_{nucleus}}}$ designate concentrations of X mRNA (pre- and maturated mRNAs) and protein in the

corresponding compartments (nucleus or cytosol), while $K_{X_{mRNA_{processing}}} = 0.5\,min^{-1}$, $K_{transport_{mRNA_{nucl<->cyt}}} = K_{transport_{protein_{cyt<->nucl}}} = 0.05\,min^{-1}$, $K_{X_{protein_{translation}}} = 0.01\,min^{-1}$ and $K_{X_{protein_{degradation}}} = 0.01\,min^{-1}$are the rate constant of mRNA processing, transport constants for mRNA and protein between two compartments, the rate constant of the X protein translation and the degradation constant, respectively, values of which are the same in both fiber types.

The maturated X protein is TF candidate regulating transcription of the *PPARGC1α* gene. To consider two alternative and independent regulatory impacts by CREB and X factors the transcription rate of the *PPARGC1α* ($F_{PPARGC1\alpha_{transcription}}$) is described by the next equation:

$$F_{PPARGC1\alpha_{transcription}} = \frac{K_{PPARGC1\alpha_{basal}} + \left(\frac{C_{X_{protein_{nucleus}}}}{K_{PPARGC1\alpha_X}}\right)^h + \left(\frac{C_{CREB^p}}{K_{PPARGC1\alpha_{CREB^p}}}\right)^{h_1}}{1 + \left(\frac{C_{X_{protein_{nucleus}}}}{K_{PPARGC1\alpha_X}}\right)^h + \left(\frac{C_{CREB^p}}{K_{PPARGC1\alpha_{CREB^p}}}\right)^{h_1}}$$

where $K_{PPARGC1\alpha_{basal}} = 0.24\,\mu M * min^{-1}$ is the basal *PPARGC1α* transcription rate constant; $K_{PPARGC1\alpha_X} = 3.5E-4\,\mu M$ and $K_{PPARGC1\alpha_{CREB^p}} = 7.0E-5\,\mu M$ correspond to effective dissociation constants between the active X and CREB forms and their binding sites of the *PPARGC1α* gene, respectively, while $h = h_1 = 2$ designate Hill coefficients for *PPARGC1α* transcription regulation by the active forms of X and CREB factors, respectively. To consider the alternative hypothesis of the transcription regulation by X or CREB factors, the concentration value of the competitive regulator is set to zero.

The rate of PPARGC1α mRNA degradation is described as:

$$F_{PPARGC1\alpha_{degradation}} = K_{PPARGC1\alpha_{degradation}} * C_{PPARGC1\alpha_{mRNA}}$$

where $K_{PPARGC1\alpha_{degradation}} = 0.05\,min^{-1}$ is the degradation constant the value of which is identical in both fiber types.

References:

1. Akberdin, I.R.; Pintus, S.S.; Kolpakov, F.A.; Vertyshev, A.Y.; Popov, D.V. A mathematical model linking Ca^{2+}-dependent signaling pathway and gene expression regulation in human skeletal muscle. *Mathematical Biology and Bioinformatics (Russian)* **2020**, 15(1), pp.20-39.

2. Berdeaux, R. and Hutchins, C. Anabolic and pro-metabolic functions of CREB-CRTC in skeletal muscle: advantages and obstacles for type 2 diabetes and cancer cachexia. *Frontiers in Endocrinology* **2019**, 10, p.535.

3. Birk, J.B; Wojtaszewski, J.F.P. Predominant α2/β2/γ3 AMPK activation during exercise in human skeletal muscle. *The Journal of Physiology* **2006**, 577(3), pp.1021-1032.

4. Coccimiglio, I.F. and Clarke, D.C. ADP is the dominant controller of AMP-activated protein kinase activity dynamics in skeletal muscle during exercise. *PLoS computational biology* **2020**, 16(7), p.e1008079.

5. Cui, J. and Kaandorp, J.A. Simulating complex calcium-calcineurin signaling network. In *International Conference on Computational Science* **2008**, Springer, Berlin, Heidelberg, pp. 110-119.

6. Eilers, W., Gevers, W., Van Overbeek, D., De Haan, A., Jaspers, R.T., Hilbers, P.A., Van Riel, N. and Flück, M.. Muscle-type specific autophosphorylation of CaMKII isoforms after paced contractions. *Biomed research international* **2014**.

7. Kolpakov, F.; Akberdin, I.; Kashapov, T.; Kiselev, L.; Kolmykov, S.; Kondrakhin, Y.; Kutumova, E.; Mandrik, N.; Pintus, S.; Ryabova, A.; Sharipov, R.; Yevshin, I.; Kel, A. BioUML: an integrated environment for systems biology and collaborative analysis of biomedical data. *Nucleic Acids Research* **2019**, 47(W1), pp.W225-W233.

8. Le Novere, N.; Hucka, M.; Mi, H.; Moodie, S.; Schreiber, F.; Sorokin, A.; Demir, E.; Wegner, K.; Aladjem, M.I.; Wimalaratne, S.M.; Bergman, F.T. The systems biology graphical notation. *Nature Biotechnology* **2009**, 27(8), pp.735-741.

9. Li, Y.; Lai, N.; Kirwan, J.P.; Saidel, G.M. Computational model of cellular metabolic dynamics in skeletal muscle fibers during moderate intensity exercise. *Cellular and Molecular Bioengineering* **2012**, 5(1), pp.92-112.

10. Popov, D.V. Adaptation of skeletal muscles to contractile activity of varying duration and intensity: the role of PGC-1α. *Biochemistry (Moscow)* **2018**, 83(6), pp.613-628.

11. Popov, D.V.; Makhnovskii, P.A.; Shagimardanova, E.I.; Gazizova, G.R.; Lysenko, E.A.; Gusev, O.A.; Vinogradova, O.L. Contractile activity-specific transcriptome response to acute endurance exercise and training in human skeletal muscle. *American Journal of Physiology-Endocrinology and Metabolism* **2019**, 316(4), pp.E605-E614.

12. Saucerman, J.J. and Bers, D.M. Calmodulin mediates differential sensitivity of CaMKII and calcineurin to local Ca^{2+} in cardiac myocytes. *Biophysical journal* **2008**, 95(10), pp.4597-4612.

13. Sonntag, A.G., Dalle Pezze, P., Shanley, D.P. and Thedieck, K. A modelling–experimental approach reveals insulin receptor substrate (IRS)-dependent regulation of adenosine monosphosphate-dependent kinase (AMPK) by insulin. *The FEBS journal* **2012**, 279(18), pp.3314-3328.

14. Smith, L.P.; Bergmann, F.T.; Chandran, D.; Sauro, H.M. Antimony: a modular model definition language. *Bioinformatics* **2009**, 25(18), pp.2452-2454.
