# Supplementary material for: A Modular Mathematical Model of Exercise-Induced Changes in Metabolism, Signaling, and Gene Expression in Human Skeletal Muscle
Source: Int J Mol Sci. 2021 Sep 26;22(19):10353. doi: 10.3390/ijms221910353 (PMC8508736; doi:10.3390/ijms221910353)
Supplement: Supplementary file 1 [file ijms-22-10353-s001.zip › ijms-1272147-supplementary (correct file)/Supplementary_Figures_Table.pdf]

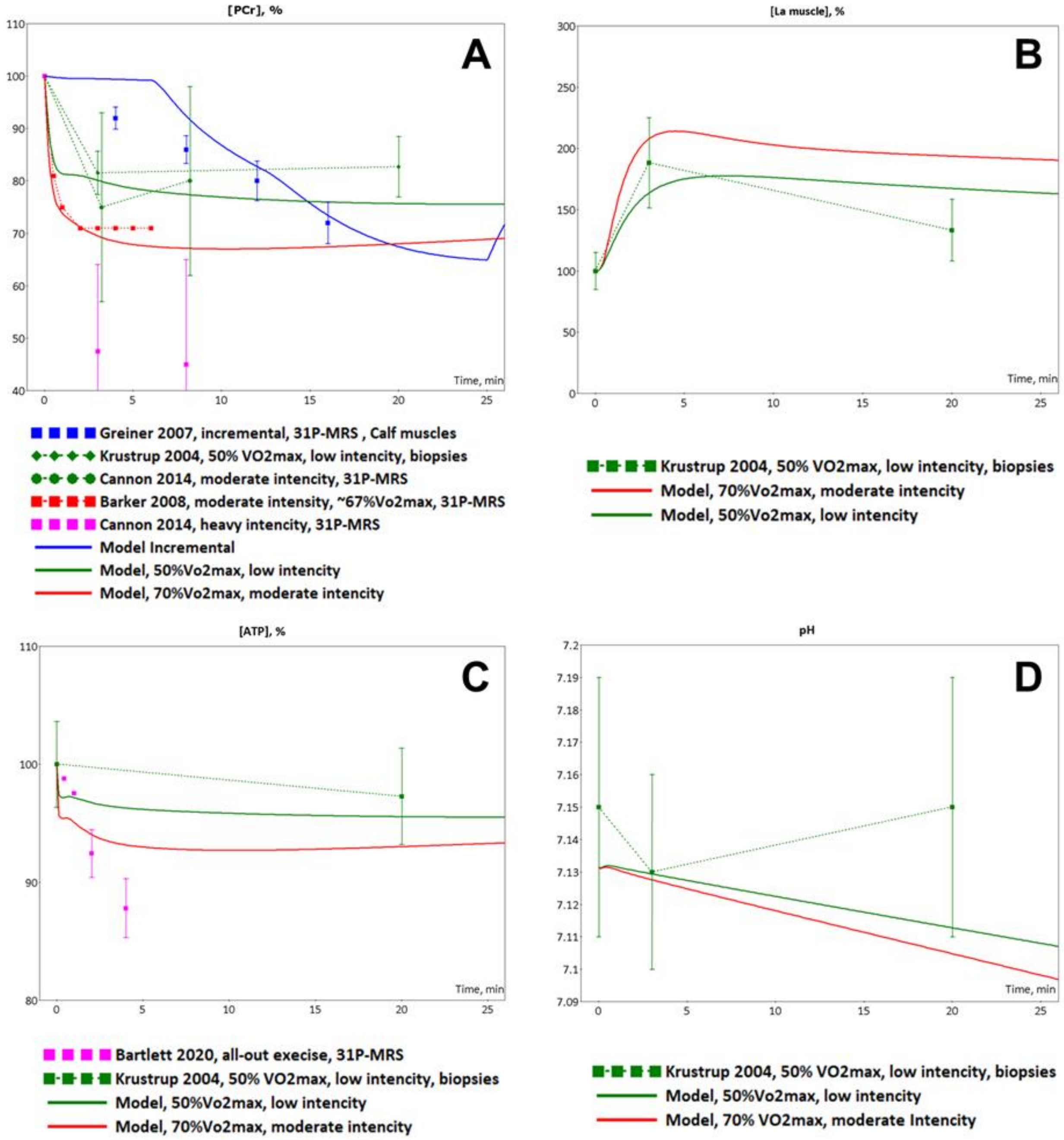

Supplementary Figure S1. Model validation. The model simulations were compared to experimental data. (A) Model-predicted dynamic changes of PCr concentration in the muscle during incremental (blue line), low (green line) and moderate intensity (red line) exercises (axis X - time of the exercise in minutes) and experimental measurements of PCr concentration changes during incremental (blue square dots, [1]), low-intensity (green diamond dots, [2]), green circle dots, [3]), moderate intensity (red square dots, [4]) and high-intensity (magenta square dots, [3]) exercises; (B) Model-predicted dynamic changes of lactate concentration in the muscle during low (green line) and moderate intensity (red line) exercises (axis X - time of the exercise in minutes) and experimental measurements of lactate concentration changes during low-intensity (green square dots, [2]) exercise; (C) Model-predicted dynamic changes of ATP concentration in the muscle during low (green line) and moderate intensity (red line) exercises (axis X - time of the exercise in minutes) and experimental measurements of ATP concentration changes during low-intensity exercise (green square dots, [2]) and all-out exercise (magenta square dots, [5]). The set of experimental data for ATP demonstrates lower [2] and upper [5] boundaries for the concentration; (D) Model-predicted dynamic changes of pH in the muscle during low (green line) and moderate intensity (red line) exercises (axis X - time of the exercise in minutes) and experimental measurements of ATP concentration changes during low-intensity (green square dots, [2]) exercise. Experimental data are shown as mean ± SD.

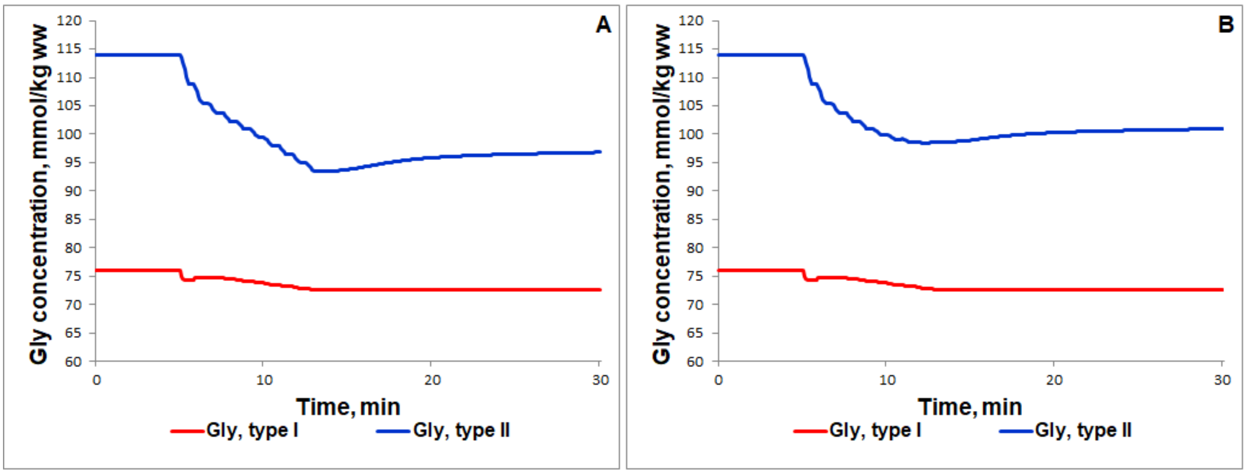

Supplementary Figure S2. Simulation results for high-intensity intermittent exercise (each bout of 30-s exercise separated by 20-s recovery, [6]). (F,G) Glycogen concentration in type I (red) and type II fibers (blue) with constant power generated by type II fibers (Figure 10A) and with successive decline of the power generated by type II fibers (Figure 10C), correspondingly.

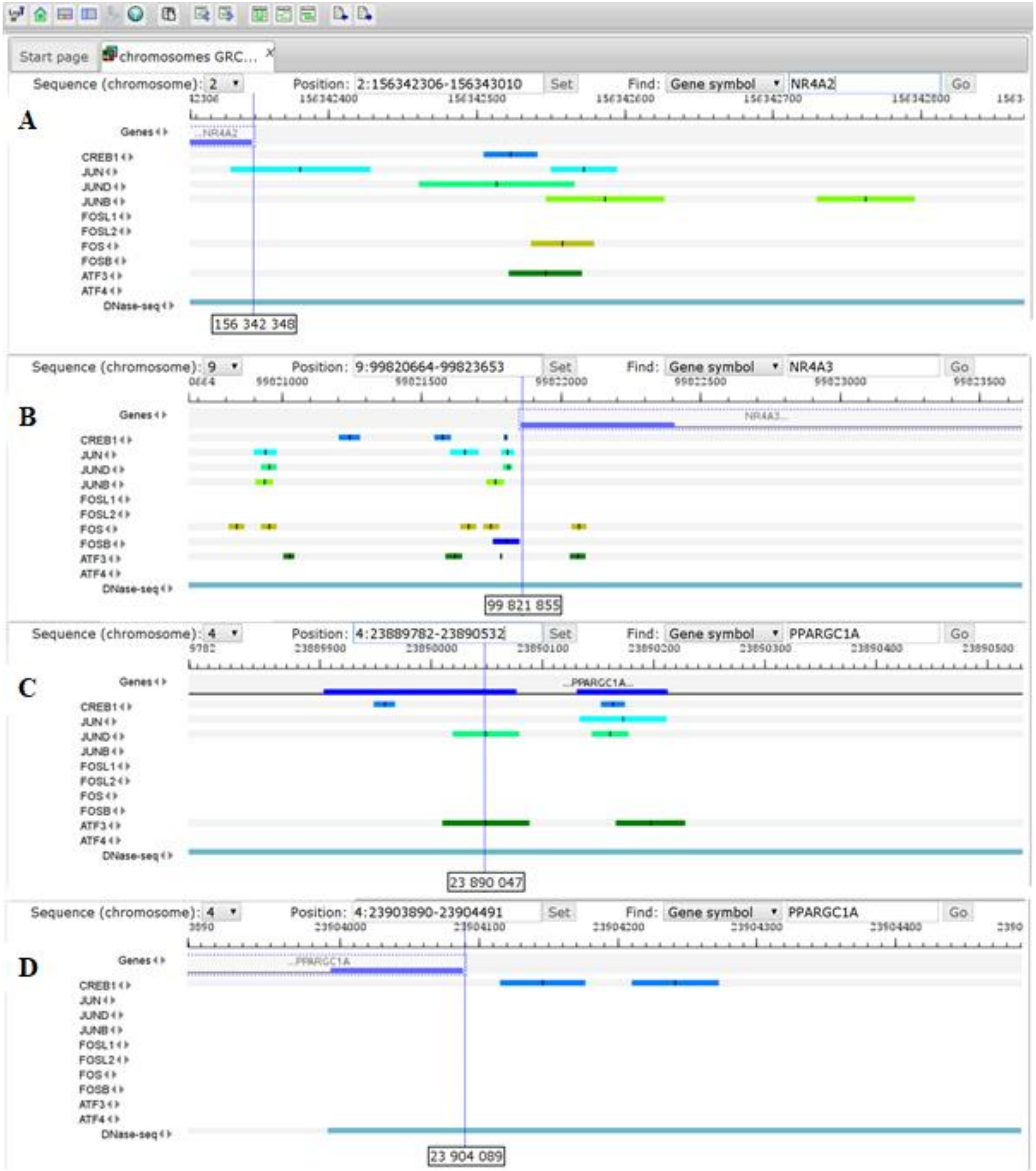

Supplementary Figure S3. Binding sites of transcription factors of CREB, FOS and JUN families in promoter loci of the target genes *NR4A2, NR4A3* and *PPARGC1A*, known from ChIP-seq experiments, as seen in the GTRD genome browser [7]. Transcription start sites are shown with a vertical dashed line; the exact coordinate of each transcription start is given in a box below: A. Promoter region of the *NR4A2* gene (ENSG00000153234). B.Promoter region of the *NR4A3* gene (ENSG00000119508). C. The region of the canonical promoter of the *PPARGC1A* gene (transcript RefSeq ID NM_013261, the coordinate of the transcription start is given according to the NCBI annotation, version 109.20190905). D. The region of the alternative promoter of the *PPARGC1A* gene (transcript RefSeq ID XM_005248132, the coordinate of the transcription start is given according to the NCBI annotation, version 109.20190905)

Supplementary Table S1. Binding sites of transcription factors CREB, FOS and JUN in promoter regions of the target genes _NR4A2_, _NR4A3_ and _PPARGC1A_, known from ChIP-seq experiments (data from the GTRD database).

Transcription factor (TF)	Target gene	Transcription start site (TSS)	TF site location, relative to TSS	Reference
pCREB1	_NR4A2_ (_ENSG00000153234_)	chr2:156342348	-175	[7]
	_NR4A3_ (_ENSG00000119508_)	chr9:99821855	612	[7]
			-279	
			-53	
	_PPARGC1A_* (_XM_005248132_)	chr4:23904089	58	[7]
			-153	
	PPARGC1A# (_NM_013261_)	chr4:23890047	89	[7]
			-117	
JUN	_NR4A2_	chr2:156342348	-224	[7]
			-33	
	_NR4A3_	chr9:99821855	915	[7]
			-200	
			-46	
	_PPARGC1A_*	chr4:23904089		
	PPARGC1A#	chr4:23890047	-126	[7]
JUND	_NR4A2_	chr2:156342348	-166	[7]
	_NR4A3_	chr9:99821855	-901	[7]
			-45	
	_PPARGC1A_*	chr4:23904089		
	PPARGC1A#	chr4:23890047	-3	[7]
			-114	
JUNB	_NR4A2_	chr2:156342348	-239	[7]
			-415	
	_NR4A3_	chr9:99821855	-917	[7]
			-90	
	_PPARGC1A_*	chr4:23904089		
	PPARGC1A#	chr4:23890047		
FOSL1	_NR4A2_	chr2:156342348		
	_NR4A3_	chr9:99821855		
	_PPARGC1A_*	chr4:23904089		
	PPARGC1A#	chr4:23890047		
FOSL2	_NR4A2_	chr2:156342348		
	_NR4A3_	chr9:99821855		
	_PPARGC1A_*	chr4:23904089		
	PPARGC1A#	chr4:23890047		
FOS	_NR4A2_	chr2:156342348	-210	[7]
	_NR4A3_	chr9:99821855	-1017	[7]
			-901	

			-188	
			-105	
			-210	
	PPARGC1A*	chr4:23904089		
	PPARGC1A#	chr4:23890047		
FOSB	NR4A2	chr2:156342348		
	NR4A3	chr9:99821855	50	[7]
	PPARGC1A*	chr4:23904089		
	PPARGC1A#	chr4:23890047		
ATF3	NR4A2	chr2:156342348	-198	[7]
	NR4A3	chr9:99821855	-828	[7]
			-237	
			-69	
			-205	
	PPARGC1A*	chr4:23904089		
	PPARGC1A#	chr2:156342348	-3	[7]
			-151	
ATF4	NR4A2	chr2:156342348		
	NR4A3	chr9:99821855		
	PPARGC1A*	chr4:23904089		
	PPARGC1A#	chr4:23890047		

* – alternative promoter of the *PPARGC1A*; # – canonical promoter of the *PPARGC1A*.

References

1. Greiner, A.; Esterhammer, R.; Bammer, D.; Messner, H.; Kremser, C.; Jaschke, W.R.; Fraedrich, G.; Schocke, M.F. High-energy phosphate metabolism in the calf muscle of healthy humans during incremental calf exercise with and without moderate cuff stenosis. *European Journal of Applied Physiology* **2007**, 99(5), pp.519-531.
2. Krustrup, P.; Soderlund, K.; Mohr, M.; Bangsbo, J. Slow-Twitch Fiber Glycogen Depletion Elevates Moderate-Exercise Fast-Twitch Fiber Activity and O~ 2 Uptake. *Medicine and Science in Sports and Exercise* **2004**, 36(6), pp. 973-982.
3. Cannon, D.T.; Bimson, W.E.; Hampson, S.A.; Bowen, T.S.; Murgatroyd, S.R.; Marwood, S.; Kemp, G.J.; Rossiter, H.B. Skeletal muscle ATP turnover by 31P magnetic resonance spectroscopy during moderate and heavy bilateral knee extension. *The Journal of Physiology* **2014**, 592(23), pp.5287-5300.
4. Barker, A.R.; Welsman, J.R.; Fulford, J.; Welford, D.; Armstrong, N. Muscle phosphocreatine kinetics in children and adults at the onset and offset of moderate-intensity exercise. *Journal of Applied Physiology* **2008**, 105(2), pp.446-456.
5. Bartlett, M.F.; Fitzgerald, L.F.; Nagarajan, R.; Hiroi, Y.; Kent, J.A. Oxidative ATP synthesis in human quadriceps declines during 4 minutes of maximal contractions. *The Journal of Physiology* **2020**, 598(10), pp.1847-1863.
6. Kappenstein, J.; Ferrauti, A.; Runkel, B.; Fernandez-Fernandez, J.; Müller, K.; Zange, J. Changes in phosphocreatine concentration of skeletal muscle during high-intensity intermittent exercise in children and adults. *European Journal of Applied Physiology* **2013**, 113(11), pp.2769-2779.
7. Kolmykov, S.; Yevshin, I.; Kulyashov, M.; Sharipov, R.; Kondrakhin, Y.; Makeev, V.J.; Kulakovskiy, I.V.; Kel, A.; Kolpakov, F. GTRD: an integrated view of transcription regulation. *Nucleic Acids Research* **2021**, 49(D1), pp.D104-D111.
