# Supplementary figures and images for: A Modular Mathematical Model of Exercise-Induced Changes in Metabolism, Signaling, and Gene Expression in Human Skeletal Muscle
Source: Int J Mol Sci. 2021 Sep 26;22(19):10353. doi: 10.3390/ijms221910353 (PMC8508736; doi:10.3390/ijms221910353)

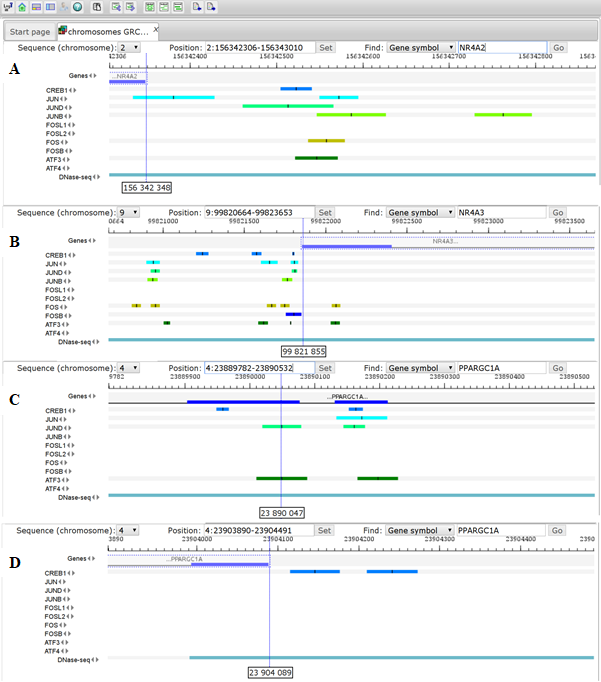

Supplement: Supplementary file 1 [file ijms-22-10353-s001.zip › ijms-1272147-supplementary (correct file)/Binding sites_GTRD.png]

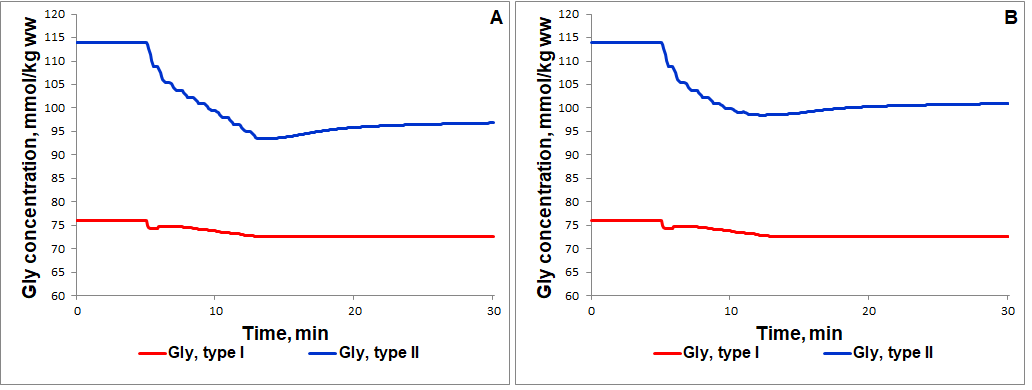

Supplement: Supplementary file 1 [file ijms-22-10353-s001.zip › ijms-1272147-supplementary (correct file)/Glycogen concentration.png]

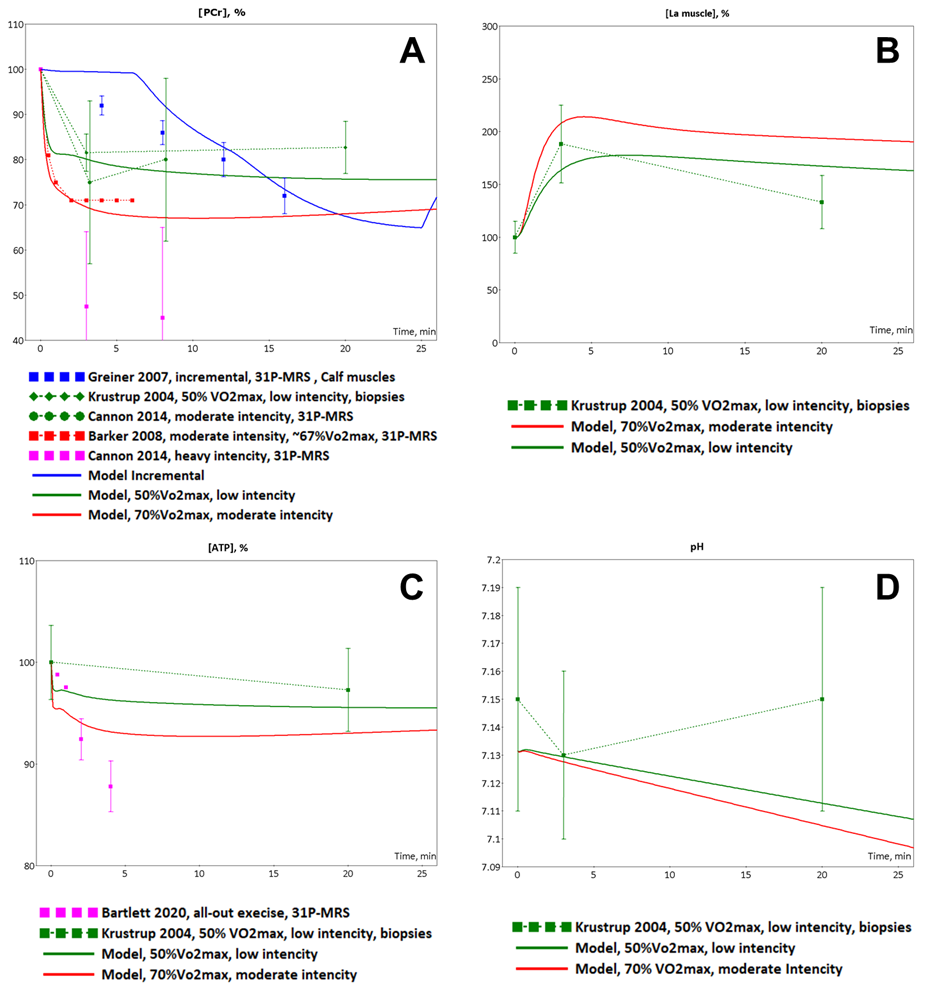

Supplement: Supplementary file 1 [file ijms-22-10353-s001.zip › ijms-1272147-supplementary (correct file)/Validation.png]
